# Rational Design of Advanced Functional Catalysts for Photo‐Reforming Glucose into Bio‐Derived Fuels and Chemicals

**DOI:** 10.1002/advs.202508850

**Published:** 2025-07-13

**Authors:** Qiong Yan, Yuyue Zhou, Yan Zhang, Dalin Sun, Xu Wu, Song Yang, Chunbao Xu, Heng Zhang

**Affiliations:** ^1^ State Key Laboratory of Green Pesticide State‐Local Joint Laboratory for Comprehensive Utilization of Biomass Center for R&D of Fine Chemicals of Guizhou University Guiyang 550025 China; ^2^ School of Energy and Environment City University of Hong Kong Kowloon Tong 999077 Hong Kong

**Keywords:** bio‐based chemicals, bio‐fuels, catalyst design, glucose, photo‐reforming

## Abstract

Transforming renewable biomass resources into high‐value chemicals and energy‐dense fuels has become paramount for advancing sustainable economic development toward a carbon‐neutral society. Glucose, a key platform molecule for biomass, has attracted considerable research attention for its promise as a feedstock for the production of various biochemicals and biofuels. To achieve glucose conversion with enhanced selectivity and efficiency, it is critical to develop high‐performance heterogeneous photocatalysts and fine‐tune cascade catalytic processes. This review provides a systematic overview of recent progress in design of advanced functional catalysts for photo‐reforming glucose into bio‐derived fuels and a variety of bio‐based molecules, with an emphasis on design and applications of functionalized heterogeneous photocatalysts, including material structure optimization strategies and their impacts on catalytic performance. Furthermore, this review covers key aspects such as reaction mechanism elucidation, product selectivity control, and reaction condition optimization, providing comprehensive insights into structure‐activity relationships and functional modification strategies. Through a systematic analysis of the current research landscape, this review outlines the rational design principles for functional catalysts and the optimization strategies for catalytic processes. It aims to provide theoretical guidance for the development of efficient and stable photocatalytic systems and to offer new solutions to producing green chemicals/fuels and achieving carbon neutrality.

## Graphical abstract

This review systematically summarizes the fundamental mechanisms of photocatalytic glucose conversion, catalyst design strategies, key reaction pathways, structure‐activity relationships, influence of reaction parameter adjustments, and current challenges in the field. It emphasizes the structural optimization of semiconductor photocatalysts, such as metal oxides, sulfides, and carbon‐based materials, and their role in achieving selective product formation. Additionally, it explores future research directions, including the development of high‐performance visible light‐responsive catalysts, advancements in interface engineering, and improvements in catalytic stability.

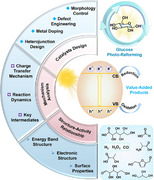

## 1 Introduction

**Figure 1:**
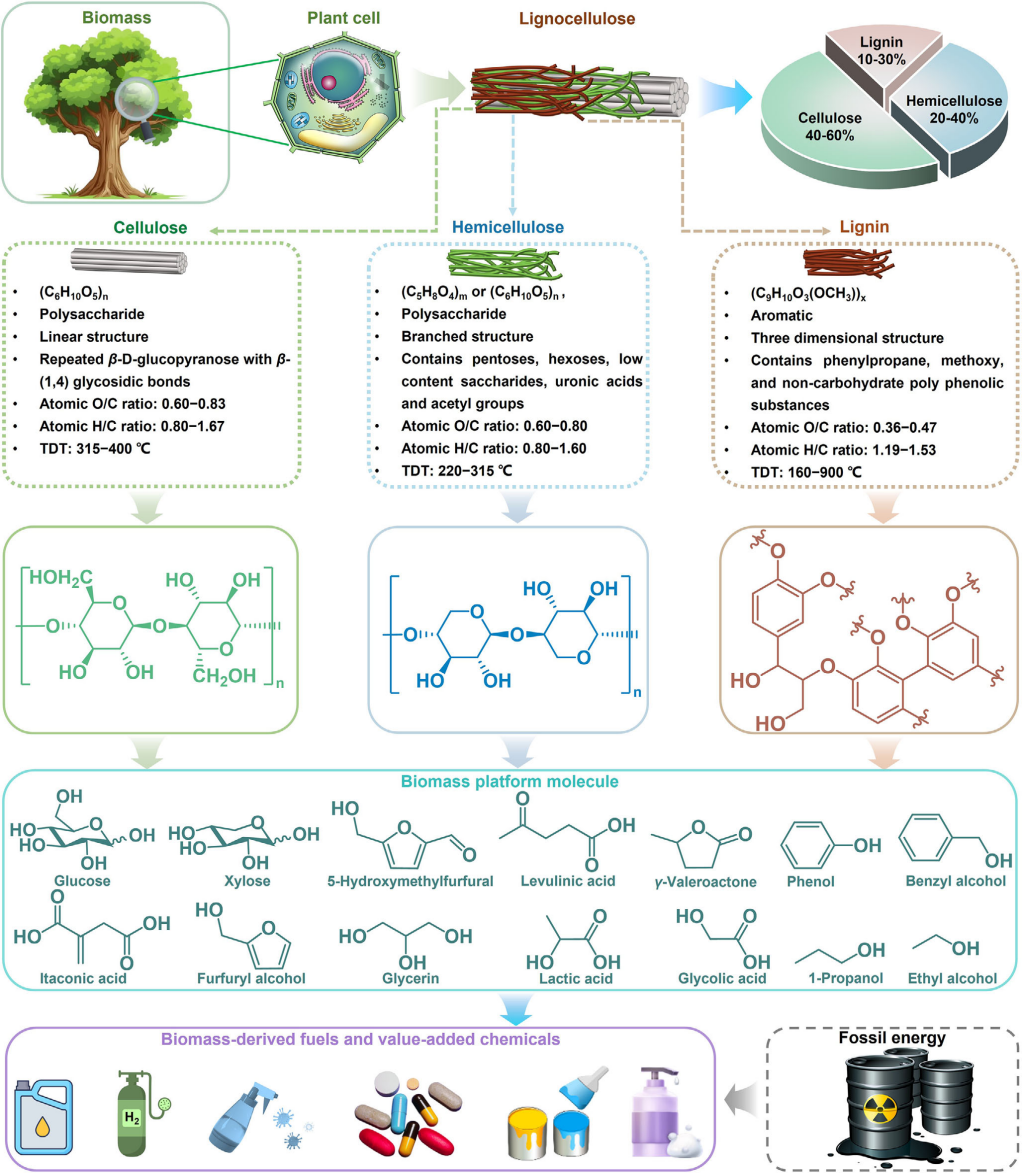
Schematic diagram of bio‐fuels and chemicals production from lignocellulosic biomass.

For decades, coal, petroleum, and natural gas have served as the dominant global energy sources and key feedstock for the production of various essential chemicals.^[^ ^1^, ^2^ ^]^ However, the excessive consumption of fossil fuels has not only accelerated resource depletion but also caused severe environmental issues, including greenhouse gas emissions, air pollution, and ecosystem degradation.^[^ ^3^, ^4^ ^]^ Under the dual pressure of intensifying global warming and rising energy demand, both the scientific and industrial communities are actively exploring sustainable alternatives to conventional fossil fuels.^[^ ^5^ ^]^ In recent years, biomass, solar, and wind power have attracted considerable interest as renewable energy sources, owing to their rapid regeneration, reliable supply, and eco‐friendly characteristics.^[^ ^1^ ^]^ These renewables are now regarded as essential components of future energy systems. Among them, biomass, the fourth most abundant resource on the Earth, is widely available from various sources such as agricultural and forestry residues, wood, aquatic plants, and animal waste.^[^ ^6^ ^]^ More importantly, biomass is the only renewable resource for carbon‐based fuels and chemicals.^[^ ^7^ ^]^ Lignocellulose, primarily composed of cellulose, hemicellulose, and lignin, is one of the most abundant biomass resources.^[^ ^5^, ^7^, ^8^ ^]^ Through well‐designed catalytic conversion technologies, lignocellulose can be efficiently transformed into clean fuels and high‐value chemicals, playing a crucial role in advancing a low‐carbon economy and sustainable development (**Figure** **1**). This transformation helps to mitigate our society's reliance on conventional fossil resources, laying the foundation for a green and circular system economy.^[^ ^3^ ^]^

**Figure 2:**
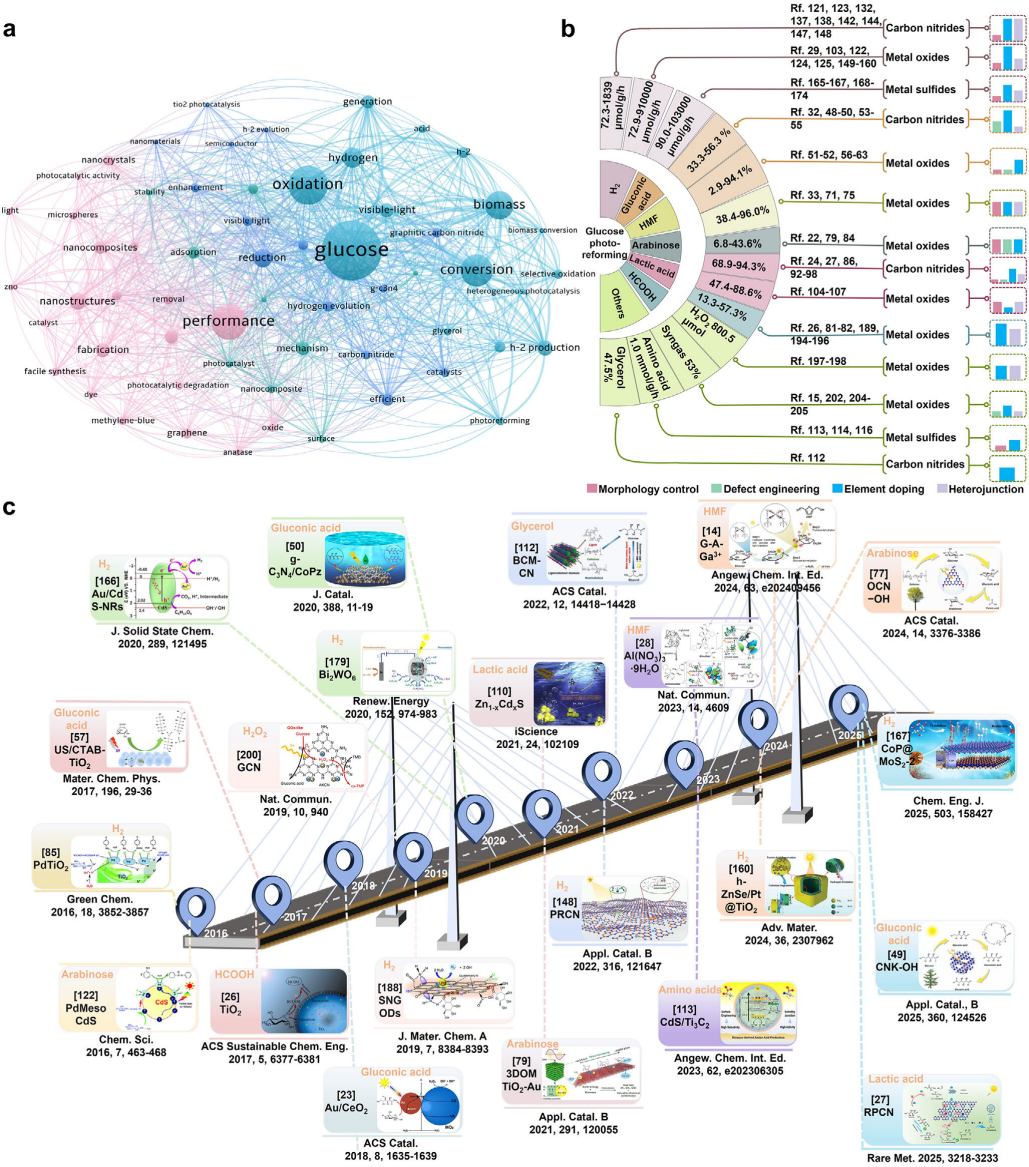
a) Visual analysis of glucose photo‐reforming, the size of the circle corresponds to keyword frequency, and the thickness of the line represents connection strength. b) Development of nanostructured semiconductor catalysts for photo‐reforming of glucose. c) A timeline of glucose photocatalytic reforming.

Glucose, the primary building block for cellulose, acts as both a crucial intermediate and a final product in its conversion process.^[^ ^9^ ^]^ With its widespread availability and high reactivity, glucose is regarded as a key platform molecule in biomass valorization.^[^ ^10^ ^]^ The efficient catalytic conversion of glucose into premium chemicals and clean energy represents a pivotal strategy for sustainable industrial development.^[^ ^11^ ^]^ Photocatalytic technology, with its unique ability to harness solar energy to drive chemical reactions under mild conditions, offers distinct advantages for biomass valorization.^[^ ^12^, ^13^ ^]^ In recent years, research on the photocatalytic conversion of glucose has achieved remarkable progress, with its applications continuously expanding in diversified fields (**Figure** **2**a,b). The past decade has witnessed remarkable progress in photocatalytic glucose reforming toward bio‐fuels and value‐added platform molecules, significantly advancing the valorization of biomass resources (Figure 2c). Compared to conventional thermo‐catalytic approaches, which often operate at high temperatures and pressures, photocatalysis operates effectively at ambient conditions. This key advantage contributes to environmental sustainability by minimizing energy consumption and reducing the need for harsh chemicals or solvents typically used in thermal processes. Additionally, photocatalytic glucose conversion allows for a more controllable and selective transformation of glucose, which results in fewer by‐products and improved product yields.^[^ ^14^, ^15^ ^]^ As a result, it presents a promising strategy for the high‐value utilization of biomass resources.

**Figure 3:**
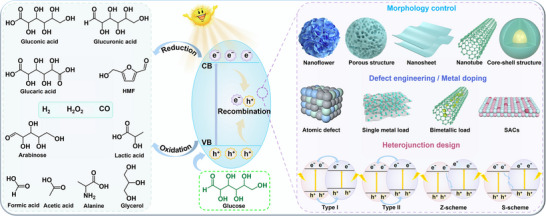
Schematic illustration of producing a variety of bio‐based fuels and platform chemicals from glucose by photocatalysis.

Achieving efficient and controllable glucose valorization relies on the development of functionalized heterogeneous catalysts. Cascade catalysis, which enables multiple consecutive reactions within a single system, offers an effective strategy for glucose deep conversion.^[^ ^16^, ^17^ ^]^ Heterogeneous catalysts are widely used for cascade catalysis due to their recyclability, high stability, and tunable structural properties.^[^ ^18^ ^]^ Rational surface functionalization design can optimize catalytic performance, enhancing reaction selectivity and efficiency.^[^ ^16^, ^18^ ^]^ Furthermore, advancements in materials science and catalytic technology have introduced innovative approaches for designing novel catalytic materials.^[^ ^19^, ^20^ ^]^ To date, various effective strategies such as morphology control, defect engineering, metal doping, and heterostructure construction have been successfully applied to design and realize efficient photo‐reforming of glucose (**Figure** **3**). With unique electronic structures and surface chemistry, these catalysts efficiently absorb solar energy and generate electron‐hole (eˉ‐h^+^) pairs, facilitating glucose transformation into high‐value‐added chemicals.^[^ ^21^, ^22^ ^]^ They include bio‐fuels (H_2_ and syngas), organic acids (e.g., gluconic acid, lactic acid, and formic acid (HCOOH)), sugar‐derived compounds (arabinose), and biobased platform chemicals such as 5‐hydroxymethylfurfural (HMF).^[^ ^23^, ^24^, ^25^, ^26^, ^27^, ^28^ ^]^ Renewable H_2_, among these products, holds great promise as a clean energy carrier and chemical feedstock.^[^ ^29^, ^30^, ^31^ ^]^ Gluconic acid and lactic acid have broad applications across food, pharmaceutical, and chemical sectors, while HMF serves as a key precursor for bioplastics and renewable fuels.^[^ ^32^, ^33^ ^]^ Interestingly, the global glucose market was valued at United States dollar (USD) 900 million in 2024 and is projected to reach USD 1.09 billion by 2029, exhibiting a compound annual growth rate (CAGR) of 3.7%. Furthermore, the CAGRs for H_2_, gluconic acid, HMF, arabinose, formic acid, lactic acid, H_2_O_2_, syngas, amino acids, and glycerol were determined to be 7.8%, 7.2%, 3.5%, 10.4%, 4.5%, 12.4%, 6.0%, 10.2%, 7.7%, and 11.9%, respectively. The market data cited in this study were sourced from two authoritative market research and consulting companies: Markets and Markets, a leading global market research firm, and Global Market Insights, a leading market intelligence provider. The efficient production of these high‐value compounds not only enhances the economic value of biomass resources but also advances green chemistry, offering new solutions for sustainable energy and materials development.

Existing reviews on glucose valorization have predominantly examined catalyst design and reaction engineering approaches for the synthesis of platform chemicals and biofuels.^[^ ^34^, ^35^, ^36^ ^]^ However, there remains a lack of comprehensive and systematic reviews specifically addressing glucose photo‐reforming, particularly in terms of catalyst types, performance enhancement strategies, reaction microenvironments, and structure‐activity relationships (**Table** **1**). To elucidate the impact of catalyst design and system optimization on biofuel production and value‐added chemicals synthesis through glucose photo‐reforming, this review serves a comprehensive summary and critical analysis on the applications of advanced photocatalysts, including carbon‐nitrogen materials, metal oxides, metal sulfides, single atom catalysts (SACs), and heterojunction catalysts for glucose conversion. Through critical examination of catalyst design principles and reaction pathway modulation mechanisms, this review bridges the knowledge gap between fundamental materials science and photocatalytic biomass conversion engineering, but also evaluates persistent technological bottlenecks. This work accelerates the transition of photocatalytic glucose conversion from conceptual research to practical implementation, advancing sustainable biorefining technologies.

**Table 1:** Comparison of the recent reviews on glucose photo‐reforming to this work.

Refs.	Catalyst types	Catalyst design strategies	Performance improvement strategies	Reaction mechanism	Products regulation	Reaction microenvironments	Structure‐activity relationships
[4]	x	√	x	√	x	√	x
[21]	x	√	x	√	x	√	x
[31]	x	x	√	√	x	x	√
[34]	x	√	x	√	√	x	√
[35]	x	x	√	√	x	x	x
[36]	x	√	x	√	x	x	√
**This work**	**√**	**√**	**√**	**√**	**√**	**√**	**√**

## 2 Application Field

**Figure 4:**
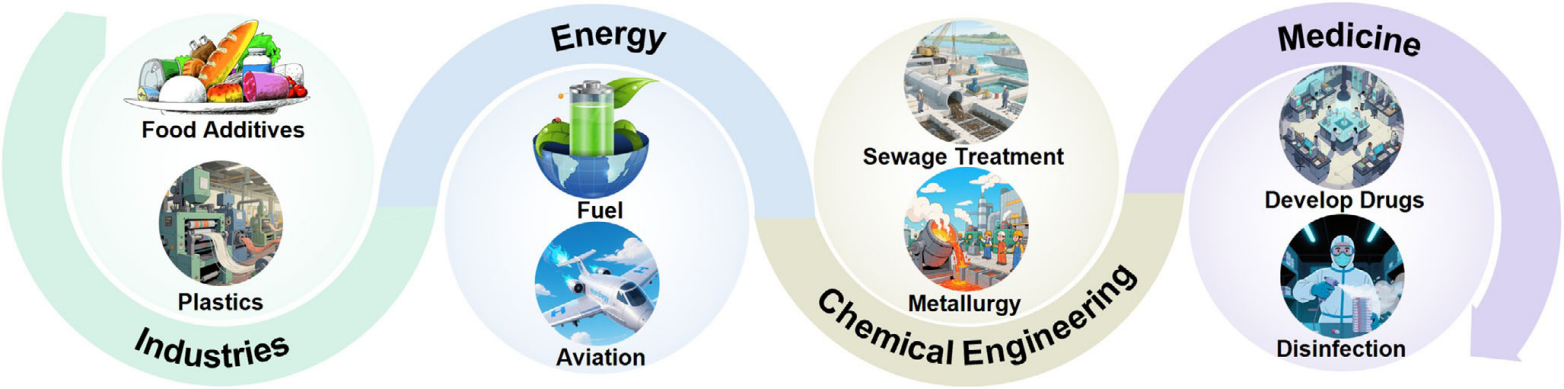
The application fields of glucose and its derivatives.

As a fundamental biomass platform molecule, glucose and its derived functional molecules have established diverse application systems in industries, energy, chemical engineering, and medicine, making photocatalytic conversion technology crucial for promoting their green valorization (**Figure** **4**).^[^ ^37^ ^]^

In the industrial and material sectors, glucose serves as a reducing agent in the dyeing and tanning industries, and as a carbon source in wastewater treatment, while being chemically converted into functional materials like polydextrose. Gluconic acid and its sodium salt exhibit exceptional chelating and corrosion inhibition properties, functioning as key agents in metal surface treatment, electroplating, and circulating water systems. In food applications, gluconates regulate acidity and replace sodium salts to meet the needs of low‐sodium diets.^[^ ^38^ ^]^ HMF bridges biomass to high‐performance materials: Its oxidation product 2,5‐furandicarboxylic acid, substitutes petroleum‐based terephthalic acid for synthesizing biodegradable polyester poly(ethylene furanoate) for green packaging and industrial films.^[^ ^39^ ^]^ HMF‐derived aldol condensation products yield C7–C15 liquid alkanes as biofuels, while rehydration generates levulinic acid, a pivotal energy platform molecule.^[^ ^40^ ^]^ Lactic acid polymerizes into polylactic acid (PLA), a biodegradable and biobased material replacing conventional plastics. Glycerol (a precursor for nitroglycerin and resins) and arabinose (a low glycemic index sweetener) further extend the derivative applications in chemicals, pharmaceuticals, and food industries.^[^ ^41^ ^]^

In the energy and environmental sectors, H_2_, HCOOH, and syngas constitute the core of clean energy systems. H_2_ powers fuel cells in transportation and stores intermittent renewable energy. HCOOH serves as a safe hydrogen carrier for storage and transport, while syngas converts to liquid fuels via Fischer–Tropsch synthesis, supporting transportation decarbonization. Meanwhile, H_2_O_2_, a green oxidant and aerospace fuel, links to glucose conversion through photocatalytic production, enhancing its sustainability.^[^ ^42^, ^43^ ^]^

Traditional glucose conversion relies on high‐temperature, high‐pressure processes with strong oxidants, suffering from high energy consumption and pollution. Photocatalysis, driven by solar energy under mild conditions, offers a green pathway for directing glucose to value‐added chemicals and energy molecules.

## 3 Bio‐Based Molecules

This chapter provided a comprehensive review of the photocatalytic production of gluconic acid, HMF, arabinose, lactic acid, glycerol, and amino acids from glucose. It systematically summarized the key governing factors in these processes, including reaction mechanisms, active sites, catalyst structure‐activity relationships, and strategies for product selectivity control.

### 3.1 Gluconic Acid

Preparation of gluconic acid by photo‐oxidation of glucose is an environmentally friendly and highly efficient process. Gluconic acid, a crucial organic acid, has extensive applications across various fields, including food, pharmaceuticals, chemical engineering, environmental protection, and agriculture.^[^ ^32^, ^44^, ^45^ ^]^ For example, gluconic acid and its salts can function as additives, humectants, and preservatives in the food industry.^[^ ^46^ ^]^ In the pharmaceutical sector, gluconic acid exhibits bioactivities such as antibacterial and anti‐inflammatory properties, making it suitable for the development of novel drugs.^[^ ^41^, ^47^ ^]^ In the chemical industry, gluconic acid serves as an important precursor for synthesizing various high‐value‐added chemicals.^[^ ^34^ ^]^ Therefore, photocatalytic glucose oxidation is a sustainable chemical process and provides new avenues for the broad application of gluconic acid.

#### 3.1.1 Mechanism

**Figure 5:**
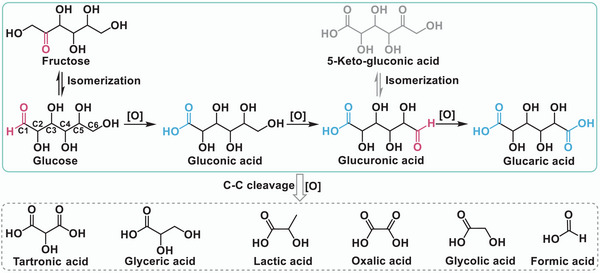
Reaction mechanism of photocatalytic conversion of glucose to gluconic acid and other derivatives. Reproduced with permission.^[^ ^34^ ^]^ Copyright 2021, Elsevier.

The photocatalytic oxidation of glucose proceeds via a stepwise mechanism (**Figure** **5**). Primary oxidation preferentially targets the ─CHO group at C1, yielding gluconic acid as the principal product. Subsequent secondary oxidation attacks the ─OH group at C6, forming an aldehyde group and ultimately producing glucuronic acid, which can be further oxidized to glucaric acid.^[^ ^48^ ^]^ Notably, the yield of glucuronic acid remains substantially lower than that of gluconic acid due to the higher activation energy barrier associated with alcohol oxidation compared to aldehyde group conversion.^[^ ^49^ ^]^ Moreover, glucuronic acid is prone to isomerization, which yields 5‐keto‐gluconic acid.^[^ ^32^, ^50^ ^]^ In addition, several side reactions occur during glucose oxidation to gluconic acid, including isomerization, C─C bond cleavage, and overoxidation. Small‐molecule carboxylic acids are produced through C─C bond cleavage and oxidation of aldehyde intermediates.^[^ ^51^ ^]^ Furthermore, glucose can reversibly isomerize into fructose under alkaline conditions. This isomerization can also occur in neutral settings when H_2_O_2_ is added.^[^ ^52^ ^]^

#### 3.1.2 Catalyst Design

Photocatalytic glucose oxidation to gluconic acid requires precise C1/C6 position selectivity, effectively suppressing competitive C−C bond scission that generates undesirable side products. Therefore, designing catalysts with high selectivity for C1 and C6 oxidation is crucial to achieving efficient and controlled glucose conversion.

##### Graphitic carbon nitride (g‐C_3_N_4_)

**Figure 6:**
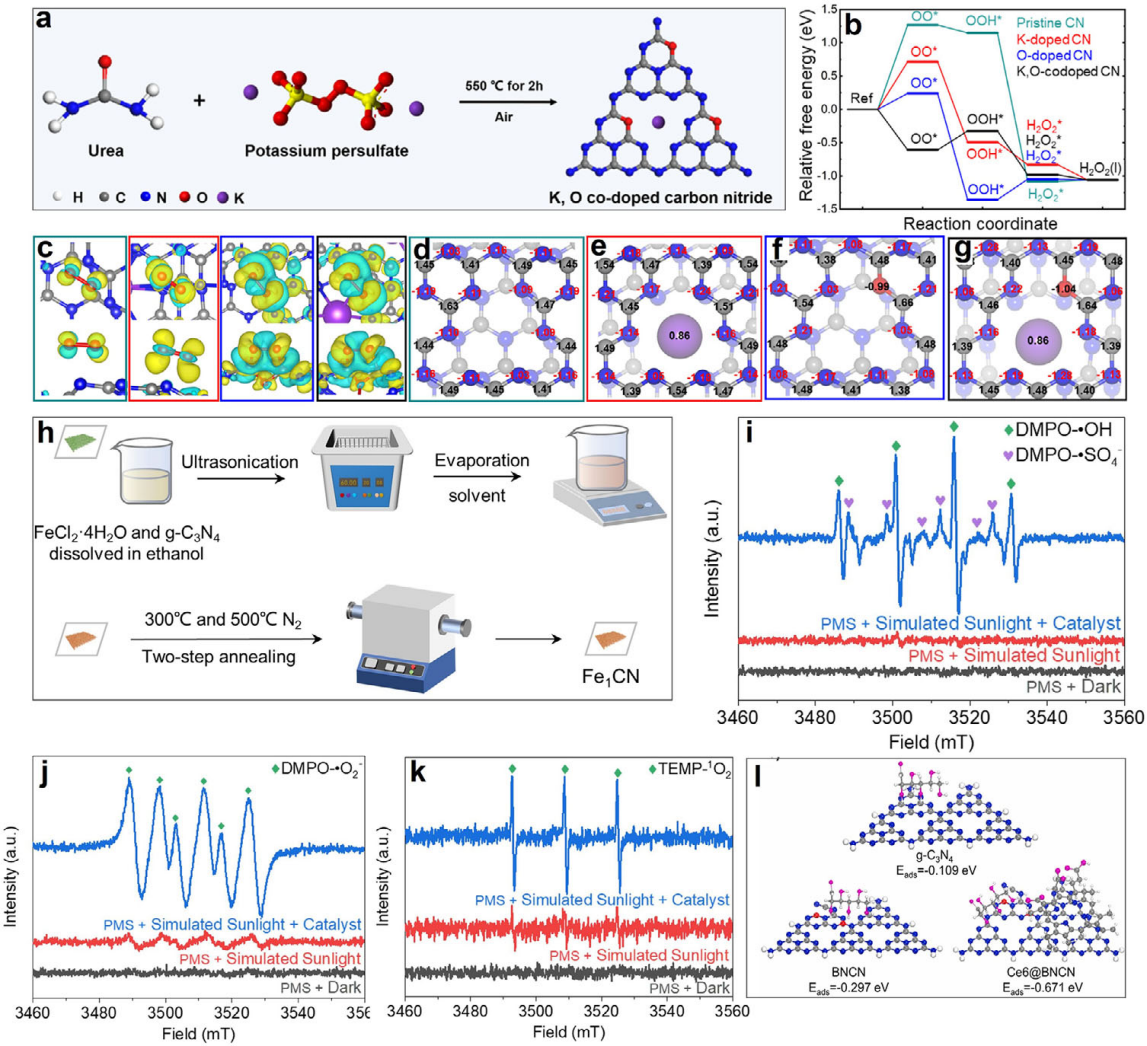
a) Schematic diagram of the synthesis of K, O co‐doped red CN by thermal copolymerization. b) Gibbs free energy of 2eˉ‐ORR of modified CN. c) Charge density difference of adsorbed O_2_ on CN, K‐doped CN, O‐doped CN, and K, O co‐doped CN. d–g) Bader charge of CN, K‐doped CN, O‐doped CN, and K, O co‐doped CN. Reproduced with permission.^[^ ^32^ ^]^ Copyright 2023, American Chemical Society. h) Synthesis technology of Fe1CN catalyst. EPR spectra of the i) DMPO‐•OH/SO•ˉ 4, j) DMPO‐•O_2_ˉ, and k) TEMP‐^1^O_2_. Reproduced with permission.^[^ ^53^ ^]^ Copyright 2024, Elsevier. l) Adsorption energy of glucose on g‐C_3_N_4_, BNCN, and Ce6@BNCN. Reproduced with permission.^[^ ^48^ ^]^ Copyright 2022, Elsevier.

Efficiently converting glucose into high‐value‐added gluconic acid through a photocatalytic Fenton‐like system was essential for addressing the trade‐off between activity and selectivity in traditional photocatalytic processes.^[^ ^53^ ^]^ The catalyst red carbon nitride (RCN), derived from K and O co‐doping of g‐C_3_N_4_ (**Figure** **6**a), exhibited dual functionality: 1) catalytic H_2_O_2_ synthesis via 2eˉ oxygen reduction reaction (2eˉ‐ORR), followed by 2) photo‐Fenton mediated decomposition into hydroxyl radicals (•OH).^[^ ^32^ ^]^ Notably, density functional theory (DFT) calculations validated that K and O co‐doped CN enhanced in generating •O_2_ˉ and H_2_O_2_ (Figure 6b). This enhancement was attributed to the reduced repulsion between N and O in the co‐doped CN, leading to a more favorable O_2_ adsorption configuration on RCN (Figure 6c). Additionally, the charge redistribution in RCNs resulted in enhanced light utilization and charge transfer (Figure 6d–g).^[^ ^32^ ^]^ Within this framework, a Fenton‐like system utilizing Fe single atoms (SAs) loaded carbon nitride (Fe_1_CN) as the catalyst and peroxymonosulfate (PMS) as the oxidant demonstrated significant potential (Figure 6h).^[^ ^53^ ^]^ Fe SAs integration into g‐C_3_N_4_ engineered the material's electronic band configuration, broadening its spectral response while utilizing Fe active centers for effective PMS activation. This system generated various reactive oxygen species (ROS) and free radicals, including •OH, SO• 4ˉ, •O_2_ˉ, and ^1^O_2_, with ^1^O_2_ playing a key role in selectively oxidizing glucose (Figure 6i–k).^[^ ^53^ ^]^ By optimizing the surface properties and electronic structure of the catalyst, this approach promoted glucose adsorption and activation, steering the reaction toward gluconic acid formation while minimizing over‐oxidation byproducts, achieving an impressive gluconic acid selectivity of 91.6%.

The photocatalytic performance of glucose oxidation was significantly enhanced through a dual modification strategy involving N defect engineering and chlorophyll e6 (Ce6) integration. Initially, N‐deficient g‐C_3_N_4_ (BNCN) served as the substrate, which was subsequently functionalized with Ce6 to form the Ce6@BNCN composite. This hierarchical design amplified light harvesting efficiency and promoted charge carrier separation (Figure 6l).^[^ ^48^ ^]^ When employing H_2_O_2_ as the oxidant, the optimized catalyst achieves 70.9% gluconic acid selectivity in glucose photooxidation, attributable to two synergistic effects: 1) The Ce6‐BNCN interfacial interaction strengthened glucose adsorption capacity compared to pristine g‐C_3_N_4_ and BNCN, accelerating substrate activation; 2) Conversely, weakened adsorption of oxidation products including gluconic acid and glucaric acid, facilitated their desorption, thereby minimizing overoxidation and improving selectivity.^[^ ^48^ ^]^ Mechanistic studies reveal that H_2_O_2_ generates ROS, including •OH, O_2_ˉ, and ^1^O_2_, which collectively drive glucose oxidation.^[^ ^50^ ^]^ Notably, incorporating cobalt tetra(2,3‐bisbutylthio) maleonitrile tetrazaporphyrin (CoPz) into g‐C_3_N_4_ modulates ROS profile by selectively suppressing •OH formation. This targeted inhibition enhanced reaction specificity while mitigating undesirable side pathways.^[^ ^50^ ^]^

Alkalinization modification was another effective strategy for enhancing g‐C_3_N_4_.^[^ ^49^ ^]^ The modified carbon nitride enhanced visible light absorption and charge separation while also facilitating oxygen activation, resulting in increased ROS production. Meanwhile, 2,2,6,6‐tetramethyl‐1‐piperidinyloxy (TEMPO) acted as an oxidizing agent, enhancing the oxidation efficiency of primary alcohol groups at the C6 position and enabling the highly selective photocatalytic conversion of glucose to glucaric acid.^[^ ^49^ ^]^ Notably, TEMPO could be regenerated through redox cycling during the photocatalytic process, ensuring sustained catalytic activity. Alternatively, alkalinization modification of carbon nitride could be achieved by incorporating KOH and KCl.^[^ ^54^ ^]^ Transient photoluminescence (TRPL) spectroscopy analysis showed a substantial reduction in the fluorescence lifetime of alkalinized g‐C_3_N_4_ (from 18.42 to 3.65 ns), suggesting enhanced separation of photogenerated charge carriers. The improved photocatalytic activity was ascribed to the hollow stacked nanosheet architecture of alkali‐modified carbon nitride, which expanded the specific surface area and promoted charge carrier mobility and reactant adsorption.^[^ ^49^ ^]^ Additionally, Wang et al. further enhanced the properties of carbon nitride by co‐doping it with K and S to develop bifunctional photocatalysts.^[^ ^55^ ^]^ This co‐doping strategy enabled the simultaneous generation of gluconic acid and syngas in acidic media.

The reported studies collectively demonstrated that modification of g‐C_3_N_4_, typically through elemental doping, was primarily designed to enhance its oxygen activation capacity and ROS generation capability. This optimization was particularly crucial for selective oxidation processes, as the oxidation reactions predominantly involve the ─CHO or ─CH_2_OH functional groups at glucose termini.

##### TiO_2_

Pristine TiO_2_ demonstrates photocatalytic activity for glucose conversion under visible irradiation, yielding gluconic acid (7% selectivity) alongside arabinose as oxidation products.^[^ ^51^ ^]^ Due to the ligand‐to‐metal charge transfer (LMCT) mechanism, TiO_2_ exhibits visible‐light activity when glucose adsorbs on its surface, forming an electron‐transfer complex that injects electrons directly into the conduction band, initiating photocatalytic reactions.^[^ ^51^ ^]^ Notably, modifications to TiO_2_ substantially improved both glucose conversion efficiency and gluconic acid selectivity.^[^ ^56^ ^]^ For instance, a mesoporous TiO_2_ photocatalyst synthesized via a rapid sol‐microwave method, assisted by the cationic surfactant cetyltrimethylammonium bromide (CTAB), achieved a 62.28% conversion for glucose.^[^ ^56^ ^]^ The reaction products included gluconic acid, xylitol, and trace amounts of arabinose and HCOOH. Compared to mesoporous TiO_2_ prepared using polyethylene glycol (PEG) as a surfactant, CTAB exhibited a greater effect on pore structure regulation, particularly in TiO_2_ synthesized with ultrasonic assistance. CTAB‐assisted TiO_2_ demonstrated a larger specific surface area and higher photocatalytic activity than its PEG‐assisted counterpart.^[^ ^57^ ^]^ In addition to controlling the specific surface area of TiO_2_, surfactants also modulate the crystalline phase composition (e.g., the anatase‐to‐rutile ratio) and particle dispersion, which were crucial for enhancing photocatalytic activity and product selectivity.^[^ ^56^, ^57^ ^]^ Another strategy for tuning the pore structure of TiO_2_ involved using zeolite Y (ZeY) as a support.^[^ ^58^ ^]^ By adjusting the SiO_2_/Al_2_O_3_ ratio in the zeolite, the pore structure of the catalyst could be optimized, thereby improving reactant adsorption and diffusion efficiency. Furthermore, metal loading (e.g., Ag or Cu) enhanced catalytic performance by reducing the bandgap and improving charge separation efficiency.^[^ ^58^ ^]^ Under optimized conditions, 1 wt.% Ag‐TiO_2_(40%)/ZeY achieved a glucose conversion of 96.9%, with the primary products being HCOOH, gluconic acid, arabinose, and xylitol.

To simplify the catalyst preparation process, an ultrasound‐assisted sol–gel method has been developed to synthesize TiO_2_ catalysts (TiO_2_(US)) with a high specific surface area, a well‐defined nanostructure, and improved visible light absorption.^[^ ^52^ ^]^ This method preserved the crystal phase of TiO_2_ while producing smaller crystallite sizes, specifically 12.7 nm. Additionally, ultrasonic treatment introduced O vacancies (Ovs) into the TiO_2_ structure, enhancing its visible light absorption and improving light utilization efficiency.^[^ ^52^ ^]^ The strategy of enhancing the specific surface area of catalysts to improve the photo‐oxidation rate of glucose has proven to be effective.

Acidification modification was an effective strategy for enhancing the catalytic oxidation of glucose by TiO_2_.^[^ ^59^ ^]^ Specifically, hetero‐polyacid (HPA)/TiO_2_ composites were synthesized by incorporating commercial Keggin‐type HPA H_3_PW_12_O_40_ (PW_12_) and a custom‐synthesized single‐vacancy K_7_PW_11_O_39_ salt (PW_11_) into TiO_2_ through wet impregnation and solvothermal methods. HPAs could absorb UV light, generating an excited state known as “heteropoly blue,” which exhibited strong oxidizing properties. This characteristic helped suppress charge recombination in TiO_2_, thereby enhancing photocatalytic performance.^[^ ^59^ ^]^ Notably, PW_12_ facilitated the desorption of products such as gluconic acid and glucaric acid, leading to improved selectivity.^[^ ^60^ ^]^ These results demonstrated that introducing acidic sites in catalytic materials facilitates reactant molecule adsorption. Simultaneously, the mutual repulsion between acidic sites promoted product desorption from the catalyst surface, thereby preventing product over‐oxidation.

Metal doping was a widely adopted strategy in photocatalysis, primarily due to its ability to extract photoelectrons or trap photogenerated h^+^.^[^ ^61^, ^62^, ^63^ ^]^ Notably, Ag‐doped TiO_2_, when calcined under an N_2_ atmosphere, exhibited adjustments in both specific surface area and particle size (≈150 nm).^[^ ^61^ ^]^ As the Ag content increased, the average diameter of the nanofibers gradually enlarged, contributing to enhanced photocatalytic activity. Furthermore, modification of TiO_2_ photocatalysts through metal/metalloid and non‐metal doping significantly improved their photocatalytic performance in glucose conversion.^[^ ^62^ ^]^ Co‐doping, particularly with Ag and N, enhanced the physicochemical properties of TiO_2_ through synergistic effects, resulting in higher glucose conversion and increased product yields. This improvement was primarily attributed to the increased specific surface area of co‐doped TiO_2_ (227.69 m^2^g^−1^) compared to pristine TiO_2_, which provided various active sites. Additionally, incorporating dopant elements into the TiO_2_ lattice induced lattice distortion, a key factor in boosting photocatalytic activity.^[^ ^62^ ^]^ Moreover, Ag nanoparticles (NPs) could absorb visible light and transfer the energy to the TiO_2_ surface. The resulting Schottky barrier at their junction suppressed rapid recombination, leading to enhanced charge carrier lifetime and improved separation efficiency under visible light.^[^ ^62^ ^]^

##### Others

**Figure 7:**
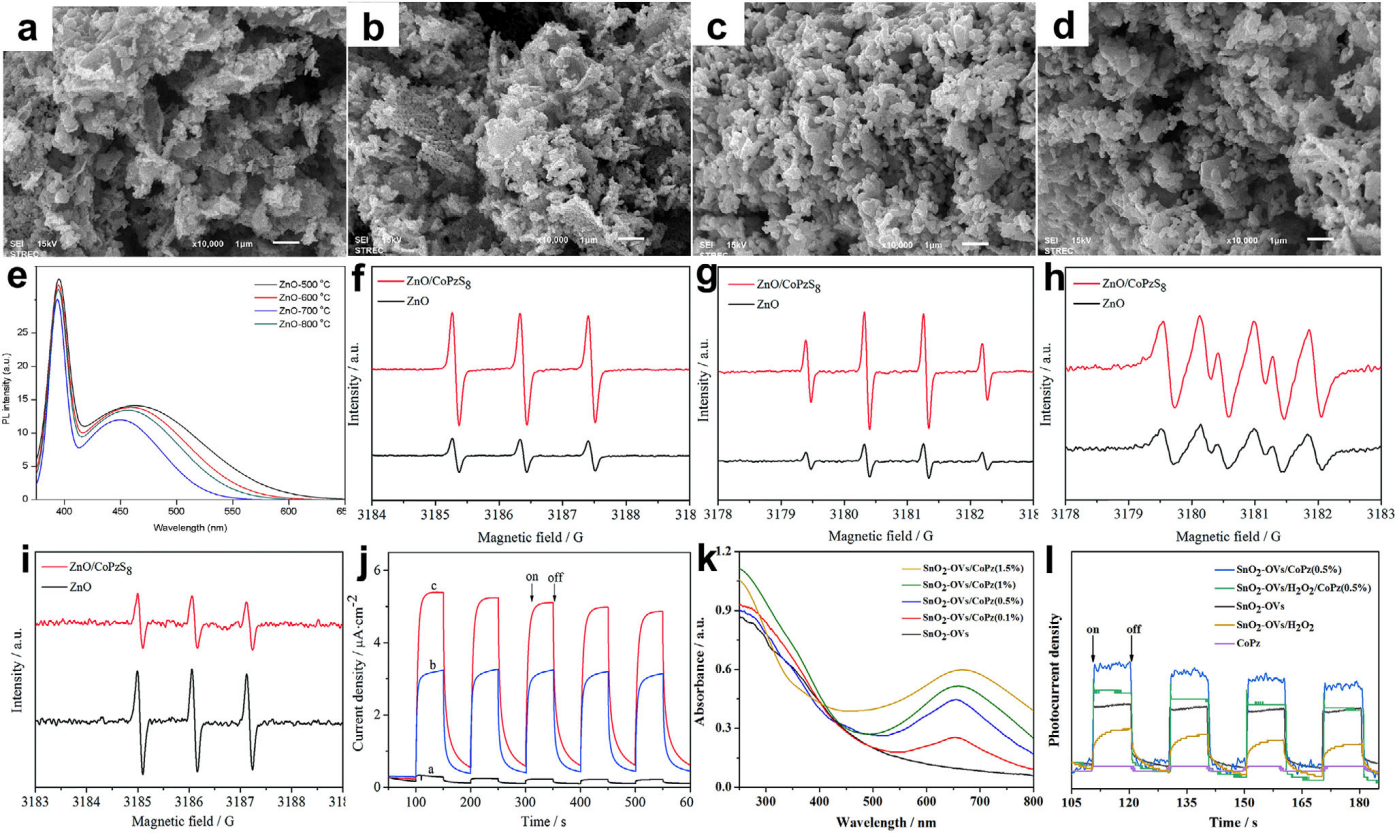
a–d) SEM images of ZnO‐500 °C, ZnO‐600 °C, ZnO‐700 °C, and ZnO‐800 °C. e) Photoluminescence (PL) spectra of ZnO NPs calcined at various temperatures. Reproduced with permission.^[^ ^25^ ^]^ Copyright 2023, American Chemical Society. EPR spectra of f) ^1^O_2_, g) •OH, h) •O_2_ˉ, and i) eˉ. j) Photocurrents of pure CoPzS8 a, pure ZnO b, and ZnO/CoPzS8 (0.5%) c. Reproduced with permission.^[^ ^64^ ^]^ Copyright 2019, Royal Society of Chemistry. k) UV–vis DRS of SnO_2_‐OVs and different SnO_2_‐OVs/CoPz composites. l) Photocurrents of CoPz, SnO_2_‐OVs/H_2_O_2_, SnO_2_‐OVs, SnO_2_‐OVs/H_2_O_2_/CoPz(0.5%), and SnO_2_‐OVs/CoPz(0.5%). Reproduced with permission.^[^ ^65^ ^]^ Copyright 2021, American Chemical Society.

ZnO NPs were synthesized using a polymer‐assisted co‐precipitation method with polyvinylpyrrolidone (PVP), followed by calcination at 700 °C, resulting in a catalyst with a high specific surface area and a high concentration of OVs.^[^ ^25^ ^]^ During the preparation process, the particle size, degree of agglomeration, and introduction of defects were controlled by adjusting the amount of PVP and the calcination temperature, leading to enhanced photocatalytic performance (**Figure** **7**a–e).^[^ ^25^ ^]^ As a result, the catalyst exhibited exceptional efficiency in the photocatalytic conversion of glucose, transforming glucose into high‐value‐added chemicals without additional acids or alkalis. This approach presented a promising strategy for applying ZnO NPs in green chemical synthesis. Beyond defect engineering, constructing composite photocatalysts was an alternative approach to boost the photocatalytic efficiency of ZnO.^[^ ^64^ ^]^ Notably, cobalt tetra(2,3‐bisbutylthio)phthalocyanine (CoPzS_8_), a metal phthalocyanine‐based photosensitizer, demonstrated strong visible light absorption and high photocatalytic activity. By immobilizing CoPzS_8_ on the surface of ZnO, a composite photocatalyst was developed, integrating the visible light absorption properties of CoPzS_8_ with the photocatalytic capabilities of ZnO.^[^ ^64^ ^]^ The interplay between these two components improved photogenerated charge carrier transfer and energy exchange, markedly boosting photocatalytic efficiency and increasing ROS production (Figure 7f–j). Consequently, this composite system achieved an efficient photocatalytic oxidation reaction.

SnO_2_, a wide‐bandgap semiconductor, possesses benefits such as affordability, non‐toxicity, and strong chemical stability. However, its photocatalytic performance is still restricted.^[^ ^65^, ^66^ ^]^ Introducing OVs could create electron‐trapping centers, effectively narrowing the bandgap and enhancing photocatalytic performance.^[^ ^65^ ^]^ A SnO_2_‐OVs/CoPz composite photocatalyst was successfully synthesized by loading cobalt phthalocyanine (CoPz) onto SnO_2_ with OVs. This design combined the robust visible light absorption of CoPz with the effective charge‐separation capabilities of SnO_2_‐OVs, creating a synergistic effect that greatly improved the efficiency (43.6%) and selectivity (81.5%) of photocatalytic glucose oxidation (Figure 7k–l).^[^ ^65^ ^]^ Additionally, FePz(SBu)_8_, a highly efficient metallophthalocyanine‐based photosensitizer, exhibited excellent visible light absorption capacity and photocatalytic performance.^[^ ^66^ ^]^ When FePz(SBu)_8_ was employed as an alternative to CoPz in constructing a SnO_2_/FePz(SBu)_8_ composite catalyst, a glucose conversion of 34.2% was achieved. The lower conversion compared to the SnO_2_‐OVs/CoPz system was likely due to the absence of OVs, which acted as eˉ traps in the former.^[^ ^66^ ^]^ An alternative strategy involved immobilizing FePz(SBu)_8_ onto H‐ZSM‐5 zeolite, taking advantage of the zeolite's structural robustness and large surface area. This configuration enabled the selective transformation of glucose into products such as gluconic acid, arabinose, glycerol, and formic acid under visible light, achieving a conversion efficiency of 35.8%.^[^ ^67^ ^]^ The resulting H‐ZSM‐5/FePz(SBu)_8_ hybrid demonstrated significantly superior photocatalytic performance compared to either component alone. This improvement was attributed to the introduction of H_2_O_2_ as an oxidant, which acted as a “reaction medium modulator,” promoting the generation of ROS and further enhancing catalytic performance.

Plasmonic nanocomposite photocatalysts, incorporating metal NPs (Ag or Au) and CdS semiconductor NPs embedded within a mordenite (MOR) matrix (M‐CdS/MOR, M = Ag, Au) were engineered for effective glucose‐to‐glucuronic acid conversion.^[^ ^68^ ^]^ In these composite catalysts, the localized surface plasmon resonance (LSPR) effect of Au, combined with the photocatalytic properties of CdS, broadened the spectral absorption range and served as an eˉ reservoir, thereby prolonging the lifetime of photogenerated charge carriers. Additionally, the MOR matrix provided high thermal stability, catalytic activity, and strong adsorption capacity. Embedding metal NPs and CdS within the zeolite matrix significantly enhanced the overall photocatalytic performance, achieving a 38% glucose conversion within 60 min.^[^ ^68^ ^]^ Notably, by utilizing H_2_O_2_ as an economical oxidant and eˉ scavenger, along with the LSPR effect of Au, the complete conversion of glucose to sodium gluconate was achieved in just 10 min under alkaline conditions.^[^ ^23^ ^]^ This method has also been successfully applied to various oligosaccharides, yielding products with over 95% purity and eliminating the need for additional purification steps. The exceptional photocatalytic efficiency was attributed to the extended separation of eˉ‐h^+^ pairs facilitated by the Schottky barrier at the Au/CeO_2_ interface, which significantly enhanced photocatalytic activity.^[^ ^23^ ^]^

**Figure 8:**
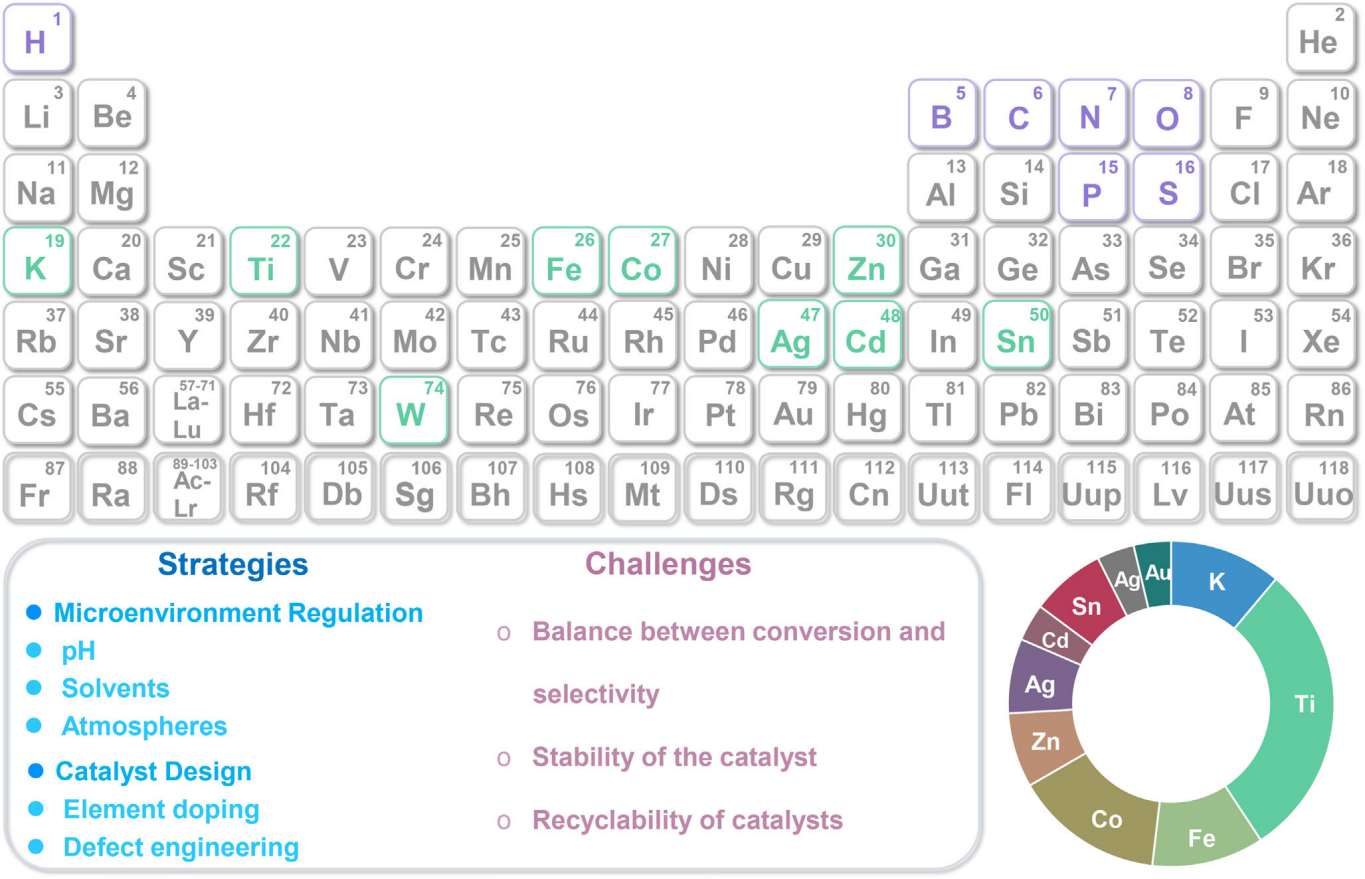
Current status and challenges in photocatalyst design for gluconic acid production from glucose.

***Summary***. Photocatalytic preparation of gluconic acids has made significant progress from glucose, particularly in catalyst design and the elucidation of reaction mechanisms (Table S1, Supporting Information). Through the development of novel photocatalysts (NiTiO_3_, Pt/TiO_2_, and CdS/TiO_2_/BC) and optimization of reaction conditions (pH, solvent composition, atmosphere), researchers have achieved high selectivity in producing high‐value‐added sugar acids (**Figure** **8**). These studies demonstrated that for visible‐light‐responsive catalysts, elemental doping serves as the primary modification strategy to enhance ROS generation capacity. For wide bandgap materials, additional defect engineering was required to modulate their band structures and optimize light response performance. Collectively, this body of work highlighted the inherent environmental advantages and green chemistry potential of photocatalytic processes. However, several challenges remain, including reduced selectivity at high conversion, insufficient catalyst stability and recyclability, and difficulties in scaling up due to the complexity of reaction conditions. Subsequent investigations ought to emphasize performance refinement of catalytic systems, operational condition simplification, and thorough practicality assessment to accelerate commercial deployment of this photocatalytic conversion technology.

### 3.2 HMF

The preparation of HMF by photocatalytic glucose conversion is an environmentally friendly, energy‐efficient, and high‐yield process. By utilizing solar energy as the driving force, this method eliminates the need for high temperatures and pressures, significantly reducing both energy consumption and environmental impact.^[^ ^69^, ^70^ ^]^ As a renewable biomass resource, glucose holds great economic potential for conversion, while the catalyst exhibits excellent recyclability and stability, further lowering operational costs.^[^ ^71^ ^]^ HMF, a key platform chemical, acts as a precursor for numerous high‐value products such as bio‐based plastics, fuels, fine chemicals, and pesticide intermediates.^[^ ^72^, ^73^ ^]^ Consequently, the photocatalytic production of HMF from glucose represents a promising and sustainable approach to green chemical synthesis.

#### 3.2.1 Mechanism

**Figure 9:**
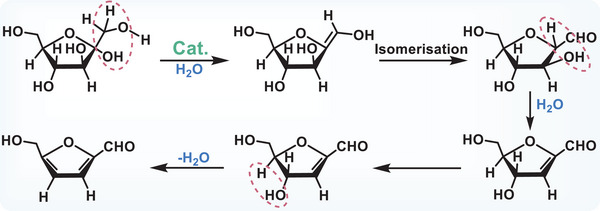
Schematic reaction mechanism of HMF produced by photocatalytic conversion of glucose. Reproduced with permission.^[^ ^33^ ^]^ Copyright 2024, American Chemical Society.

Solar‐driven glucose‐to‐HMF conversion proceeds via a triple dehydration pathway, where catalyst acidity serves as the key determinant of reaction efficiency.^[^ ^33^, ^74^ ^]^ In the initial step, the catalyst interacts with the ─OH and ─H groups at the C1 and C2 sites of glucose, promoting dehydration through water release and subsequently generating an enediol intermediate (**Figure** **9**).^[^ ^33^ ^]^ This intermediate then rapidly isomerizes into its aldehyde form. In the subsequent dehydration steps, additional water molecules are sequentially removed, ultimately leading to the formation of HMF.^[^ ^33^ ^]^

#### 3.2.2 Catalyst Design

**Figure 10:**
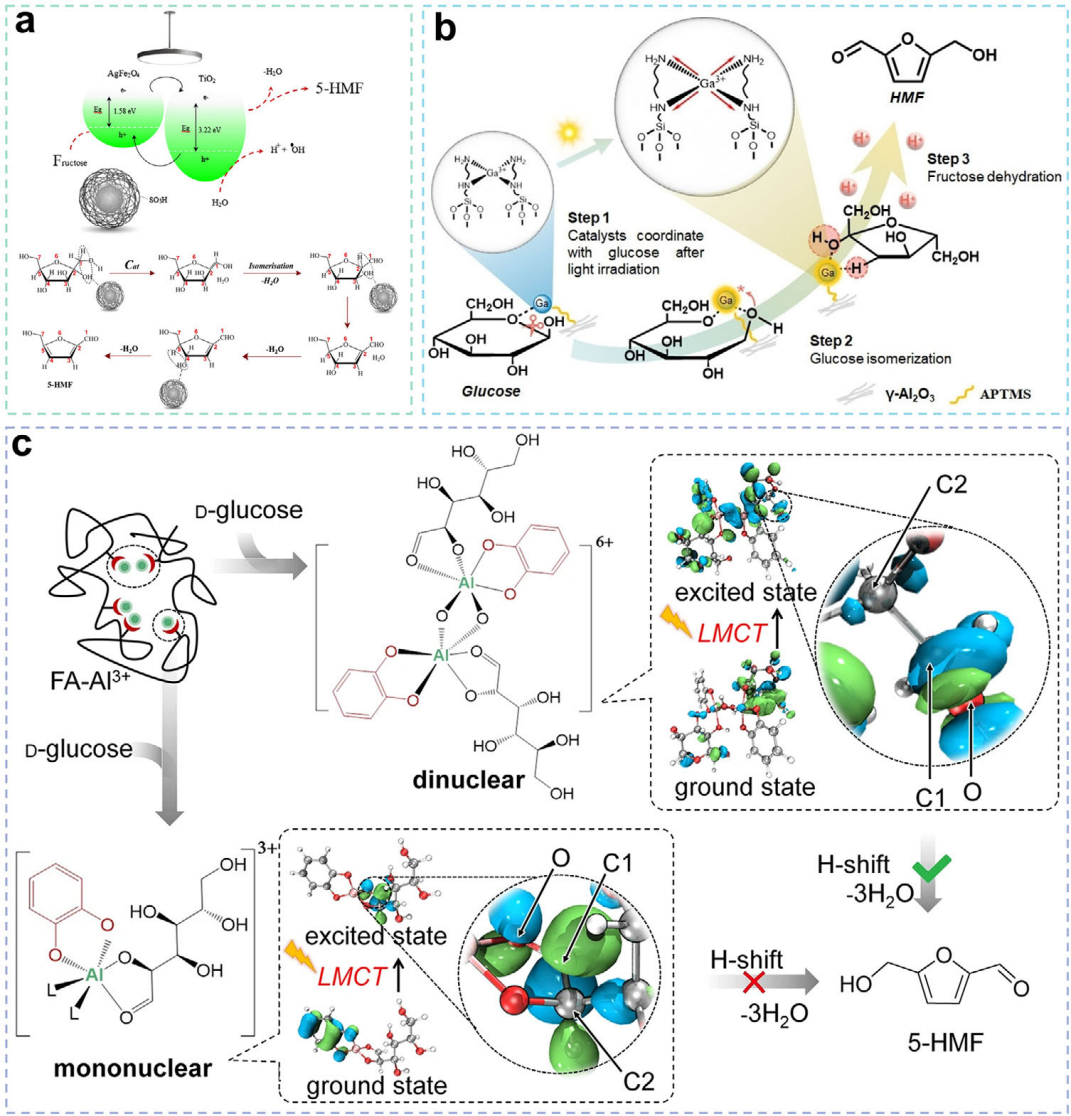
a) Mechanism of the conversion of glucose to HMF over AgFe_2_O_4_/TiO_2_‐SO_3_H. Reproduced with permission.^[^ ^33^ ^]^ Copyright 2024, American Chemical Society. b) Mechanism of glucose conversion to HMF over Ga(III) catalyst. Reproduced with permission.^[^ ^14^ ^]^ Copyright 2024, Wiley‐VCH GmbH c) Scheme of the glucose conversion to HMF with aluminum (III)‐catechol‐glucose complexes. Reproduced with permission.^[^ ^28^ ^]^ Copyright 2023, Springer Nature.

Fly ash and waste rice‐cooking water were utilized as raw materials to synthesize a nano‐photocatalyst (FA_NPC*) through a synergistic energy system combining tungsten halogen radiation and ultrasound (THUS).^[^ ^74^ ^]^ Fly ash was rich in amorphous silicoaluminates and iron oxides, while waste rice‐cooking water contained abundant starch, which could be converted into glucose and HMF. Simultaneously, Fe^3+^/Fe^2+^NPs were synthesized via the THUS system and combined with Al_2_SiO_5_ and Ti^4+^ to form a core‐shell structure. This structure prevented the photo‐dissolution of Fe^3+^/Fe^2+^, preserved the catalyst's magnetic properties, and enhanced its reusability.^[^ ^74^ ^]^ Acidic functional groups also played a crucial role in catalyzing glucose conversion to HMF. Pouya et al. developed a heterojunction photocatalyst using Al_2_SiO_5_ and TiO_2_ as precursors.^[^ ^33^ ^]^ AgFe_2_O_4_, a spinel ferrite, exhibited excellent visible light responsiveness, a high specific surface area, and a narrow bandgap. Further modification with sulfonic acid groups (─SO_3_H) resulted in the AgFe_2_O_4_/TiO_2_‐SO_3_H nanocomposite, which demonstrated significant light absorption across the visible spectrum.^[^ ^33^ ^]^ Due to its abundant active sites, this material enabled highly efficient sugar conversion under mild conditions, achieving an HMF yield as high as 98% (**Figure** **10**a). Notably, Ag NPs could enhance the photocatalytic activity of immobilized metal ions (such as Cr^3+^, Rh^3+^, Al^3+^, etc.) for sugar conversion to HMF under mild conditions.^[^ ^71^ ^]^ AgNPs improved light absorption via the LSPR effect. Meanwhile, metal ions (e.g., Cr^3+^) coordinate with sugar molecules to form intermediates, facilitating isomerization and dehydration reactions. Interestingly, the addition of a small amount of water lowered the activation energy of Cr^3+^, promoting sugar conversion to HMF, with a final yield reaching up to 68%.^[^ ^71^ ^]^ Collectively, these systems aimed to achieve efficient glucose‐to‐HMF conversion under mild conditions by employing various strategies to address challenges in selectivity, stability, and energy efficiency. The respective approaches emphasized: 1) sustainable material architectures, 2) precise engineering of acidic sites, and 3) innovative photo‐chemo synergistic mechanisms. Integrating these complementary strengths would offer significant potential for designing next‐generation high‐performance photocatalysts.

By immobilizing Ga(III) onto alumina nanofibers using silane with terminal amino groups (3‐2‐APTMS) as a ligand, researchers developed a stable heterogeneous catalyst.^[^ ^14^ ^]^ The coordination environment formed between Ga(III) and 3‐2‐APTMS significantly enhanced visible light absorption via the LMCT effect. Additionally, it increased the Lewis acidity of the catalyst, thereby promoting sugar transformation (Figure 10b). Mechanistic analysis revealed that Ga(III) functioned as a Lewis acid, facilitating the glucose‐fructose isomerization as the conversion's rate‐limiting step (RDS).^[^ ^14^ ^]^ The active Ga(III) sites interacted more strongly with the ─OH of sugar substrates, accelerating C─O bond scission and dehydration processes to achieve 57% HMF yield.

The coordination of Al^3+^ with polyphenolic compounds in fulvic acid, such as catechol and pyrogallol, formed complexes capable of absorbing visible light in the 350–600 nm range.^[^ ^28^ ^]^ Upon photoexcitation, electron density redistribution within the Al^3+^‐polyphenol complexes facilitated the isomerization of glucose to fructose, followed by fructose dehydration to produce HMF (Figure 10c). In this process, the RDS was the 1,2‐hydrogen migration or C2 proton extraction under photoexcitation.^[^ ^28^ ^]^ To improve both the acidity and light‐driven catalytic performance of carbon nitride‐based materials, ─SO_3_H and ionic liquids (ILs) were grafted onto g‐C_3_N_4_.^[^ ^75^ ^]^ Additionally, a novel acidic photocatalyst, TiO_2_/g‐C_3_N_4_/SO_3_H(IL), was developed by integrating TiO_2_, g‐C_3_N_4_, and sulfonic acid‐functionalized ILs, leveraging the synergistic effects of these components.^[^ ^75^ ^]^ Under visible light irradiation, this catalyst exhibited high efficiency in converting glucose to HMF in an aqueous medium, achieving an optimal yield of 92%. This demonstrated how metal‐ligand coordination engineering innovatively resolved critical bottlenecks in photocatalytic biomass conversion through: 1) elucidating Lewis acid site regulation of RDS, and 2) designing triple‐synergy catalysts integrating acidic sites, photoactivity, and mass transfer. Collectively, this established a new paradigm for visible‐light‐driven, aqueous‐phase‐compatible, and highly selective biomass conversion systems via synergistic control of Lewis acidity, photogenerated carrier dynamics, and mass transport.

**Figure 11:**
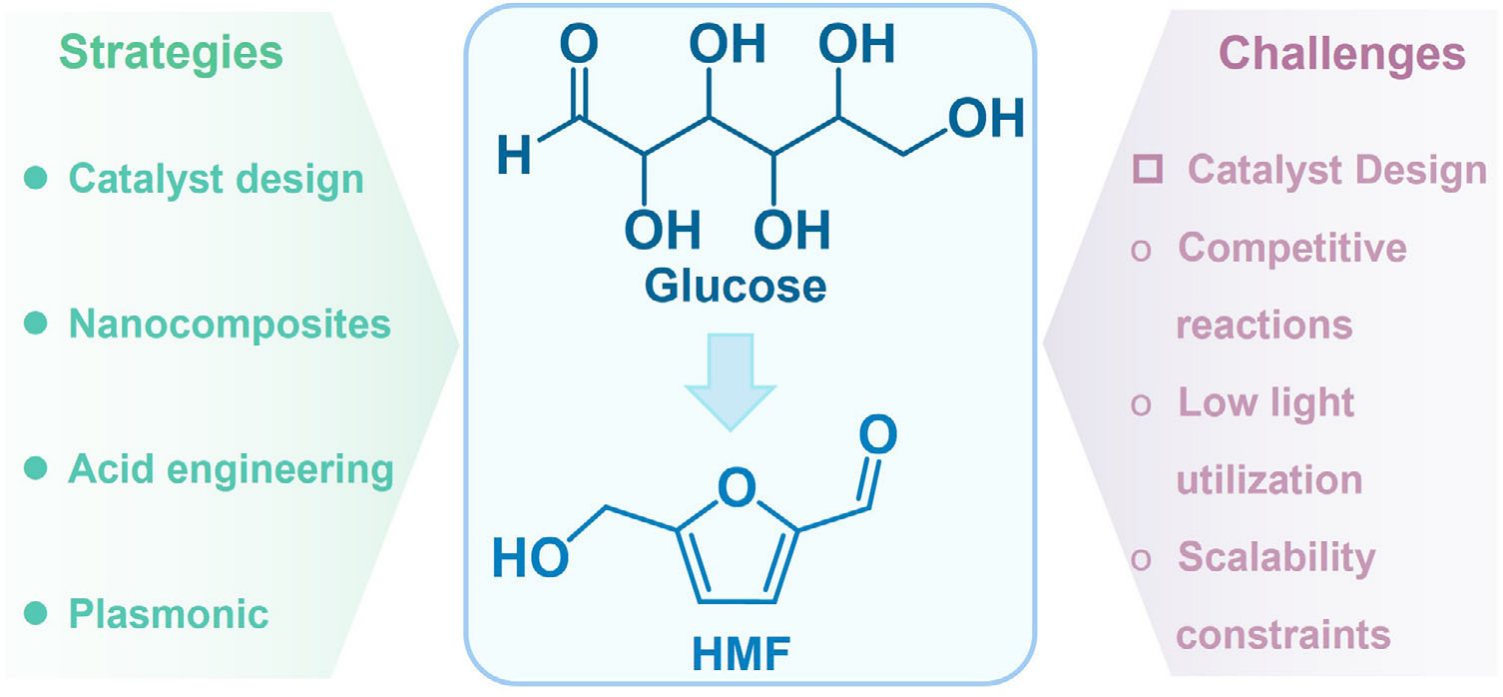
Strategies and challenges in designing photocatalysts for HMF production from glucose.

***Summary***. Photocatalytic technology has enabled significant advances in glucose‐to‐HMF conversion through rationally designed catalysts, including waste‐derived nanocomposites (e.g., fly ash/rice water coreshells preventing metal leaching), plasmonic‐ionic hybrids (AgNPs/Cr^3+^ leveraging LSPR effects), and acid‐engineered heterostructures (Ga(III)/Al_2_O_3_ nanofibers optimizing Lewis acidity via LMCT) (**Figure** **11**; Table S2, Supporting Information). These systems have achieved over 90% HMF selectivity under mild conditions (< 100 °C) by targeting C1/C2 hydroxyl activation and simultaneously suppressing fragmentation pathways. Despite improved sustainability through renewable feedstocks and energy‐efficient operation, persistent challenges involve competing reactions, limited visible‐light utilization beyond 600 nm, and scalability constraints. Consequently, next‐generation designs must integrate precision acid‐base bifunctionality, full‐spectrum photon management, and modular architectures to enable continuous‐flow operation, which is critical for industrial‐scale implementation of solar‐driven biomass valorization.

### 3.3 Arabinose

Photocatalysis enables the efficient conversion of glucose into arabinose through the selective cleavage of the C1─C2 bond, offering a significant advantage over traditional methods, which are often costly and involve complex procedures.^[^ ^22^ ^]^ Arabinose, a natural low‐calorie sweetener, provides several health benefits, including inhibiting sucrose absorption, regulating blood glucose levels, and reducing fat accumulation.^[^ ^76^ ^]^ This compound finds extensive application across food, nutraceutical, and pharmaceutical sectors for preventing and controlling metabolic conditions, including obesity, high blood pressure, and elevated lipid levels.^[^ ^77^ ^]^ With the growing demand for healthier dietary options, the photocatalytic production of arabinose represents a sustainable manufacturing approach and meets the increasing market demand for functional sweeteners, highlighting its substantial potential for broad application.^[^ ^78^ ^]^

#### 3.3.1 Mechanism

**Figure 12:**
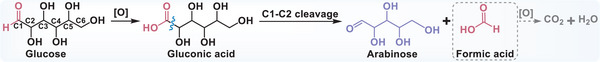
Reaction mechanism of photocatalytic preparation of arabinose and HCOOH from glucose.

Photocatalytic preparation of arabinose and HCOOH via photocatalytic transformation of glucose proceeds through an identical *α*‐cleavage pathway at the C1─C2 bond (**Figure** **12**).^[^ ^77^ ^]^ In this process, arabinose formation is accompanied by the generation of HCOOH as a byproduct. However, unlike arabinose, formic acid can undergo further oxidation, resulting in the production of CO_2_ and H_2_O.^[^ ^79^ ^]^ Hence, the rational design of catalysts capable of selectively breaking C─C linkages in glucose, while suppressing excessive oxidation, is of great importance. During the photocatalytic reaction, reactive species first interact with the C1 ─CHO of glucose, forming gluconic acid as an intermediate.^[^ ^80^ ^]^ Subsequent C1─C2 *α*‐cleavage and decarboxylation of gluconic acid lead to the production of HCOOH and arabinose.^[^ ^81^, ^82^ ^]^ Importantly, in this process, protons from H_2_O can also participate in reduction reactions, generating H_2_ as a valuable byproduct.

#### 3.3.2 Catalyst Design

By incorporating NaNO_2_ and NH_4_I into melamine during calcination yielded cyano‐functionalized defective carbon nitride (NICN).^[^ ^83^ ^]^ NICN exhibited significantly enhanced light absorption in both the ultraviolet (UV) and visible regions, with its bandgap narrowing from 2.64 eV for pristine g‐C_3_N_4_ to 2.40 eV, thereby improving its photocatalytic performance. Furthermore, the introduced ─CN groups, defects, and iodine atoms worked synergistically to extend carrier lifetime, accelerate charge transfer, and enhance both photocurrent density and light harvesting capacity, leading to superior photocatalytic performance.^[^ ^83^ ^]^ Consequently, NICN demonstrated a high glucose conversion (91.04% within 1 h) and exceptional selectivity toward arabinose (yield of 0.262 g/L).^[^ ^83^ ^]^ These findings demonstrated that incorporating ─CN groups, iodine atoms, and defects synergistically modulated the material's electronic structure. This multi‐faceted modification increased reactive site density, consequently enhancing charge carrier separation and mobility.

NiTiO_3_ emerged as a promising photocatalytic material owing to its appropriate bandgap for visible‐light absorption, excellent structural stability, and economic viability.^[^ ^22^ ^]^ Highly crystalline 1D NiTiO_3_ nanorods with controlled morphology were successfully fabricated through a sol–gel protocol coupled with thermal calcination. This engineered architecture promoted effective spatial charge separation and directional transport of photoinduced charge carriers, while its broad spectral absorption spanning UV to visible regions demonstrated a 2.3‐fold enhancement in visible light utilization compared to conventional TiO_2_.^[^ ^22^ ^]^ Interestingly, during the photocatalytic conversion of glucose driven by NiTiO_3_, precise control over product selectivity could be achieved by modulating the solvent pH, reaction time, solvent composition, and reaction atmosphere. Specifically, 1) under neutral conditions, the primary product of glucose photo‐reforming was arabinose, with a selectivity of up to 75%. 2) Under alkaline conditions, the main products were organic acids (lactic acid, acetic acid, and HCOOH), with a combined selectivity of 63%.^[^ ^22^ ^]^ Furthermore, increasing the proportion of acetonitrile enhanced the glucose conversion but reduced the selectivity toward arabinose. Adjusting the reaction atmosphere to include oxygen was crucial for the photo‐oxidation of glucose. However, high oxygen concentrations could inhibit the selective formation of arabinose.^[^ ^22^ ^]^

High‐purity anatase TiO_2_ (TiO_2_‐A) and rutile TiO_2_ (TiO_2_‐R) phases were synthesized via a hydrothermal method to investigate the impact of different TiO_2_ crystalline phases on the photocatalytic conversion of glucose to arabinose.^[^ ^84^ ^]^ TiO_2_‐A exhibited lower overall selectivity but demonstrated a higher ability to cleave C─C bonds, as indicated by a CO_2_/H_2_ molar ratio close to the theoretical value of 0.5. In contrast, TiO_2_‐R showed the highest overall selectivity (91%) and the lowest CO_2_/H_2_ ratio (0.017), suggesting its weaker ability for C─C bond cleavage and greater suitability for selective conversion.^[^ ^84^ ^]^ Electron paramagnetic resonance (EPR) experiments revealed that TiO_2_‐R primarily generated peroxide species under light irradiation, while TiO_2_‐A produced •OH. These findings offered valuable insights for designing catalysts tailored to specific photocatalytic reactions.^[^ ^84^ ^]^

**Figure 13:**
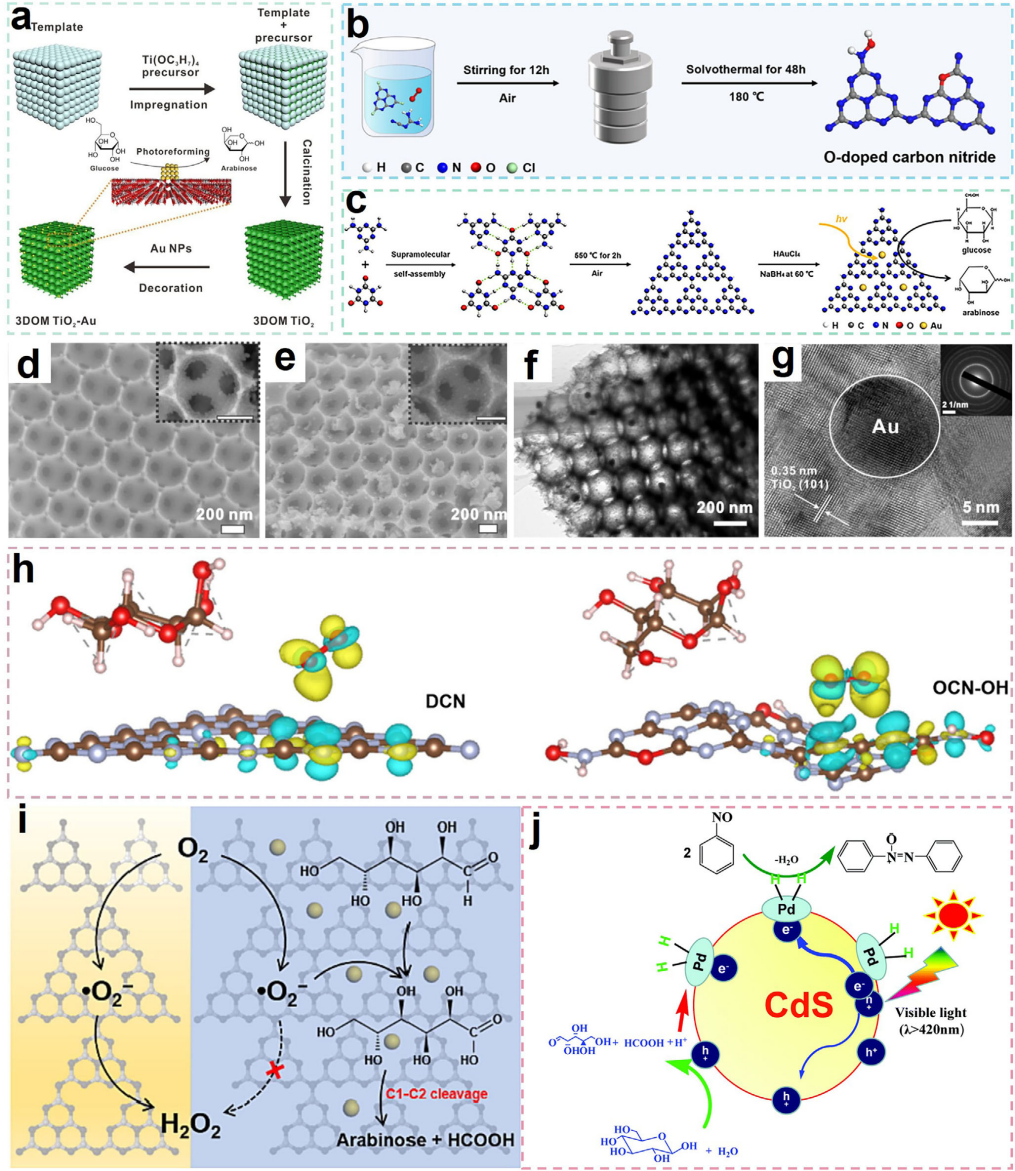
a) Schematic illustration of the fabrication of 3DOM TiO_2_‐Au. Reproduced with permission.^[^ ^79^ ^]^ Copyright 2021, Elsevier. b) Synthetic process of O‐doped CN. Reproduced with permission.^[^ ^24^ ^]^ Copyright 2022, American Chemical Society. c) Synthetic process of AuCN. SEM images of d) 3DOM TiO_2_ and e) 3DOM TiO_2_‐Au. f) TEM and g) HR‐TEM images of 3DOM TiO_2_‐Au. Reproduced with permission.^[^ ^79^ ^]^ Copyright 2021, Elsevier. h) Charge difference profiles of glucose and O_2_ adsorption on DCN and OCN‐OH. Reproduced with permission.^[^ ^24^ ^]^ Copyright 2022, American Chemical Society. i) Schematic illustration of photocatalytic glucose transformation into arabinose on AuCN. Reproduced with permission.^[^ ^78^ ^]^ Copyright 2022, Elsevier. j) Mechanism of arabinose preparation by photocatalytic conversion of glucose. Reproduced with permission.^[^ ^85^ ^]^ Copyright 2016, Royal Society of Chemistry.

3D ordered microporous (3DOM) TiO_2_ was synthesized via a polystyrene colloidal crystal templating approach, yielding a photocatalyst with exceptional specific surface area and broadband light‐harvesting capabilities (**Figure** **13**a).^[^ ^79^ ^]^ Structural characterization revealed a periodically arranged, interconnected porous network (pore diameter ∼380 nm) constructed from monodisperse, smooth‐surfaced TiO_2_ nanocrystallites (Figure 13d,e). This hierarchical architecture provided abundant anchoring sites for Au NPs, enabling their homogeneous dispersion within the macroporous framework while suppressing particle agglomeration (Figure 13f–g).^[^ ^79^ ^]^ Interestingly, the layered porous architecture of the 3DOM structure enhanced Au NPs the distribution and dispersion of Au NPs while simultaneously decreasing particle dimensions. The high specific surface area of the catalyst enhanced the interaction between glucose and the photocatalyst, promoting mass diffusion and improving conversion efficiency. Leveraging the LSPR effect of AuNPs enabled 37% glucose conversion with 0.2 g L^−1^ arabinose production.^[^ ^79^ ^]^

A hierarchical photocatalytic system was constructed through the integration of mesoporous CdS as a structural scaffold with PdNPs, achieving dual functionality in redox catalysis.^[^ ^85^ ^]^ The CdS matrix facilitated directional eˉ transport while creating active reduction centers through PdNPs incorporation. Based on this design, the catalyst achieved dual functionality: selective glucose‐to‐arabinose transformation coupled with nitrosobenzene‐to‐azoxybenzene conversion (Figure 13j). The selectivity for arabinose reached 70%, while that for azoxybenzene reached 92%. The catalyst design effectively utilized the advantages of the mesoporous structure and the enhancing effect of Pd NPs, enabling effective charge carrier separation and excellent.^[^ ^85^ ^]^ This approach offered a green and efficient photocatalytic method for biomass conversion and organic synthesis.

Through the rational design of O‐doped polymeric carbon nitride (Figure 13b), the research team led by Wang accomplished neutral pH‐driven photocatalytic transformation of cellulose into arabinose.^[^ ^77^ ^]^ This process utilized oxidative ROS (such as •O_2_ˉ and •OH) to facilitate selective oxidation reactions. Notably, using glucose as the reactant, OCN−OH achieved a glucose conversion of ≈10%, with arabinose selectivity exceeding 90% after 6 h of photocatalysis. Under an oxygen atmosphere, the glucose conversion increased to ≈28%, while the arabinose selectivity decreased but remained above 80%.^[^ ^77^ ^]^ Characterization of the material revealed that OCN−OH exhibited a stronger EPR signal, indicating that O‐doping introduced additional spin defects, which promoted effective charge carrier separation. DFT calculations showed that O‐doping altered the distribution of the highest occupied molecular orbital (HOMO) and lowest unoccupied molecular orbital (LUMO) in CN, enhancing the spatial separation of charge carriers.^[^ ^77^ ^]^ More importantly, the modified carbonitrides displayed a stronger affinity for oxygen (Figure 13h), which further facilitated the oxidation of glucose to arabinose.^[^ ^77^ ^]^

Polymeric carbon nitride (SCN) was prepared through a conventional supramolecular assembly process, utilizing cyanuric acid and melamine as starting materials and employing thermal polymerization. Subsequently, Au NPs were introduced into the SCN framework through NaBH_4_ reduction of HAuCl_4_, yielding the AuCN composite photocatalyst (Figure 13c). Decorating the surface of carbon nitride with Au NPs to regulate electron transfer behavior enabled a shift from the traditional two‐electron to single‐electron transfer mechanisms.^[^ ^78^ ^]^ This characteristic was confirmed by rotating disk electrode (RDE) measurements, which revealed that AuCN predominantly followed a single‐electron transfer pathway (n = 1.251) during oxygen reduction, while undoped CN mainly followed a two‐electron pathway (n = 1.878). This suggested that AuCN could selectively generate •O₂ˉ, thereby promoting glucose oxidation (Figure 13i).^[^ ^78^ ^]^ By adjusting the electron transfer pathway, AuCN achieved a glucose conversion of over 30%, with arabinose selectivity exceeding 40% and a yield of 12% after 6 h of photocatalysis. These findings elucidated the mechanisms of cellulose and glucose photocatalytic conversion, offering new insights for mild‐condition biomass valorization into valuable biochemicals.

**Figure 14:**
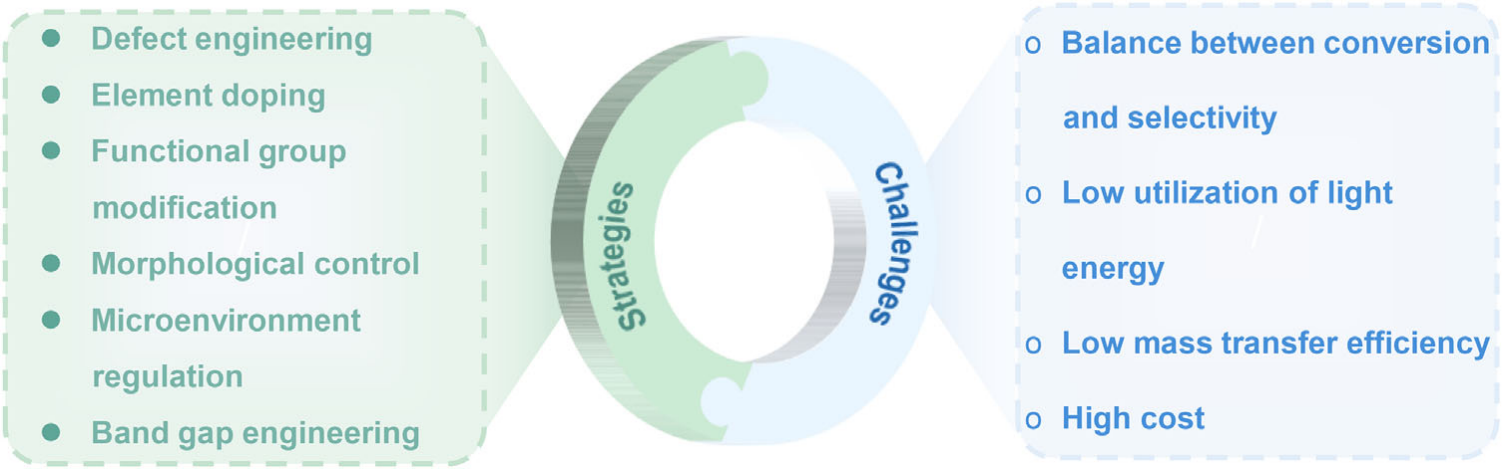
Design strategies and challenges for photocatalysts in arabinose production from glucose.

***Summary***. Researchers have developed numerous photocatalysts, including TiO_2_‐based materials and heterojunctions, which have demonstrated high selectivity for arabinose production under optimized conditions (**Figure** **14**; Table S3, Supporting Information). For instance, NiTiO_3_ achieved up to 75% arabinose selectivity under neutral conditions, while CdS/TiO_2_/BC demonstrated remarkable performance by tailoring the reaction microenvironment. These studies also highlighted the environmental friendliness of photocatalysis, leveraging water as both solvent and precursor for oxidants. From the catalyst modification processes discussed, it is evident that modifications of visible‐light‐responsive materials primarily aim to enhance charge carrier separation. For materials with narrow photoresponse ranges, adjusting the conduction band (CB) and valence band (VB) is essential to enable efficient oxygen activation. However, balancing selectivity and conversion remains a critical challenge, as high selectivity often comes at the cost of lower conversion efficiency.

### 3.4 Lactic Acid

Producing lactic acid by photocatalytic oxidation of glucose is a sustainable and highly efficient chemical process that is both environmentally friendly and cost‐effective. The high efficiency of photogenerated charge separation in photocatalysts ensures excellent reaction selectivity, enabling the rapid conversion of glucose to lactic acid with minimal by‐products.^[^ ^86^ ^]^ As an important organic acid, lactic acid is widely used in biotechnology, food production, pharmaceuticals, chemical engineering, and environmental protection, offering significant economic value.^[^ ^87^, ^88^ ^]^ For instance, lactic acid plays a key role in PLA synthesis, a compostable polymer widely employed in packaging, textiles, and medical applications.^[^ ^89^ ^]^ Within food processing, lactic acid and its derivatives serve as additives, preservatives, and flavor enhancers.^[^ ^90^ ^]^ The compound also demonstrates significant pharmaceutical potential due to its antimicrobial and anti‐inflammatory characteristics, enabling therapeutic applications.^[^ ^91^ ^]^ Therefore, photocatalytic glucose oxidation not only provides a sustainable chemical process but also opens up new opportunities for the broader application of lactic acid.

#### 3.4.1 Mechanism

**Figure 15:**
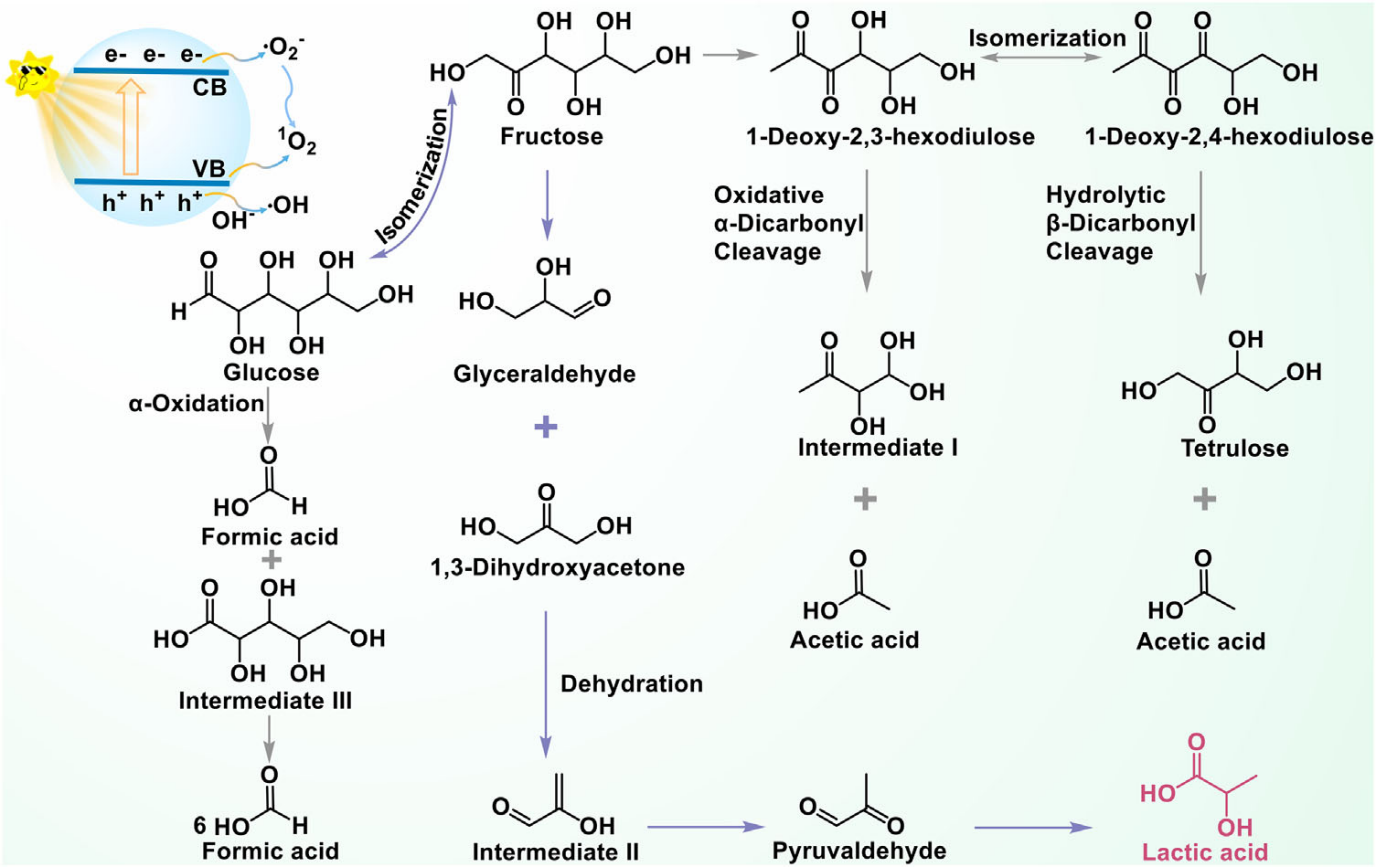
Schematic reaction mechanism of photocatalytic preparation of lactic acid from glucose. Reproduced with permission.^[^ ^92^ ^]^ Copyright 2022, Elsevier.

Upon photoexcitation, the eˉ in the CB of the catalyst interacts with molecular oxygen to generate •O_2_ˉ, while the h^+^ in the VB oxidizes H_2_O to produce •OH.^[^ ^92^ ^]^ The generated •O_2_ˉ can further react with h^+^ to form ^1^O_2_. These ROS facilitate the conversion of glucose to lactic acid. As shown in **Figure** **15**, during the photocatalytic process, glucose is first isomerized to fructose, which then transforms into 1‐deoxy‐2,3‐hexodiulose.^[^ ^92^, ^93^ ^]^ This intermediate can follow two pathways: (1) it is transformed into 1‐deoxy‐2,4‐hexodiulose, which undergoes hydrolytic *β*‐dicarbonyl cleavage to produce tetroses and acetic acid, or (2) it undergoes *α*‐dicarbonyl oxidative cleavage to form intermediate I and acetic acid.^[^ ^92^ ^]^ Meanwhile, fructose transforms glyceraldehyde and dihydroxyacetone, which then undergo dehydration to generate intermediate II. This intermediate is further processed to produce acetoin, finally leading to lactic acid formation.^[^ ^92^ ^]^ The intricate molecular architecture of glucose leads to multiple parallel reaction pathways during lactic acid production, generating various secondary products. Therefore, developing highly efficient and selective catalysts that promote the main reaction pathways while minimizing side reactions is of great significance.

#### 3.4.2 Catalyst Design

Complex product distribution due to photocatalytic preparation of lactic acid from glucose, the design of an effective photocatalyst necessitates precise modulation of its band structure, charge separation efficiency, surface active sites, and surface properties. Such careful control is crucial to achieving high selectivity and directing the oxidation pathway toward the formation of lactic acid.

##### g‐C_3_N_4_

**Figure 16:**
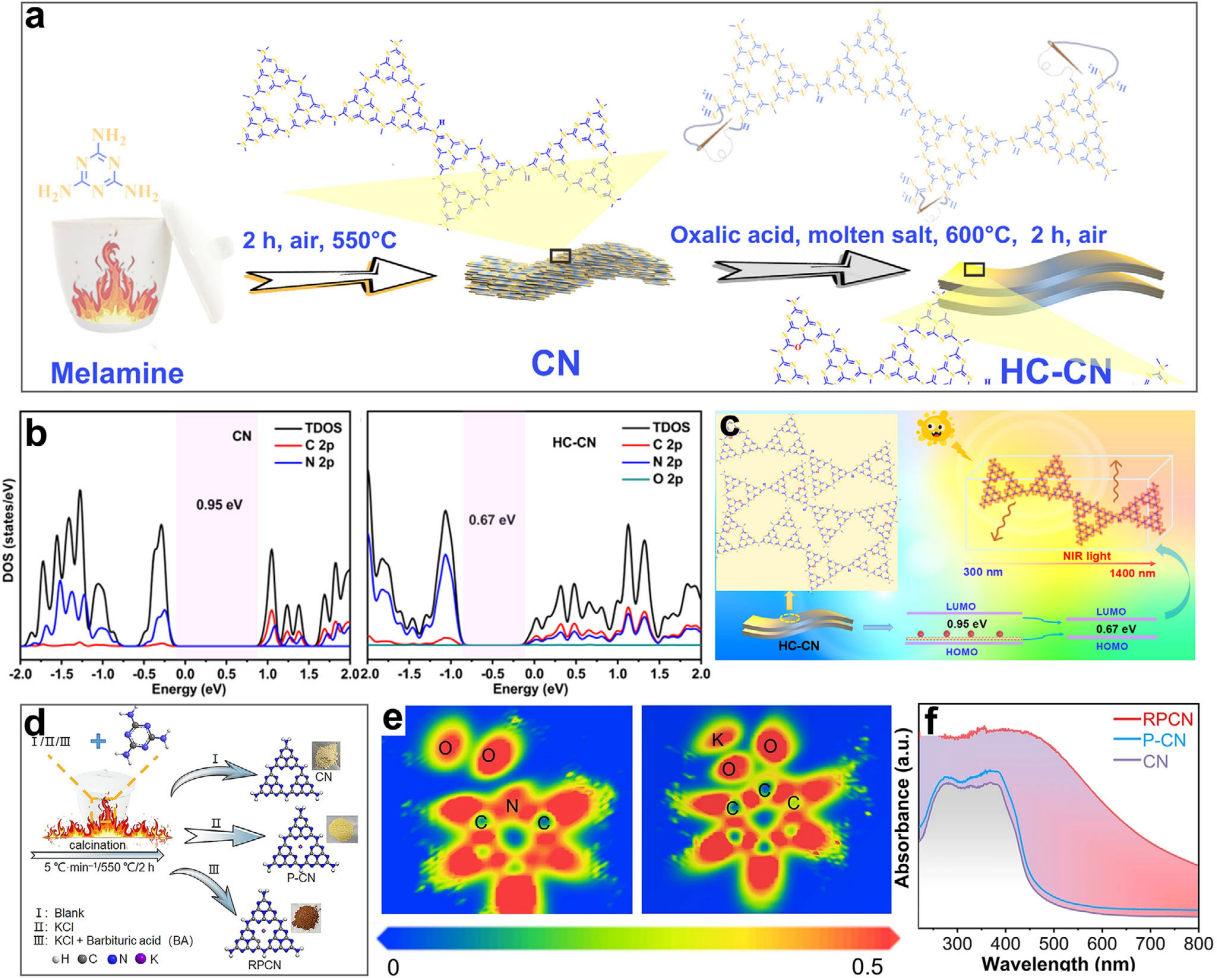
a) Schematic diagram of preparing HC‐CN. b) Density of states (DOS) of CN and HC‐CN. c) Schematic of HC‐CN structure‐light absorption performance relationship. Reproduced with permission.^[^ ^94^ ^]^ Copyright 2024, Wiley. d) Schematic illustration of preparing CN, P‐CN, and RPCN. e) Electron local function (ELF) of adsorbed O_2_ on CN and RPCN. f) UV–visible diffuse reflectance spectroscopy (UV–vis DRS) of CN, P‐CN, and RPCN. Reproduced with permission.^[^ ^27^ ^]^ Copyright 2025, Springer.

Non‐metal doping was an effective strategy for modifying g‐C_3_N_4_ materials to enhance their photocatalytic conversion of glucose into lactic acid. Studies have shown that co‐doping with boron (B) and O reduced the catalyst's bandgap from 2.64 to 2.31 eV as the doping levels increased.^[^ ^86^ ^]^ This reduction was attributed to the similar electronic orbital configurations of B, O, C, and N, which allowed the doping to significantly alter the catalyst's electronic structure. This modification greatly improved the catalyst's visible light absorption capacity and enhanced the separation efficiency of photogenerated charge carriers.^[^ ^86^ ^]^ This approach attained exceptional lactic acid production (yielding up to 92.7%), with the catalytic activity remaining above 99% even after 11 cycles of reuse. Additionally, a molten‐salt‐assisted thermal polymerization method was employed to introduce structural oxygen into the carbon nitride, replacing some N atoms (**Figure** **16**a).^[^ ^94^ ^]^ Structural oxygen doping altered the catalyst's electronic configuration and intensified localized electric field generation, resulting in significant bandgap reduction (Figure 16b). This study demonstrated that the molten‐salt‐assisted strategy, involving “plane stitching” and “interlayer cutting,” improved the catalyst's crystallinity and strengthened the π–π stacking effect.^[^ ^94^ ^]^ High crystallinity enhanced light absorption, expanding the absorption spectrum to near‐infrared wavelengths (≤1400 nm) and promoting the photothermal effect (Figure 16c). The combination of structural oxygen and the π–π stacking effect facilitated the generation of •O_2_ˉ, enabling selective C3─C4 bond cleavage and thus improving the selectivity for lactic acid production.^[^ ^94^ ^]^

The molten salt templating method was also effective for doping K into carbon nitride, enabling the construction of a catalyst with well‐distributed K/C dual active centers (Figure 16d).^[^ ^27^ ^]^ In this system, K sites functioned as Lewis acid sites to catalyze glucose‐to‐fructose isomerization, while N‐substituted C sites trapped eˉ to selectively produce •O_2_ˉ to cleave the C3─C4 bond of fructose. The synergy between K and C sites enhanced light absorption, modulated charge distribution, and promoted the selective generation of ROS during photocatalysis (Figure 16e–f), attaining 98.5% glucose conversion and 93.4% lactic acid yield in 1 h at 60 °C.^[^ ^27^ ^]^ The molten‐salt‐assisted method was also applicable to Mg‐modified carbon nitride. Combined with converter slag (CS) as a support material, it improved the dispersion of carbon nitride and mitigated agglomeration.^[^ ^95^ ^]^ The composite structure of Mg‐modified carbon nitride (Mg‐CN) and CS significantly enhanced photocatalytic performance. This improvement was primarily attributed to: i) Mg incorporation optimized carbon nitride's band structure, improved visible photon utilization, and suppressed charge recombination; and ii) the inherent basicity of metal oxides (such as CaO and Fe_2_O_3_) in CS, which promoted the isomerization of glucose to fructose, thereby increasing lactic acid selectivity.^[^ ^95^ ^]^ This dual‐modification strategy achieved 97% glucose conversion with 71% lactic acid production. Furthermore, replacing Mg‐CN with O‐doped g‐C_3_N_4_ (OCN) and compositing OCN with CS via thermal polymerization allowed for the uniform growth of OCN on the CS surface, forming an OCN/CS type‐II heterojunction. This further optimized the catalytic system, increasing the lactic acid yield to 90%.^[^ ^96^ ^]^

Compared with binary heterojunction catalysts, the ternary heterojunction photocatalyst g‐C_3_N_4_/N‐TiO_2_/NiFe‐layered double hydroxide (NTCN/LDH), synthesized via a facile wet‐chemical approach, exhibited superior photocatalytic activity, producing 92% lactic acid in 1 h at 60 °C.^[^ ^97^ ^]^ The enhanced performance could be attributed to two main factors. First, NiFe‐LDH provided Lewis acid sites that facilitated the thermal catalytic isomerization of glucose to fructose. Second, the constructed double type‐II heterojunctions regulated the formation of ROS, enabling precise photocatalytic cleavage of the C3─C4 bond.^[^ ^97^ ^]^ Furthermore, DFT calculations revealed that NTCN/LDH exhibited different adsorption affinities for glucose and fructose. This selective adsorption promoted the isomerization of sugars and enhanced the efficiency of subsequent photocatalytic C─C bond cleavage.

The uniform dispersion of Zn atoms within carbon nitride resulted in forming Zn SA sites, which served as active centers.^[^ ^98^ ^]^ This modification narrowed the bandgap of carbon nitride to 1.8 eV, thereby extending its light absorption range. Additionally, the establishment of Zn‐N6 coordination bonds in the carbon nitride framework substantially reduced interfacial charge transfer resistance, facilitating the efficient migration of photogenerated charge carriers.^[^ ^98^ ^]^ As a result, this structural optimization enabled the catalyst's ability to convert glucose into lactic acid while simultaneously co‐producing H_2_.

**Figure 17:**
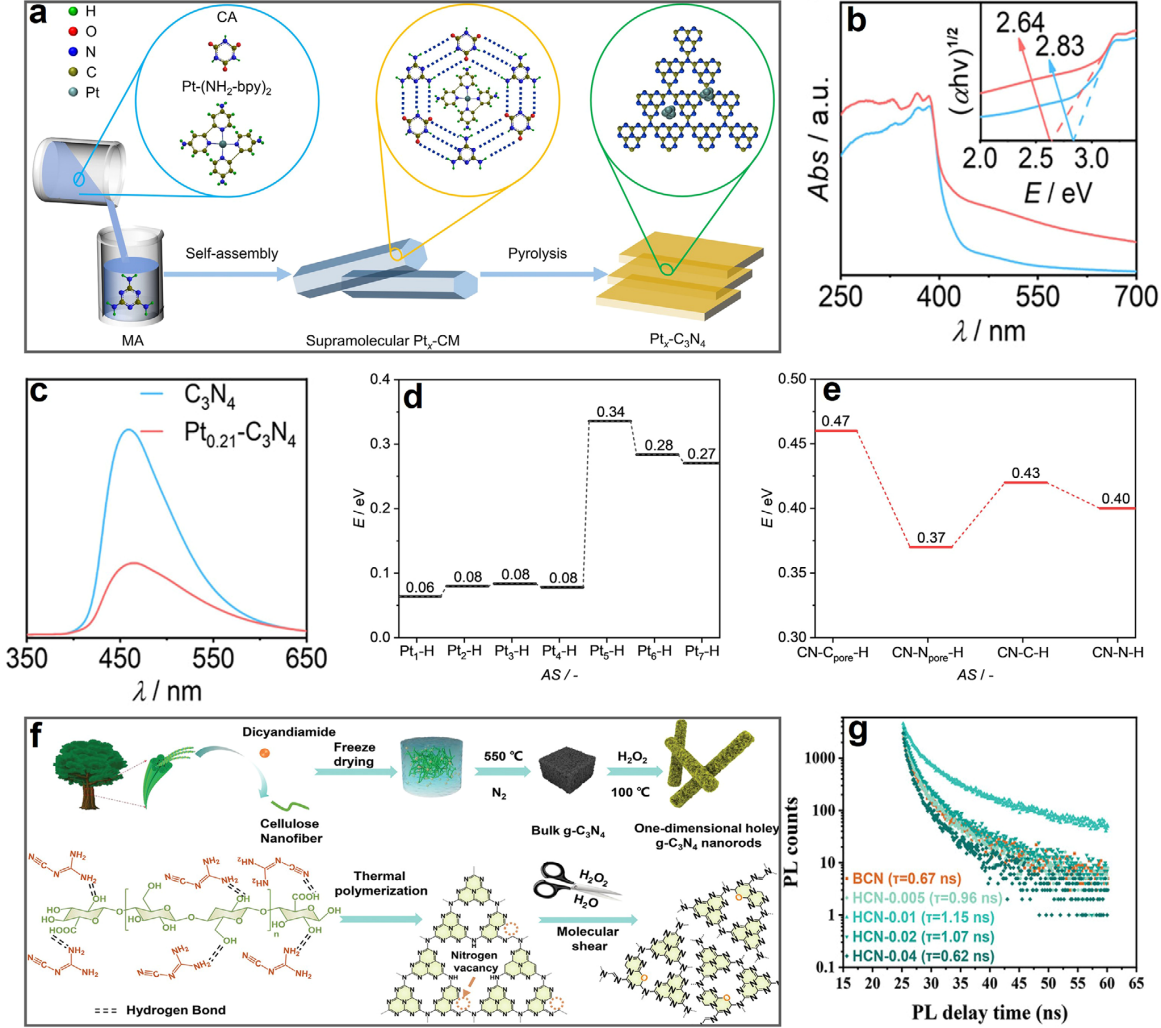
a) Schematic illustration of preparing Pt_x_‐C_3_N_4_. b) UV–vis DRS and c) PL spectra of C_3_N_4_ and Pt_0.21_‐C_3_N_4_. Gibbs free energy of H_2_O reduction to H_2_ on d) C_3_N_4_ and e) Pt_0.21_‐C_3_N_4_. Reproduced with permission.^[^ ^24^ ^]^ Copyright 2022, American Chemical Society. f) Schematic representation of the synthesis approach and chemical structure of the NVs HCN with O‐doping. g) TRPL spectra of BCN and HCN‐x. Reproduced with permission.^[^ ^93^ ^]^ Copyright 2023, Wiley‐VCH GmbH.

Pt NPs were incorporated into a preorganized supramolecular assembly of cyanuric acid, melamine, and Pt‐(NH_2_‐bpy)₂ to fabricate a polymeric carbon nitride‐based photocatalyst (**Figure** **17**a).^[^ ^24^ ^]^ The resulting Pt‐C_3_N_4_ catalyst, prepared via pyrolysis, formed curly nanosheets with numerous surface pores, ensuring high Pt NPs loading efficiency and uniform dispersion. Notably, depositing Pt NPs on C_3_N_4_ surface significantly improved the separation efficiency of photogenerated charge carriers (Figure 17c).^[^ ^24^ ^]^ In this composite, Pt NPs induced a red shift in the absorption edge via the surface plasmon effect, thereby enhancing visible light absorption and reducing the carbon nitride bandgap from 2.83 to 1.97 eV (Figure 17b). Additionally, the activation energy barrier for water reduction to H_2_ was significantly lowered (Figure 17d,e), while the selective cleavage of the C─C bond in fructose (formed via glucose isomerization) was promoted. As a result, lactic acid production efficiency increased more than ninefold compared to pristine C_3_N_4_. Moreover, the photocatalytic performance of Pt_0.21_‐C_3_N_4_ remained stable after four experimental cycles, demonstrating excellent reusability.^[^ ^24^ ^]^

Hydrothermal etching has emerged as an effective approach for co‐introducing N (NVs/OVs) into g‐C_3_N_4_ during defect engineering modifications (Figure 17f).^[^ ^93^ ^]^ By further integrating this approach with cellulose nanofiber (CNF)‐assisted polymerization, the morphology of g‐C_3_N_4_ was effectively regulated, transitioning from a 2D layered structure to 1D nanorod configurations. This transformation significantly enhanced the material's specific surface area and mass transfer efficiency. Leveraging the synergistic effects of NVs and OVs, the catalyst exhibited extended photogenerated charge carrier lifetimes and improved charge separation efficiency (Figure 17g).^[^ ^93^ ^]^ Additionally, the one‐dimensional porous nanorod structure facilitated both reactant uptake and product release.^[^ ^93^ ^]^ As a result, the catalyst achieved a lactic acid yield of 75.5% in glucose conversion. Notably, it also demonstrated a high H_2_ evolution activity (2.8 mmol g^−1^ h^−1^).

Regulating catalyst morphology was primarily aimed at enhancing surface area and active site accessibility.^[^ ^92^ ^]^ Following this principle, carbon nitride nanosheets were encapsulated within a 3D ordered porous polyvinyl alcohol (PVA) matrix using a freeze‐induced method, resulting in a “gill‐like” ordered porous structure.^[^ ^92^ ^]^ This design significantly increased the specific surface area to 9.2496 m^2^ g^−1^, compared with 4.4894 m^2^ g^−1^ for the pure PVA hydrogel, thereby enhancing its adsorption capacity.

Strategies for modifying g‐C_3_N_4_ to improve photocatalytic glucose conversion to lactic acid focus on three key aspects: 1) enhancing light absorption, 2) promoting charge carrier separation, and 3) enabling selective bond cleavage. For instance, non‐metal doping effectively narrowed the bandgap, significantly improving visible‐light utilization and charge separation. Molten‐salt‐assisted O doping altered electronic configurations, enhanced crystallinity, and improved π–π stacking, thereby extending light absorption into the near‐infrared region to enable photothermal synergy and selective •O_2_ˉ mediated C3─C4 bond cleavage. Metal doping introduced Lewis acid sites for glucose isomerization, while dual active centers or support composites optimized band structures and basicity. Heterojunction engineering regulated ROS formation via double type‐II junctions and Lewis acid‐catalyzed isomerization. Zn or Pt NP loading narrowed bandgaps to 1.8–1.97 eV via coordination bonds or surface plasmon effects, promoting charge migration and C─C bond cleavage with lactic acid yields up to ninefold higher than pristine C_3_N_4_. Defect engineering and 1D/3D morphology regulation enhanced specific surface area, mass transfer, and carrier lifetimes. Collectively, these strategies synergized electronic structure tuning, active site design, and microenvironment optimization to balance conversion efficiency and lactic acid selectivity in photocatalytic systems.

##### TiO_2_

TiO_2_ is widely recognized for its remarkable chemical stability and strong resistance to photochemical corrosion, enabling it to maintain structural integrity under UV and even visible light irradiation.^[^ ^99^, ^100^ ^]^ These properties make TiO_2_ an ideal candidate for long‐term catalytic applications without significant degradation. However, pristine TiO_2_ possesses a relatively wide bandgap (3.0–3.2 eV), which restricts its light absorption primarily to the UV spectrum and limits its overall photocatalytic efficiency.^[^ ^101^ ^]^ Additionally, unmodified TiO_2_ suffers from rapid recombination of photogenerated charge carriers, further reducing its photocatalytic performance.^[^ ^102^ ^]^ To overcome these challenges, researchers have explored various modification strategies, including element doping, heterostructure engineering, and defect modulation.^[^ ^75^, ^103^ ^]^ These approaches have enhanced TiO_2_’s ability to harness solar energy and significantly improved its H_2_ production efficiency, reinforcing its potential for advanced photocatalytic applications.

CS, which contains ≈50% Fe, served as a valuable Fe source. Utilizing CS, TiO_2_/Fe oxide composite photocatalysts were synthesized via calcination at different temperatures, enabling efficient visible‐light‐driven glucose transformation to lactic acid.^[^ ^104^ ^]^ This approach not only achieved high‐efficiency biomass transformation but also aligned with the principles of a circular economy and sustainable development. Notably, the calcined composites formed robust heterojunctions, significantly enhancing their visible light absorption capacity.^[^ ^104^ ^]^ The resulting composites demonstrated exceptional catalytic performance, achieving 97% glucose conversion and 71% lactic acid production. X‐ray photoelectron spectroscopy (XPS) indicated a positive correlation between calcination temperature and the Ti^3+^/Ti^4+^ ratio. The Z‐scheme heterojunction formed between them facilitated the separation of photogenerated charges and reduced the recombination of photogenerated eˉ‐h^+^ pairs.^[^ ^104^ ^]^

Optimizing TiO_2_’s photocatalytic efficiency for glucose‐to‐lactic acid conversion also required precise control over catalyst morphology. Based on this principle, a fully symmetrical 3DOM TiO_2_‐CdSe heterojunction photonic crystal (s‐TCS) catalyst was designed to efficiently drive the photocatalytic production of lactic acid and H_2_ from glucose.^[^ ^105^ ^]^ The fully symmetrical spherical structure of the photonic crystal ensured uniform light absorption and mass diffusion, thereby elevating both light harvesting and catalytic stability. Furthermore, the incorporation of CdSe NPs onto the TiO_2_ surface led to the formation of a type‐II heterojunction, which significantly enhanced the separation efficiency of photogenerated eˉ‐h^+^ pairs, further boosting photocatalytic performance.^[^ ^105^ ^]^ As a result, the catalyst exhibited outstanding stability and superior light absorption capabilities, achieving a glucose conversion of 95.9%, a lactic acid selectivity of 94.3%, and a lactic acid yield of 96.4%. Notably, this study was the first to reveal that the photocatalytic conversion of glucose followed a third‐order reaction mechanism involving four photogenerated h^+^.

##### Others

**Figure 18:**
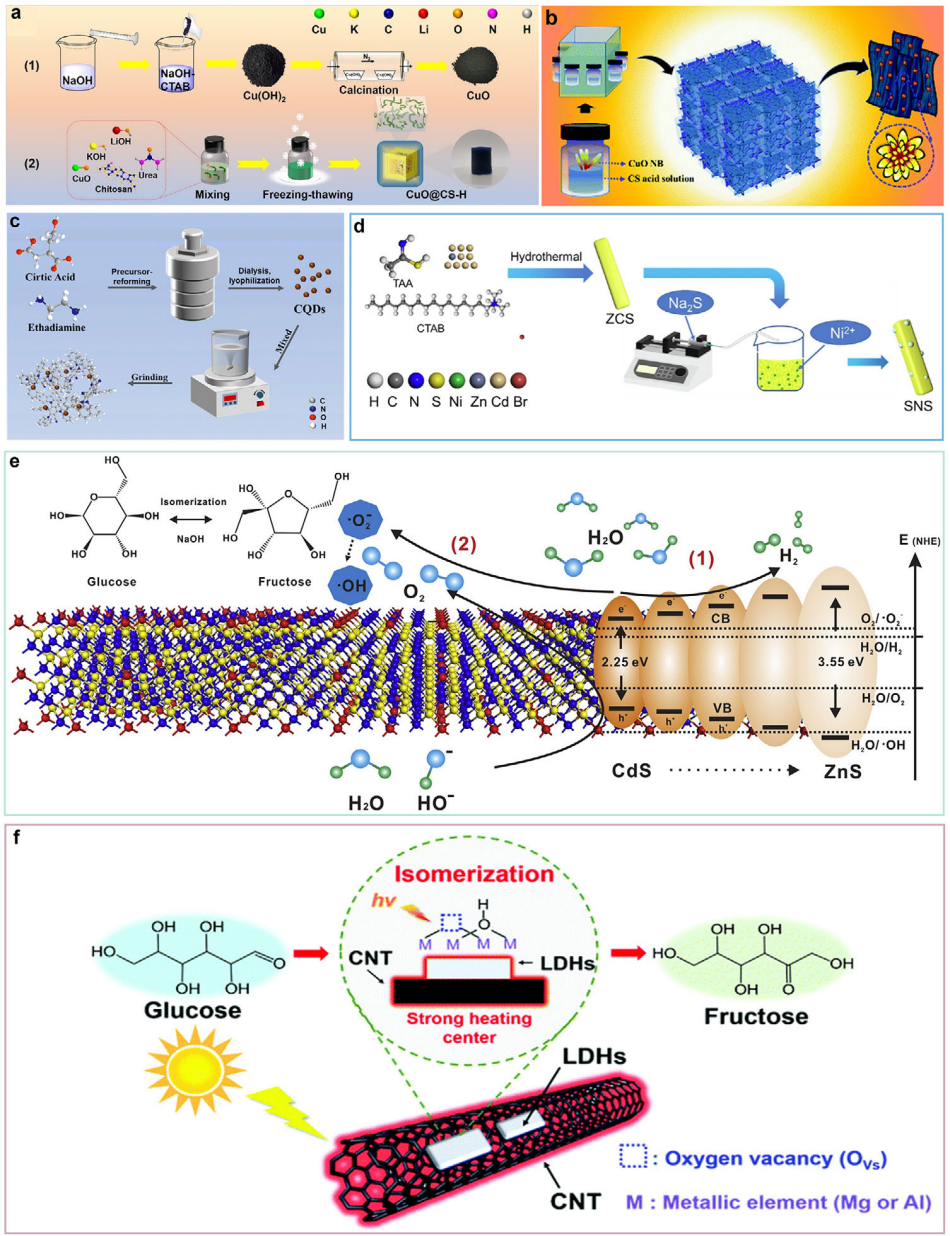
Scheme illustration of a) CuO@CS‐H preparation (Reproduced with permission. Copyright 2021, Elsevier),^[^ ^106^ ^]^ b) preparing Cu/Cu_2_O/CuO@CA (Reproduced with permission. Copyright 2021, Royal Society of Chemistry),^[^ ^107^ ^]^ c) synthesis of CQDs@_4_CzIPN (Reproduced with permission. Copyright 2022, Royal Society of Chemistry),^[^ ^108^ ^]^ and d) preparation of SNS (Reproduced with permission. Copyright 2023, Elsevier).^[^ ^109^ ^]^ e) Reaction pathways for photocatalytic oxidation of glucose. Reproduced with permission.^[^ ^110^ ^]^ Copyright 2021, Cell. f) Photothermal conversion mechanism of glucose. Reproduced with permission.^[^ ^111^ ^]^ Copyright 2022, Royal Society of Chemistry.

CuO NPs were uniformly dispersed in chitosan hydrogel using the freeze‐thaw method (**Figure** **18**a). Exploiting the alkalinity and porous structure of chitosan. The resulting CuO‐chitosan hydrogel composite (CuO@CS‐H) exhibited enhanced stability and adsorption capacity for reactants.^[^ ^106^ ^]^ When applied to photocatalytic lactic acid production from monosaccharides (xylose/glucose), near‐quantitative conversion (≈100%) was achieved. In this reaction system, the alkaline nature of chitosan was used to create a mildly alkaline condition, thereby avoiding the need for high‐concentration alkali solutions required in traditional methods.^[^ ^106^ ^]^ Additionally, the comparatively small bandgap of CuO (≈1.8 eV) facilitated efficient visible light harvesting and charge carrier generation, leading to improved photocatalytic performance. In the same year, the research group designed and synthesized a floral Cu/Cu_2_O/CuO composite material via a freeze‐drying and calcination strategy, which was further uniformly embedded into the carbon nanosheets of a flexible carbon aerogel (Cu/Cu_2_O/CuO@CA) (Figure 18b).^[^ ^107^ ^]^ In this catalyst, the different valence and conduction band positions of Cu_2_O and CuO facilitated the separation of photogenerated eˉ and h^+^, thereby improving photocatalytic performance. The dynamic interconversion between Cu_2_O and CuO also eliminated the need for additional functional materials.^[^ ^107^ ^]^ Moreover, the carbon nanosheets, acting as a support, provided excellent electrical conductivity and mechanical strength, enhancing the stability and photocatalytic activity of the catalyst. The catalyst's surface properties and porous architecture were simultaneously enhanced, promoting efficient reactant adsorption and product transport.^[^ ^107^ ^]^

Carbon quantum dot‐decorated 4CzIPN (CQDs@4CzIPN) was prepared through a single‐step synthesis approach, where CQDs were anchored onto the 1,2,3,5‐tetra(carbazol‐9‐yl)‐4,6‐dicyanobenzene matrix (Figure 18c).^[^ ^108^ ^]^ In this composite, 4CzIPN functioned as a novel organic semiconductor with a straightforward synthesis process, a high molar absorption coefficient, and tunable emission wavelengths. Meanwhile, CQDs, as 0D carbon‐based nanomaterials, offered outstanding photonic characteristics, high aqueous dispersibility, minimal toxicity, eco‐compatibility, and cost‐effectiveness. The incorporation of CQDs@4CzIPN significantly extended its visible light absorption range of the composite to 578 nm and reduced the bandgap from 2.2 eV (pristine 4CzIPN) to 2.0 eV.^[^ ^108^ ^]^ These enhancements led to a remarkable photocatalyst, achieving a lactic acid yield of 96.9% within 30 min at 70 °C.

Zn_0.1_Cd_0.9_S (ZCS) possessed an appropriate bandgap (2.38 eV) and exhibited excellent photocatalytic performance.^[^ ^109^ ^]^ NiS NPs were grown in situ on the surface of ZCS nanorods employing a chemical bath deposition process to build a heterojunction structure (Figure 18d). In this configuration, NiS efficiently captured photogenerated eˉ and promoted H_2_ generation. By optimizing the loading amount of NiS, the photocatalytic performance of the ZCS‐NiS (SNS) composite could be significantly enhanced.^[^ ^109^ ^]^

A homojunction photocatalyst based on the Zn_1‐x_Cd_x_S solid solution was designed through bandgap engineering and a biphasic (ZB and WZ) structural approach, enabling the simultaneous production of H_2_ and lactic acid via photocatalytic glucose reforming.^[^ ^110^ ^]^ Introducing a ZB‐WZ biphasic structure in this catalyst formed a twinned superlattice, which promoted the effective separation of photogenerated eˉ and h^+^. Notably, by adjusting the Zn‐to‐Cd ratio, the bandgap structure of the catalyst could be optimized, thereby enhancing its visible light absorption and product selectivity. In the Zn_1‐x_Cd_x_S homojunction system, photoinduced charge carriers mediated glucose conversion by generating ROS, including •O_2_ˉ and •OH (Figure 18e). These ROS facilitated lactic acid production from glucose. Specifically, under alkaline conditions, the process began with the isomerization of glucose to fructose and the ring‐opening of glucose and fructose. Subsequently, fructose was hydrolyzed in the environment with ROS present. Through a sequence of reactions, this hydrolysis process culminated in the generation of lactic acid.^[^ ^110^ ^]^ Additionally, HCOOH was detected, presumably originating from glucose oxidation at either the *α*‐ or *β*‐position. Notably, the products existed in their anionic forms (lactate and formate). Given the negatively charged surface of the photocatalyst, these anions were rapidly released from the catalyst surface upon formation, preventing over‐oxidation.^[^ ^110^ ^]^

The carbon nanotube/LDH (CNT/LDH) composite catalyst facilitated low‐temperature glucose‐to‐lactate conversion through photothermal activation.^[^ ^111^ ^]^ In this system, CNTs exhibited strong near‐infrared light absorption and high photothermal conversion efficiency, functioning as effective photothermal materials. Meanwhile, LDHs, which feature strong alkaline sites and the ability to generate photo‐induced OVs, served as the active component. During the photocatalytic process, CNTs acted as localized heating centers under illumination, providing the necessary thermal energy for the reaction.^[^ ^111^ ^]^ Simultaneously, LDHs absorb OH‾ ions under alkaline conditions to form strong alkaline sites, which promote the sequential transformation of glucose to lactic acid through isomerization and follow‐up reactions (Figure 18f). Moreover, EPR analysis indicated that OVs formed in LDHs under light irradiation acted as Lewis acid sites. This significantly promoted the isomerization process from glucose to fructose. Consequently, a lactic acid yield of up to 88.6% with a selectivity of 90.0% was achieved.^[^ ^111^ ^]^

**Figure 19:**
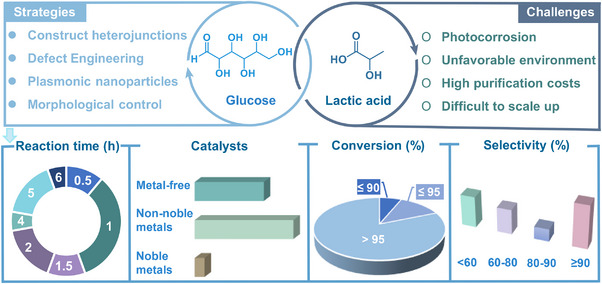
Strategies and challenges in designing photocatalysts for lactic acid production from glucose.

***Summary***. As shown in Table S4 (Supporting Information), recent advancements in the photocatalytic preparation of lactic acid from glucose have made significant strides, particularly in catalyst design and reaction mechanisms. Innovations such as heterojunction structures, defect engineering in carbon nitrides, and the incorporation of plasmonic nanoparticles have substantially improved charge separation, light absorption, and product selectivity (**Figure** **19**). Notably, some studies have achieved lactic acid selectivity exceeding 70%. Additionally, mechanistic insights gained through radical scavenging experiments and DFT calculations have provided a deeper understanding of the roles of ROS and the C1─C2 bond cleavage pathway in glucose conversion. However, several challenges remain. Catalyst stability is a major concern, as structural changes and photo‐corrosion can compromise long‐term performance. Reaction conditions, such as reliance on alkaline environments or organic solvents, limited industrial scalability and environmental sustainability. Moreover, the separation and purification of lactic acid from reaction mixtures remain complex and costly, with by‐products posing challenges to product purity. Future research should focus on developing more robust catalysts, optimizing reaction conditions to minimize environmental impact, and improving separation techniques to enhance the feasibility of photocatalytic glucose‐to‐lactic acid conversion for practical applications.

### 3.5 Glycerol and Amino Acids

Efficient photocatalyst designs have been fruitfully employed in the production of high‐value compounds by glucose photo‐reforming, including glycerol and amino acids. This advancement offers a promising framework for the subsequent valorization of glucose via green, mild, and sustainable pathways.

#### 3.5.1 Glycerol

By combining boron‐doped carbon nitride (BCM‐CN) with nucleophilic dimethyl sulfoxide, a collaborative photocatalytic system was designed to produce glycerol efficiently and selectively through glucose photocatalytic conversion.^[^ ^112^ ^]^ DFT studies combined with material characterization showed that the improved BCM‐CN simultaneously boosted visible light harvest and charge separation while mapping the formation pathway of the important 1,3‐dihydroxyacetone intermediate.^[^ ^112^ ^]^ This study offered novel perspectives on photocatalytic biomass photocatalytic transformation, particularly demonstrating great potential for selective cleavage of the C3─C4 bond and nucleophilic addition reactions.

#### 3.5.2 Amino Acids

**Figure 20:**
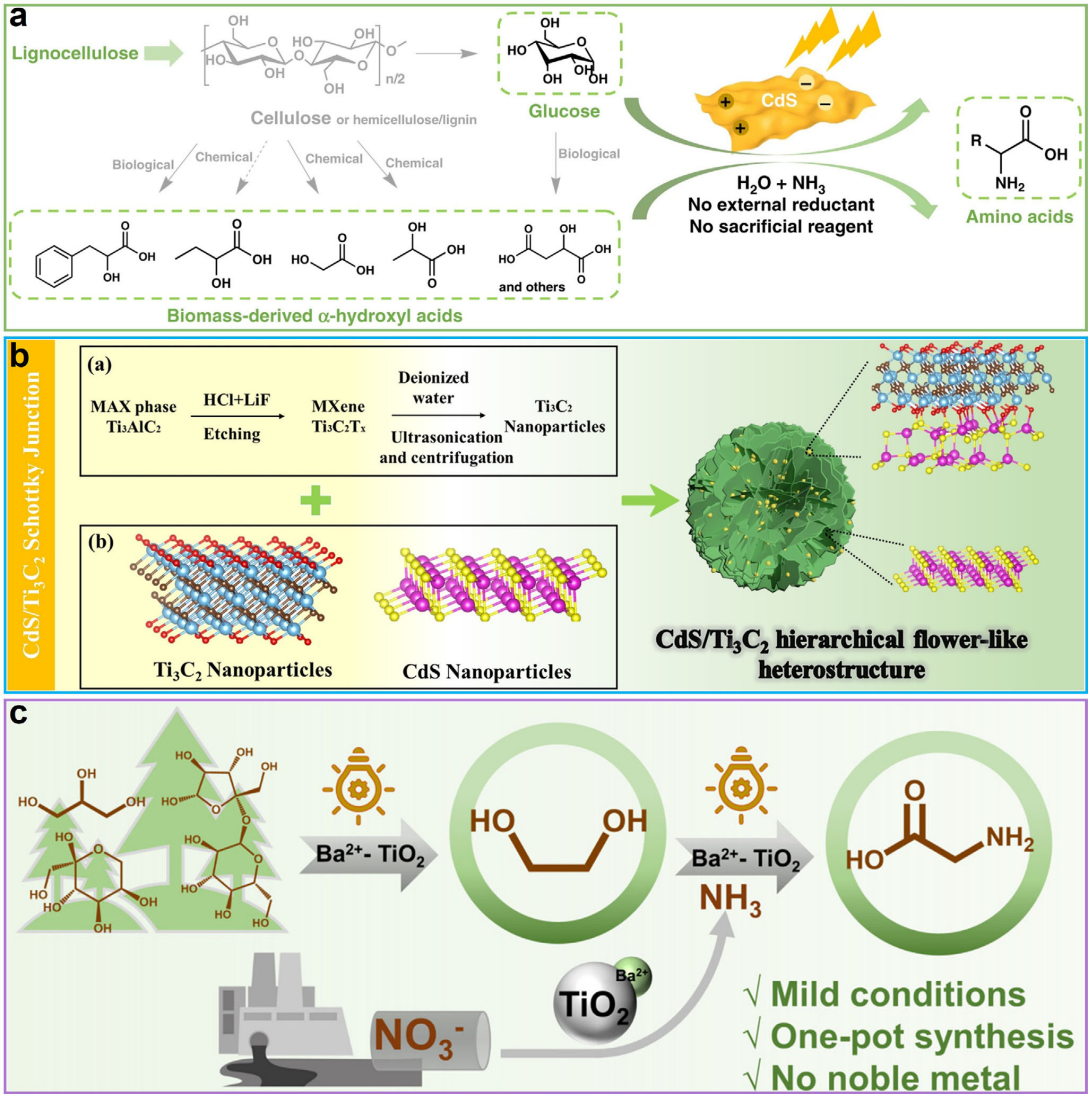
a) Schematic representation of photocatalytic reductive amination to prepare amino acids from glucose. Reproduced with permission.^[^ ^114^ ^]^ Copyright 2020, Springer Nature. b) Schematic fabrication of CdS/Ti_3_C_2_ heterostructure. Reproduced with permission.^[^ ^113^ ^]^ Copyright 2022, Wiley. c) Schematic diagram of photoconversion of biomass and nitrate into glycine. Reproduced with permission.^[^ ^116^ ^]^ Copyright 2024, American Chemical Society.

Amino acids, as fundamental building blocks of living organisms, are widely utilized in various fields, including nutrition, agriculture, polymer synthesis, and the pharmaceutical industry.^[^ ^113^, ^114^ ^]^ Currently, the production of amino acids predominantly relies on fermentation methods, which are associated with several limitations, such as prolonged reaction times, intricate separation processes, high energy consumption, and the exclusive generation of L‐configured amino acids.^[^ ^115^ ^]^ In contrast, the visible‐light‐driven photocatalytic approach, employing ultrathin CdS nanosheets as a catalyst (**Figure** **20**a), provided a green and efficient route for the one‐step conversion of glucose to alanine under mild conditions (50 °C and 1 bar N_2_).^[^ ^114^ ^]^ Notably, the introduction of alkaline additives (e.g., NaOH) promotes glucose‐to‐lactate conversion, consequently enhancing alanine production.^[^ ^114^ ^]^ This innovative strategy enabled sustainable amino acid synthesis using biomass‐based glucose under environmentally benign conditions.

Han et al. constructed a 3D hierarchical CdS/Ti_3_C_2_ MXene Schottky junction through surface engineering and heterojunction design (Figure 20b).^[^ ^113^ ^]^ By precisely controlling the crystal orientation of CdS, they effectively reduced its surface energy, steering the catalytic pathway toward alanine formation while suppressing the generation of unwanted byproducts. Their investigation into the photocatalytic mechanism demonstrated that eˉ migrated from CdS to Ti_3_C_2_, creating an efficient charge transfer channel.^[^ ^113^ ^]^ This charge separation mechanism facilitated the rapid migration of photogenerated eˉ to Ti_3_C_2_. As a result, it augmented the overall efficiency of the reaction. Furthermore, Li et al. utilized Ba^2+^‐modified TiO_2_ (Ba^2+^‐TiO_2_) as a photocatalyst (Figure 20c), leveraging Ba^2+^ incorporation to modify TiO_2_ surface characteristics and enhance photogenerated eˉ‐h^+^ pairs separation.^[^ ^116^ ^]^ This strategy enabled the selective production of glycine from the conversion of biomass and nitrate under mild conditions. In this process, nitrate served as a nitrogen source and played a modulator of •OH levels, preventing excessive oxidation of biomass‐derived alcohols and thereby improving glycine selectivity.^[^ ^116^ ^]^

In the context of photocatalytic glucose conversion to amino acids, catalyst design strategies have been strategically tailored to address efficiency and selectivity, culminating in three key modification approaches with distinct advantages. Ultrathin CdS nanosheets leverage their high specific surface area and enhanced visible‐light absorption, while alkaline additives promote glucose‐to‐lactate conversion as a pivotal intermediate, enabling one‐step alanine synthesis under mild conditions with minimized energy consumption. Hierarchical CdS/Ti_3_C_2_ MXene Schottky junctions advance this by controlling CdS crystal orientation to reduce surface energy, establishing an efficient electron transfer pathway from CdS to Ti_3_C_2_ that suppresses byproducts and steers the reaction toward alanine via optimized charge separation. Meanwhile, Ba^2+^‐doped TiO_2_ modifies surface properties to enhance photogenerated carrier separation, with nitrate serving dual roles as a nitrogen source and •OH regulator to prevent overoxidation of biomass‐derived intermediates, thus boosting glycine selectivity in glucose‐nitrate conversions. Collectively, these modifications—encompassing nanostructure engineering, heterojunction construction, and ion doping—synergize to optimize charge dynamics, reaction microenvironments, and substrate adsorption, providing a robust framework for balancing amino acid yield and selectivity in photocatalytic biomass valorization.

***Summary***. Overall, glucose is used for the photocatalytic transformation into other high‐value small molecules, including glycerol and amino acids. These processes have shown excellent performance. Therefore, glucose shows significant promise as an alternative feedstock for small‐molecule synthesis. However, as indicated in Table S5 (Supporting Information), research on the photocatalytic transformation of glucose remains relatively limited. Developing advanced photocatalytic approaches is therefore essential for optimizing glucose utilization.

## 4 Bio‐Derived Fuels

### 4.1 H_2_

The glucose‐based photocatalytic H_2_ production system has attracted increasing attention as a potential solution to pressing energy and environmental challenges.^[^ ^117^, ^118^ ^]^ This approach enables the efficient utilization of biomass resources and facilitates the generation of clean energy. Unlike conventional H_2_ production methods, which are often energy‐intensive and contribute to significant carbon emissions, photocatalytic H_2_ generation operates under mild conditions with minimal environmental impact.^[^ ^119^ ^]^ Given its high hydrogen‐to‐carbon ratio, glucose is an ideal precursor for H_2_ production.^[^ ^120^ ^]^ Utilizing glucose as an electron donor in H_2_ generation facilitates clean energy production and contributes to wastewater treatment, presenting a dual benefit for sustainable development.

#### 4.1.1 Mechanism

**Figure 21:**
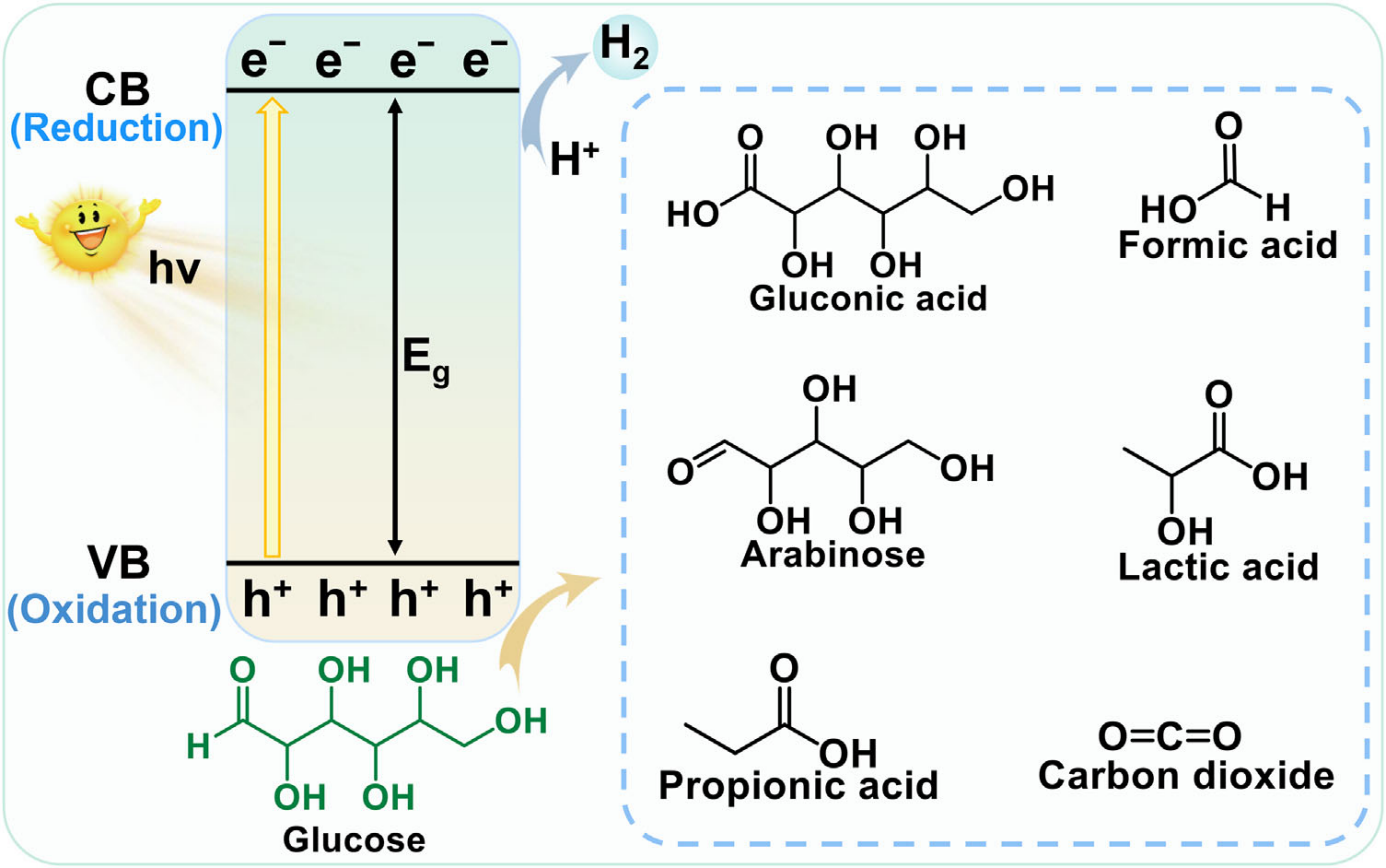
Schematic reaction mechanism of photocatalytic conversion of glucose into H_2_.

As presented in **Figure** **21**, the H_2_ evolution pathway from glucose begins with photoexcitation of the catalyst, producing CB eˉ and VB h^+^.^[^ ^29^, ^121^ ^]^ Photoholes can directly oxidize glucose molecules and react with water to form •OH, further facilitating glucose oxidation.^[^ ^122^ ^]^ The oxidation process yields various products, including arabinose, glucuronic acid, HCOOH, lactic acid, and propionic acid, while CO_2_ may accumulate linearly over time.^[^ ^12^, ^123^ ^]^ Meanwhile, photoelectrons in the CB reduce H^+^ in water, leading to H_2_ generation.^[^ ^124^ ^]^ Some studies have also suggested that H_2_ can be derived from glucose itself.^[^ ^125^ ^]^ Therefore, the design of photocatalysts for glucose‐derived H_2_ production primarily focuses on achieving efficient charge separation, enhanced light absorption, and strong stability.

#### 4.1.2 Catalyst Design

Researchers in the field of photocatalytic H_2_ production from glucose are primarily focused on developing and optimizing high‐performance photocatalysts. Strategies such as rational catalyst design, detailed structure‐activity relationship studies, and systematic reaction condition optimization were being applied to enhance the efficiency of photocatalytic H_2_ generation.^[^ ^63^, ^126^ ^]^ Among these factors, the photocatalyst's spectral response characteristics critically determine visible‐light‐driven H_2_ generation efficiency. According to the semiconductor bandgap equation (Eg = 1240/λg eV), an ideal photocatalyst should have a bandgap of less than 3.1 eV to effectively capture visible light.^[^ ^127^, ^128^, ^129^ ^]^ This characteristic is now widely recognized as a key design criterion for developing advanced photocatalytic materials.

##### g‐C_3_N_4_

g‐C_3_N_4_, with a bandgap of 2.7 eV, has attracted considerable attention owing to its biocompatibility and exceptional stability.^[^ ^130^, ^131^ ^]^ Pristine g‐C_3_N_4_ was prepared via thermal condensation of dicyandiamide, followed by oxidative treatment with HNO_3_ to obtain oxidized g‐C_3_N_4_ (o‐g‐C_3_N_4_).^[^ ^132^ ^]^ This modified material exhibited a remarkable 27 times greater activity than unmodified g‐C_3_N_4_, which was attributed to its expanded surface area, enabling homogeneous Pt distribution during photochemical deposition. Additionally, O doping and ultrasonic exfoliation induced electron redistribution between C and N atoms, promoting π‐bond delocalization and strengthening interfacial coupling, thereby improving redox activity.^[^ ^132^ ^]^ Incorporating Pt as a co‐catalyst further enhanced the material's proton reduction capability. Optimal performance was achieved with 3 wt.% Pt, balancing catalytic activity with minimal photon absorption interference.^[^ ^132^ ^]^ The exceptional photocatalytic performance of o‐g‐C_3_N_4_ in environmental water systems highlights its potential for real‐world applications, particularly in sustainable H_2_ production using natural sunlight and ambient water sources.

Similarly, the noble metal Pd has attracted considerable attention in photocatalytic H_2_ generation owing to its outstanding catalytic performance.^[^ ^133^, ^134^ ^]^ Chinnu et al. synthesized Pd NP‐fabricated g‐C_3_N_4_ nanosheets using thermal exfoliation and the wet impregnation method.^[^ ^121^ ^]^ The porous structure and high specific surface area of the g‐C_3_N_4_ nanosheets provided abundant active sites, enhancing reactant adsorption and improving reaction kinetics. The incorporation of PdNPs significantly enhanced g‐C_3_N_4_’s photocatalytic performance through broadening light absorption, facilitating charge separation, and increasing the density of active sites.^[^ ^121^ ^]^ The research team determined that the optimal Pd loading for maximizing H_2_ production was 0.2 wt.%. Excessive Pd loading caused nanoparticle aggregation, resulting in diminished catalytic activity. Consequently, the catalyst demonstrated exceptional photocatalytic H_2_ production performance under visible light illumination, reaching a maximum H_2_ evolution result of 1839.84 µmol g^−1^ h^−1^. A detailed mechanistic investigation identified three contributing factors to the activity enhancement: 1) PdNPs‐induced surface plasmon resonance broadening the visible light utilization, 2) efficient eˉ trapping by Pd NPs reducing charge recombination, and 3) the increased number of active sites introduced by PdNPs, which facilitated proton reduction.^[^ ^121^ ^]^

**Figure 22:**
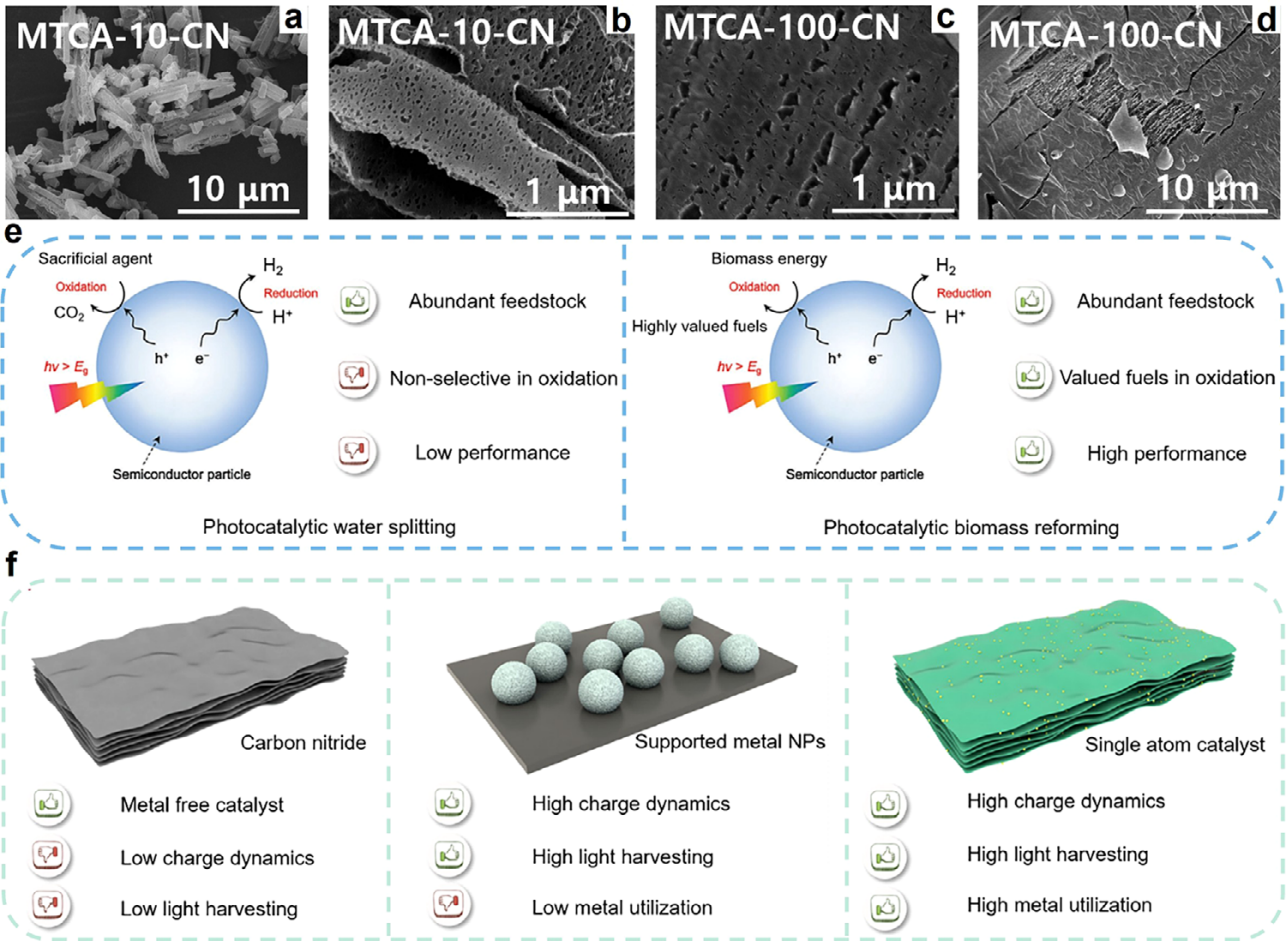
a–d) Scanning electron microscopy (SEM) images of MTCA‐10‐CN and MTCA‐100‐CN. Reproduced with permission.^[^ ^137^ ^]^ Copyright 2021, Elsevier. e) Principles of photocatalytic H_2_ production. f) Schematic design of Pt‐MCNN catalysts. Reproduced with permission.^[^ ^138^ ^]^ Copyright 2023, Elsevier.

Dimensional tuning serves as a powerful strategy to optimize key catalytic properties, including nanoscale morphology, photon capture capacity, charge carrier dynamics, and interfacial energetics.^[^ ^135^, ^136^ ^]^ To address the challenge of rapid charge carrier recombination in g‐C_3_N_4_, Lee et al. developed an innovative approach utilizing melamine (M) and trithiocyanuric acid (TCA) as precursors.^[^ ^137^ ^]^ Through thermal polycondensation, they successfully synthesized macroscopic g‐C_3_N_4_ single crystals (MTCA‐CN) with precisely controlled dimensions ranging from 10 to 100 µm. During polycondensation, the molecular precursors maintained their macroscopic properties while undergoing significant transformations in microstructure, electronic structure, and photocatalytic performance. The Pt‐modified photocatalytic system demonstrated excellent H_2_ efficiency under AM 1.5G irradiation during glucose conversion. The study revealed that size modulation led to the formation of MTCA‐100‐CN, which exhibited enhanced graphitic stacking and reduced in‐plane structural order (Figure 22a–d).^[^ ^137^ ^]^ Although MTCA‐100‐CN displayed relatively weaker light absorption, its elevated HOMO energy level improved its oxidative capability during glucose oxidation, thereby enhancing hydrogen production efficiency. Furthermore, sulfur doping further refined its electronic structure, leading to enhanced photocatalytic activity.^[^ ^137^ ^]^ Thus, it is evident that tuning the band structure of catalysts is crucial for enhancing the catalytic performance of materials.

Characterized by atomically dispersed metal centers, SACs demonstrate unparalleled catalytic advantages, including maximized atomic efficiency, numerous accessible active sites, and wide reaction compatibility.^[^ ^139^, ^140^ ^]^ To improve the efficiency of photocatalytic hydrogen production, Zhang's team engineered a novel catalyst through atomic‐level Pt deposition on ultrathin g‐C_3_N_4_ nanosheets (MCNN), leveraging the distinctive advantages of SACs to enhance photocatalytic performance.^[^ ^138^ ^]^ The incorporation of Pt SAs induced a red shift in the light absorption edge, thereby improving visible light absorption and enhancing photocatalytic activity. Additionally, the Pt‐N coordination structures formed between SAs and the g‐C_3_N_4_ matrix facilitated interfacial electron transport, enabling the Pt‐MCNN‐3.0% catalyst to exhibit outstanding photocatalytic H_2_ production activity under visible light.^[^ ^138^ ^]^ Notably, in the H_2_ production system utilizing glucose as a sacrificial agent, valuable platform molecules were simultaneously generated (**Figure** **22**e). Furthermore, modifying carbon nitride materials with SAs decoration presents a cost‐effective strategy for enhancing photocatalytic efficiency (Figure 22f).^[^ ^138^ ^]^

To further enhance the efficiency of photocatalytic H_2_ production using single‐metal catalysts, Ding et al. designed a novel Au‐Pt heterostructure co‐catalyst, leveraging the synergistic effects of bimetallic co‐catalysts to improve the H_2_ generation rate.^[^ ^123^ ^]^ This heterostructure features an Au core (≈3 nm) surrounded by multiple Pt islands (≈1 nm), creating an optimized catalytic interface. Both experimental and theoretical studies confirmed that the exceptional H_2_ generation activity of the Au‐Pt heterostructure originates from the synergistic interaction between Au and Pt rather than Au's plasmonic effect. This unique architecture enhanced the capture of photogenerated eˉ from g‐C_3_N_4_ and optimized H^+^ adsorption strength, exhibiting high intrinsic activity for H_2_ evolution.^[^ ^123^ ^]^

Lead‐free perovskites, specifically DMASnBr_3_ and PEA_2_SnBr_4_, were selected as key components of the composite material owing to their excellent photon harvesting capability and tunable bandgaps.^[^ ^141^ ^]^ The hybridization of these perovskites with g‐C_3_N_4_ produced micrometer‐sized architectures that enhanced light‐driven catalytic performance. Additionally, the incorporation of Pt as a co‐catalyst introduced a strong Schottky barrier due to its high work function, further improving H_2_ production efficiency.^[^ ^142^ ^]^ DMASnBr_3_/g‐C_3_N_4_ composite achieved a notable H_2_ production performance. Its superior dispersibility and reduced hydrophobicity further contributed to enhanced performance in aqueous environments, demonstrating its potential for efficient and sustainable H_2_ generation.

**Figure 23:**
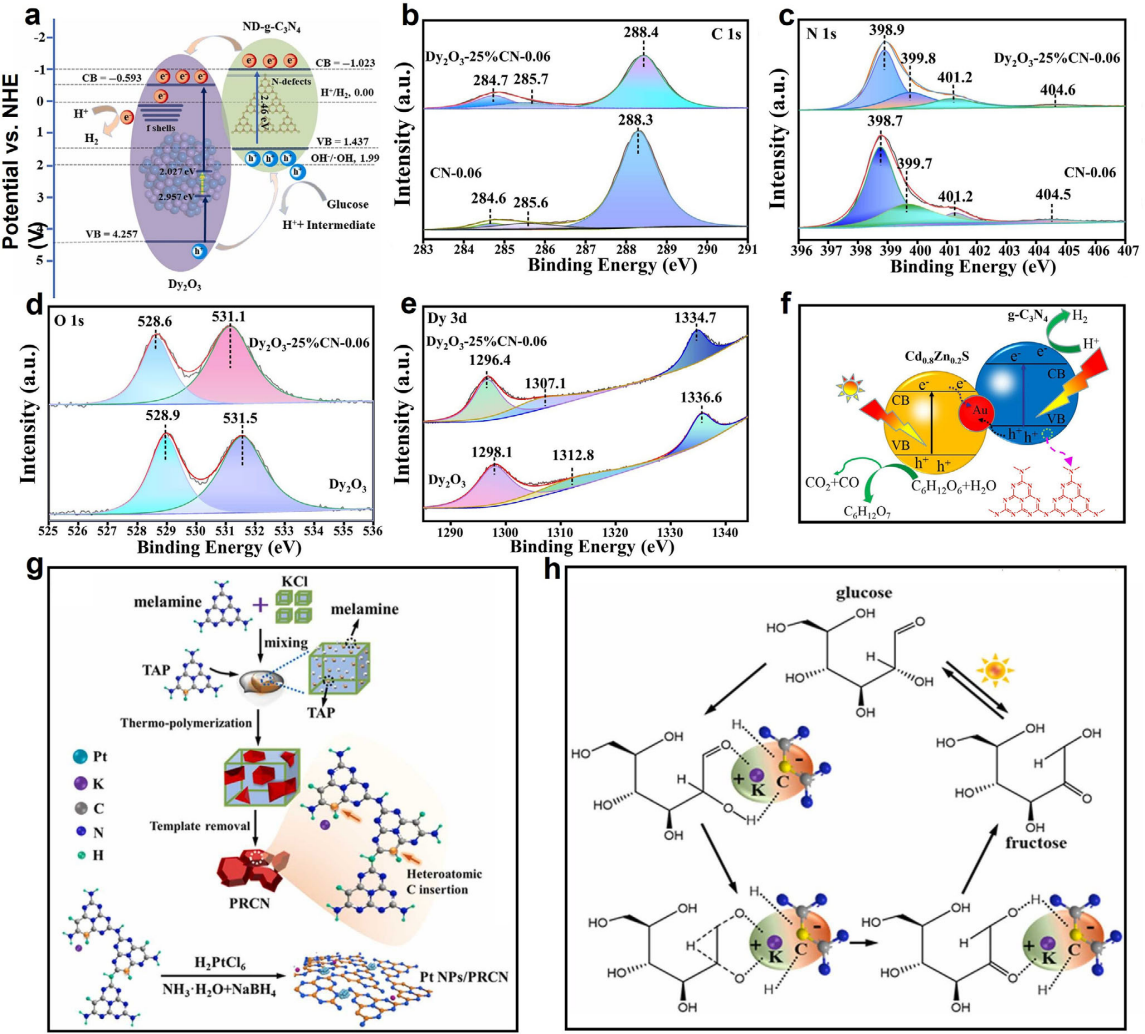
a) Mechanism of H_2_ production by type II heterojunction. XPS spectra of b) C 1 s, c) N 1 s, d) O 1s, e) Dy 3d in CN‐0.06, Dy_2_O_3_, and Dy_2_O_3_/CN photocatalyst. Reproduced with permission.^[^ ^144^ ^]^ Copyright 2024, Elsevier. f) Mechanism of H_2_ production by type Z heterojunction. Reproduced with permission.^[^ ^147^ ^]^ Copyright 2017, Elsevier. g) Schematic illustration of PRCN and Pt NPs/PRCN synthesis procedure. h) Proposed mechanism for isomerizing glucose to fructose. Reproduced with permission.^[^ ^148^ ^]^ Copyright 2022, Elsevier.

Heterostructure engineering represents a fundamental approach to boost semiconductor photocatalysis through optimized interfacial charge transfer.^[^ ^143^ ^]^ In this regard, Jing et al. selected dysprosium oxide (Dy_2_O_3_) as the base catalyst, leveraging its unique 4f electronic structure and optical properties to efficiently trap eˉ and suppress exciton recombination.^[^ ^144^ ^]^ To further optimize the band structure of g‐C_3_N_4_, nitrogen defects (ND) were introduced, creating ND‐g‐C_3_N_4_. Subsequently, Dy_2_O_3_ was combined with ND‐g‐C_3_N_4_ through high‐temperature calcination, forming a type II heterojunction (**Figure** **23**a). XPS and EPR analyses confirmed interfacial electron migration from ND‐g‐C_3_N_4_ to Dy_2_O_3_, where photogenerated eˉ from ND‐g‐C_3_N_4_ migrated to the CB of Dy_2_O_3_ (Figure 23b–e).^[^ ^144^ ^]^ This process significantly promoted charge carrier separation, suppressed eˉ‐h^+^ recombination, and thereby improved H_2_ generation efficiency.

Z‐scheme heterostructure engineering has proven particularly effective for facilitating charge transport dynamics, establishing itself as a key research direction in solar‐driven H_2_ evolution.^[^ ^145^, ^146^ ^]^ To develop an efficient Z‐scheme photocatalyst, Au NPs were first loaded onto g‐C_3_N_4_ via chemical reduction, followed by the deposition of CdxZn_1‐x_S solid solutions through photochemical deposition, forming a CdxZn_1‐x_S/Au/g‐C_3_N_4_ heterojunction (Figure 23f).^[^ ^147^ ^]^ Among the synthesized composites, Cd_0.5_Zn_0.2_S/Au/g‐C₃N₄ exhibited exceptional photocatalytic performance. Beyond its enhanced photocatalytic efficiency, this Z‐scheme heterojunction significantly suppressed the formation of CO and CO_2_. Mechanistic investigations demonstrated that the basic surface properties of CdxZn_1‐x_S facilitated glucose adsorption while inhibiting the dehydration of intermediate products (such as HCOOH derivatives), thereby reducing CO and CO_2_ production.^[^ ^147^ ^]^ In this Z‐scheme system, Au NPs functioned as eˉ mediators, promoting charge separation between g‐C_3_N_4_ and CdxZn_1‐x_S, which contributed to superior photocatalytic performance. Additionally, the CdxZn_1‐x_S solid solution expanded photon harvesting into the visible region and improved charge carrier energetics for enhanced reductive activity.

A salt‐templating approach was employed to introduce carbon and potassium dopants into polymericPRCN), generating transient Lewis acid‐base pairs (Figure 23g).^[^ ^148^ ^]^ These sites played distinct roles: the Lewis base sites facilitated glucose deprotonation, while the Lewis acid sites activated its aldehyde group, enhancing the overall reaction efficiency (Figure 23h). Additionally, the deposition of high work‐function metals, such as PtNPs, on the PRCN surface led to forming a Schottky junction. This junction dramatically enhanced charge carrier dynamics, boosting H_2_ production efficiency. Notably, incorporating C and K‐induced charge redistribution within PRCN generated electron‐enriched C sites (acting as Lewis base sites) and sites bearing positive charge (functioning as Lewis acids).^[^ ^148^ ^]^ Leveraging these synergistic mechanisms, the Pt/PRCN catalyst achieved H_2_ evolution rates exceeding those of PRCN and g‐C_3_N_4_ by a factor of 3.4‐fold and 43.8‐fold, respectively, demonstrating exceptional catalytic activity.^[^ ^148^ ^]^

In photocatalytic H_2_ evolution, C_3_N_4_ materials have been modified through diverse strategies to enhance catalytic efficiency. These approaches include 1) morphological engineering through surface treatments (oxidation, exfoliation, etc.) to expand surface area and modulate electron distribution, 2) SAs or bimetallic loading (e.g., Pt and Au‐Pt) for enhanced photoelectron trapping via synergistic effects, 3) heterojunction construction with tailored Schottky barriers to facilitate charge transfer, and 4) elemental doping that generates Lewis acid‐base pairs to promote both glucose activation and Schottky junction formation. Collectively, these modification strategies establish a robust foundation for the rational design of efficient and stable photocatalytic systems, propelling advancements in sustainable H_2_ production and its practical applications.

##### TiO_2_

**Figure 24:**
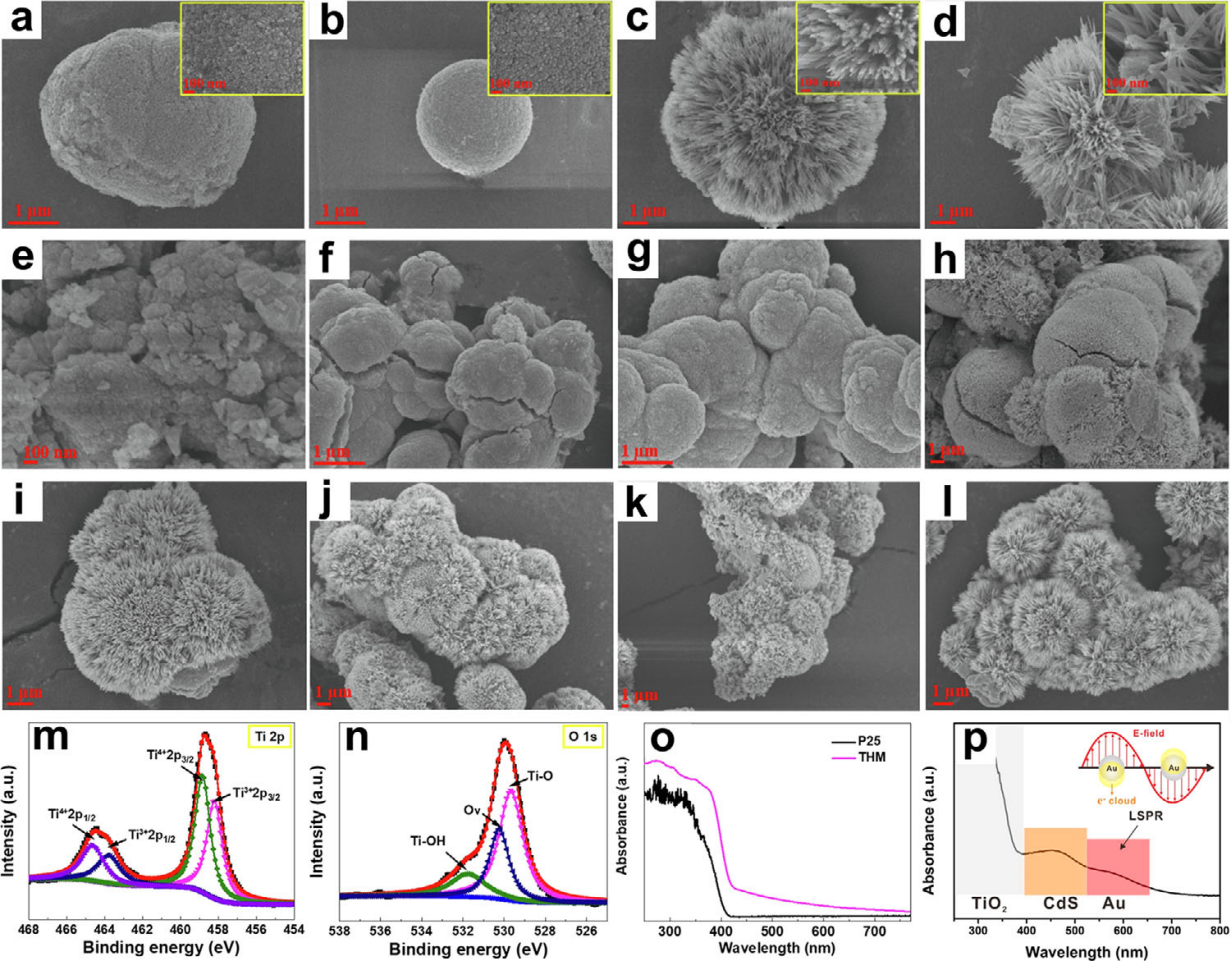
a–d) SEM images of samples synthesized with TiCl_4_ concentrations of 6.25, 9.09, 14.29, and 20 wt.%, respectively. e–l) SEM images of samples synthesized at different lengths of solvothermal time of 0.5, 0.75, 1, 1.5, 2.5, 6, 12, and 48 h. m–n) XPS spectra of Ti 2p and O 1s. o) UV–vis DRS of THM. Reproduced with permission.^[^ ^149^ ^]^ Copyright 2023 Elsevier, MDPI. p) UV–vis DRS of 3DOM TiO_2_‐Au‐CdS. Reproduced with permission.^[^ ^150^ ^]^ Copyright 2022, MDPI.

By integrating morphological control with defect engineering, Shi et al. developed a novel 3D hierarchical TiO_2_ microsphere (THM) photocatalyst.^[^ ^149^ ^]^ During synthesis, key parameters such as TiCl_4_ concentration, reaction time, and temperature were systematically optimized. Their findings suggested that THM formation follows a “nucleation‐dissolution, recrystallization, and assembly” mechanism (**Figure** **24**a–l).^[^ ^149^ ^]^ As a result, they successfully synthesized THM with a surface enriched in OVs and unsaturated Ti atoms (Figure 24m–n). In photocatalytic H_2_ production from glucose, the unsaturated Ti atoms and OVs in THM established LMCT interaction with the hydroxyl oxygen atoms of glucose, facilitating efficient charge separation.^[^ ^149^ ^]^ This interaction promotes efficient eˉ transfer while suppressing non‐selective radical‐based oxidation reactions. UV–vis DRS analysis confirmed that the introduction of OVs and Ti^3+^ narrowed the bandgap of THM (Figure 24o), enhancing visible light absorption and leading to an impressive H_2_ evolution rate of 9440 µmol g^−1^ h^−1^. Additionally, Zhong et al. synthesized a 3DOM TiO_2_ catalyst using a colloidal template method.^[^ ^150^ ^]^ The catalyst was then modified through the sequential loading of Au NPs via chemical reduction and CdS via chemical bath deposition, forming a TiO_2_‐Au‐CdS ternary system. The incorporation of CdS established a typical type II heterojunction, while Au NPs further enhanced visible light absorption through their LSPR effect. Acting as eˉ transfer bridges (Figure 24p), Au NPs improved charge separation efficiency, thereby significantly boosting the quantum efficiency of photocatalysis.^[^ ^150^ ^]^

Using a hydration‐dehydration method and a one‐pot oxidation approach, Silva and colleagues synthesized TiO_2_‐decorated carbon nanotube (CNT‐TiO_2_), followed by the deposition of Au, Pt, Ir, and Pd.^[^ ^29^ ^]^ Among these, the Pt‐decorated catalyst demonstrated superior photocatalytic performance relative to unmodified TiO_2_. Investigation of the reaction mechanism indicated that H_2_ generation efficiency correlated strongly with carbohydrate structural complexity and H/C atomic ratios. Notably, the Pt/(CNT‐TiO_2_)ox‐473 catalyst demonstrated superior H_2_ evolution efficiency when complex saccharides, such as cellobiose, were used as substrates. Additionally, the photocatalytic activity varied significantly depending on the TiO_2_ crystal phases.^[^ ^151^, ^152^ ^]^ Specifically, rutile and brookite exhibited higher activity in glucose conversion and H_2_ generation, whereas anatase displayed stronger oxidative properties. The incorporation of Pt as a co‐catalyst further facilitated proton reduction, thereby enhancing hydrogen production efficiency.^[^ ^151^ ^]^ Fu's research team systematically investigated structural and operational parameters affecting glucose photo‐reforming by designing TiO_2_ catalysts loaded with various noble metals.^[^ ^152^ ^]^ Their findings demonstrated that the incorporation of noble metals significantly enhanced the H_2_ generation rate, with Pd and Pt exhibiting the highest catalytic activity. Critical parameters, including TiO_2_ polymorph, Pt concentration, gaseous environment, and solution acidity, significantly influenced hydrogen generation efficiency. Oxygen exposure and low pH conditions substantially suppressed activity, while mild alkalinity (pH = 11) proved ideal, achieving peak H_2_ production at 4580 µmol g^−1^ h^−1^.^[^ ^152^ ^]^ The adsorption characteristics and reaction activities of glucose isomers play a crucial role in determining photocatalytic efficiency. As demonstrated by Zhuo and coworkers, Pt/TiO_2_ catalysts exhibited 2.3‐fold higher activity for *α*‐D‐glucose conversion compared to *β*‐D‐glucose, attributed to preferential C1‐hydroxyl coordination at active sites.^[^ ^153^ ^]^ This activity difference was most pronounced under neutral conditions but became less distinct in acidic and alkaline environments. Mechanistic studies suggested that under weakly alkaline conditions, the TiO_2_ surface acquired a negative charge (>TiO⁻), which enhanced the adsorption of glucose anions.^[^ ^152^ ^]^ Compared to *β*‐D‐glucose, *α*‐D‐glucose demonstrated stronger adsorption on the catalyst surface and more efficient interactions with •OH, leading to a higher H_2_ evolution rate.^[^ ^153^ ^]^

The Pd/TiO_2_ catalyst efficiently hydrogenated nitrobenzene compounds into aniline derivatives under UV irradiation while simultaneously producing arabinose, erythrose, HCOOH, and glycolic acid from glucose.^[^ ^122^ ^]^ During the photocatalytic process, photogenerated h⁺ oxidized glucose, leading to the formation of •OH that further facilitated glucose oxidation. Simultaneously, photogenerated eˉ reduced H^+^ in water to generate H_2_ and form hydrogenated Pd NPs (H‐PdNPs), which served as active species for the hydrogenation reaction. Notably, nitrobenzene compounds preferentially adsorbed onto the Pd/TiO₂ surface and underwent sequential reduction via H‐PdNPs, following the reaction pathway: nitrobenzene → nitrosobenzene → hydroxylamine → aniline.^[^ ^122^ ^]^

Through innovative catalyst design, Tkachenko et al. successfully deposited Pt oxide particles onto TiO_2_ via controlled hydrolysis of Pt(IV) hydroxide in a sulfuric acid solution.^[^ ^154^ ^]^ By precisely regulating the loading and oxidation state of Pt, they achieved efficient H_2_ production in ethanol and glucose solutions. Their findings indicated that excessive Pt deposition acts as recombination centers for eˉ‐h^+^ pairs, ultimately reducing photocatalytic activity. Additionally, Bellardita et al. discovered that low‐frequency ultrasonic treatment reduced the bandgap of TiO_2_ to 3.04 eV.^[^ ^155^ ^]^ They further investigated the effects of photo‐deposited Pt, Ag, Rh, and Pd on the degradation of 4‐nitrophenol (4‐NP) and the photocatalytic H_2_ production from glucose oxidation. Due to differences in work function (Pt: 5.65 eV > Pd: 5.12 eV > Rh: 4.98 eV > Ag: 4.26 eV), Pt exhibited the highest H_2_ evolution efficiency (2.17 mM).^[^ ^155^ ^]^ Notably, ultrasonic treatment increased the hydroxyl group (−OH) content on the TiO_2_ surface, creating effective h^+^ capture centers that boosted generated •OH and enhanced both organic oxidation and H_2_ production. Furthermore, Ramis et al. demonstrated that temperature and pressure were critical factors influencing H_2_ production efficiency in TiO_2_‐based catalytic systems. Through comprehensive optimization, the researchers identified 80 °C and 4 bar as ideal operational parameters, achieving exceptional hydrogen evolution at 14,690 µmol g^−1^ h^−1^.^[^ ^156^ ^]^

Fluorine incorporation markedly enhanced the catalytic efficiency of Pd/TiO_2_ systems.^[^ ^125^ ^]^ Vaiano's group demonstrated that TiO_2_ fluorination generated surface fluoride species (≡Ti‐F) while simultaneously increasing the surface −OH content.^[^ ^125^ ^]^ Additionally, fluorination effectively inhibited the transition to the rutile phase, thereby preserving the anatase phase's inherent high surface area and stability—key factors for sustained catalytic activity.^[^ ^125^ ^]^ Unlike conventional glucose‐based H_2_ production systems that typically operate in alkaline environments, the fluorinated Pd/TiO_2_ catalyst achieved a significantly higher H_2_ evolution rate (590 µmol g^−1^ h^−1^) under acidic conditions (pH = 2). Meanwhile, neutral conditions were found to be more favorable for glucose adsorption and degradation. Sandoval's team developed a dual‐functionalization protocol involving simultaneous sulfation and fluorination to enhance surface acidity in both Lewis and Brønsted configurations. This dual modification further increased the H_2_ content in the gas‐phase products, demonstrating its potential for optimizing photocatalytic H_2_ production efficiency.^[^ ^157^ ^]^

Addressing rapid charge recombination and poor reforming efficiency in traditional TiO_2_ systems, Eqi's team engineered a biomimetic sea urchin‐shaped architecture decorated with Ni‐Au alloy nanoparticles.^[^ ^158^ ^]^ This catalyst achieved highly efficient co‐production of H_2_ and arabinose, with an H_2_ evolution rate of 6391.86 µmol g^−1^ h^−1^ and an arabinose selectivity of 36.54%. Notably, the catalyst was synthesized through a simple one‐step impregnation method, yielding Ni_x_Au_0.5‐x_/TiO_2_ with varying Ni and Au loadings. The structural engineering successfully extended TiO_2_’s photon harvesting capability to visible wavelengths.^[^ ^158^ ^]^ Structural analysis revealed that Ni and Au NPs were uniformly distributed on the TiO_2_ nanorods, maintaining close contact with the TiO_2_ surface. The LSPR effect of Au NPs enhanced visible light absorption, while the high work function of Ni NPs (5.3 eV) facilitated C─C bond cleavage.^[^ ^158^ ^]^ The synergistic effect of these bimetallic nanoparticles significantly boosted photocatalytic activity, resulting in hydrogen evolution rates that were 118.57, 30.78, and 1.65 times higher than those of pure TiO_2_, Ni_0.5_/TiO_2_, and Au_0.5_/TiO_2_, respectively^[^ ^158^ ^]^ In a separate study, Ma et al. synthesized Er^3+^:YAlO_3_ up‐conversion luminescent materials via sol‐gel and calcination methods^[^ ^159^ ^]^ These materials effectively converted visible light into UV light, thereby activating TiO_2_ to generate photogenerated eˉ‐h^+^ pairs. Additionally, Pt was deposited onto the membrane surface as a co‐catalyst using an impregnation method, facilitating eˉ transfer and enhancing H_2_ production efficiency. As a result, the H_2_ yield reached 153.9 mmol, significantly surpassing that of pure Pt‐TiO_2_ membranes.^[^ ^159^ ^]^

**Figure 25:**
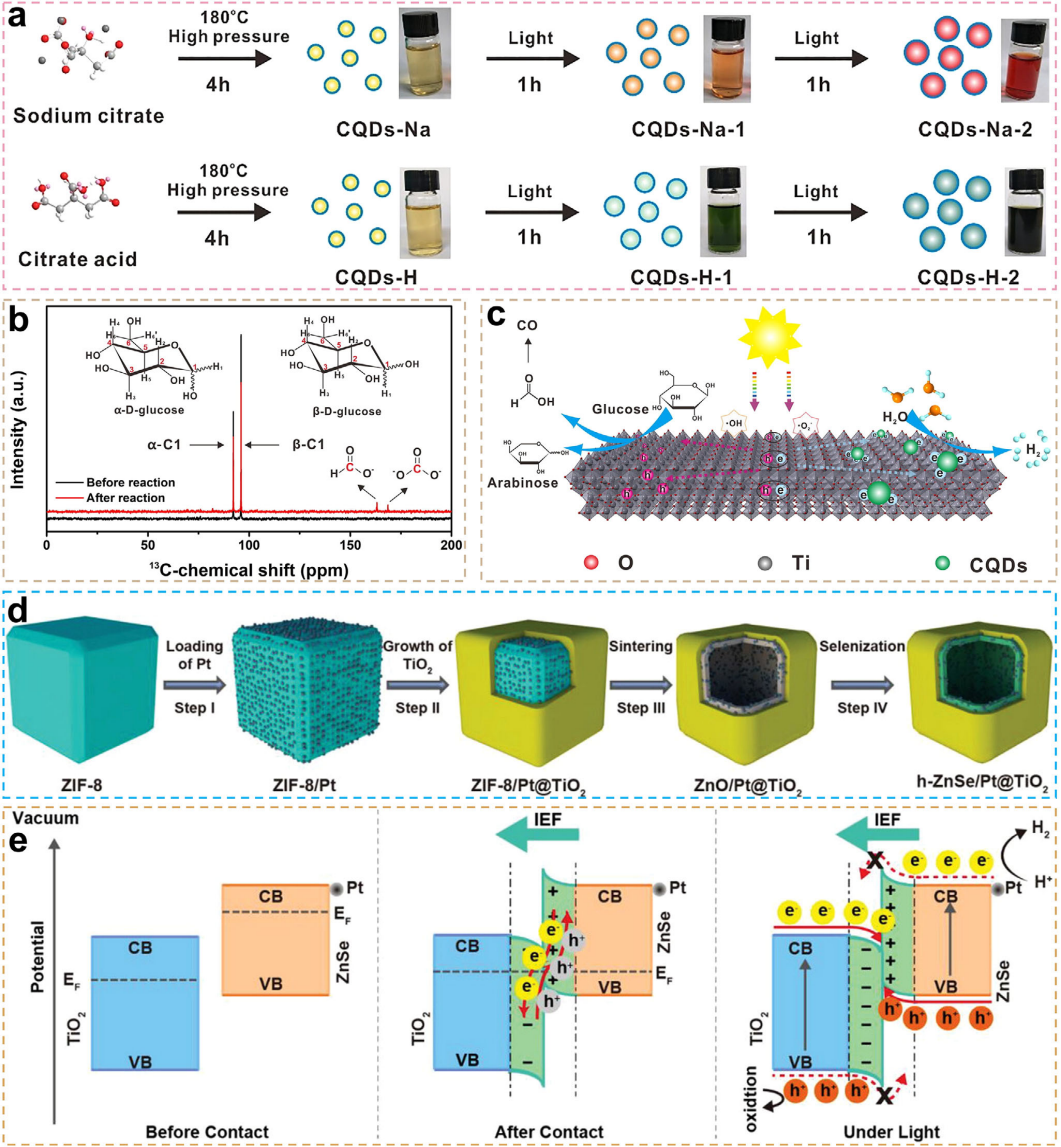
a) Scheme diagram of preparing CQDs by modulating light using sodium citrate and citric acid. b) ^13^C NMR before and after spectra of glucose. c) Corresponding mechanism of glucose photoreaction. Reproduced with permission.^[^ ^124^ ^]^ Copyright 2022, Elsevier. d) Schematic diagram of the synthesis of h‐ZnSe/Pt@TiO_2_. e) S‐scheme charge transfer mechanism between ZnSe and TiO_2_ in h‐ZnSe/Pt@TiO_2_. Reproduced with permission.^[^ ^160^ ^]^ Copyright 2023, Wiley.

Developing TiO_2_‐based catalysts modified with non‐precious metals is important to enhance economic feasibility. In this context, Zhao et al. designed a CQD‐modified TiO_2_ composite (CQDs/TiO_2_), utilizing the exceptional optoelectronic characteristics of carbon quantum dots to boost charge.^[^ ^124^ ^]^ Notably, the particle size of CQDs could be precisely tuned by adjusting the illumination duration. Zhao et al. demonstrated that CQDs synthesized from sodium citrate (CQDs‐Na) exhibited a color transition from bright yellow to orange and red under light irradiation, with particle size increasing from 4.25 to 7.00 nm.^[^ ^124^ ^]^ Similarly, CQDs derived from citric acid (CQDs‐H) changed from bright yellow to light green and dark green, accompanied by a size increase from 2.70 to 4.75 nm (**Figure** **25**a). As the particle size increased, fluorescence intensity decreased, while photocatalytic activity improved.^[^ ^124^ ^]^ More importantly, N doping and particle size variations in CQDs were found to significantly influence charge separation efficiency. Furthermore, the research group employed ^13^C isotope labeling to investigate possible bond cleavage pathways of glucose (Figure 25b). The results demonstrated that glucose undergoes C1─C2 bond cleavage to produce arabinose and formic acid. Consequently, a detailed reaction mechanism for hydrogen production from glucose was elucidated (Figure 25c).^[^ ^124^ ^]^

Employing the successive ionic layer adsorption and reaction technique, Roongraung's team fabricated CdS‐TiO_2_ heterostructures by sequentially depositing cadmium sulfide on both nanoparticle and nanotube forms of titanium dioxide.^[^ ^103^ ^]^ Under both UV and simulated solar irradiation, CdS/TiO_2_ NTs exhibited superior glucose conversion and H_2_ evolution efficiency, achieving an H_2_ yield of 0.138 mmol. The performance improvement mainly resulted from the nanotubular architecture's substantially increased surface area relative to nanoparticles, offering more accessible catalytic sites. Additionally, increasing the CdS content effectively reduced the bandgap of TiO_2_, further improving its photocatalytic performance.^[^ ^103^ ^]^ Moreover, You et al. designed and synthesized a hollow‐structured h‐ZnSe/Pt@TiO_2_ catalyst (Figure 25d), where ZnSe functioned as a reducing photocatalyst and TiO_2_ as an oxidizing photocatalyst.^[^ ^160^ ^]^ During the photoreaction, eˉ‐h^+^ transfer followed an S‐scheme mechanism, which promoted charge separation and preserved the strong redox capabilities of ZnSe and TiO_2_. Additionally, the interfacial electric field generated between ZnSe and TiO_2_ promoted directional charge transfer while suppressing eˉ‐h^+^ recombination, thereby improving photocatalytic performance (Figure 25e). Notably, Pt was deposited on ZnSe as a co‐catalyst, effectively shortening the electron transport path and further boosting H_2_ evolution performance.^[^ ^160^ ^]^

Key TiO_2_ modification strategies for glucose‐driven H_2_ production include: i) 3D morphology tuning and OVs generation for improved substrate adsorption, ii) plasmonic heterojunctions (e.g., Au/TiO_2_) for enhanced light utilization, iii) tailored metal cocatalysts (Pt, Ni‐Au) to accelerate proton reduction, iv) anion doping (F/S) for acid‐stable performance, and v) phase engineering with up‐converters to expand excitation wavelengths. This systematic optimization addresses all critical aspects of the photocatalytic process.

##### Metal Sulfides

Metal sulfides represent a highly versatile class of semiconductor photocatalysts.^[^ ^161^ ^]^ The cations in these sulfide semiconductors are primarily metals with d^10^ electronic configurations, including Zn, Cd, Mo, In, and Mn.^[^ ^161^, ^162^ ^]^ In these compounds, hybridization occurs between the atomic orbitals of the metal cations and S^2^ˉ anions, leading to the formation of numerous closely spaced molecular orbitals that constitute the CB and VB.^[^ ^163^ ^]^ In particular, the CB minimum and VB maximum are largely determined by the outermost atomic orbitals of the constituent elements.^[^ ^163^ ^]^ Moreover, compared to metal oxides, most metal sulfides demonstrate reduced oxidation capabilities due to the higher energy position of the VB formed by S3p orbitals relative to the O 2p orbitals in oxides. As a result, metal sulfides typically exhibit enhanced reduction capabilities, lower oxidation potentials, and narrower bandgaps.^[^ ^164^ ^]^

**Figure 26:**
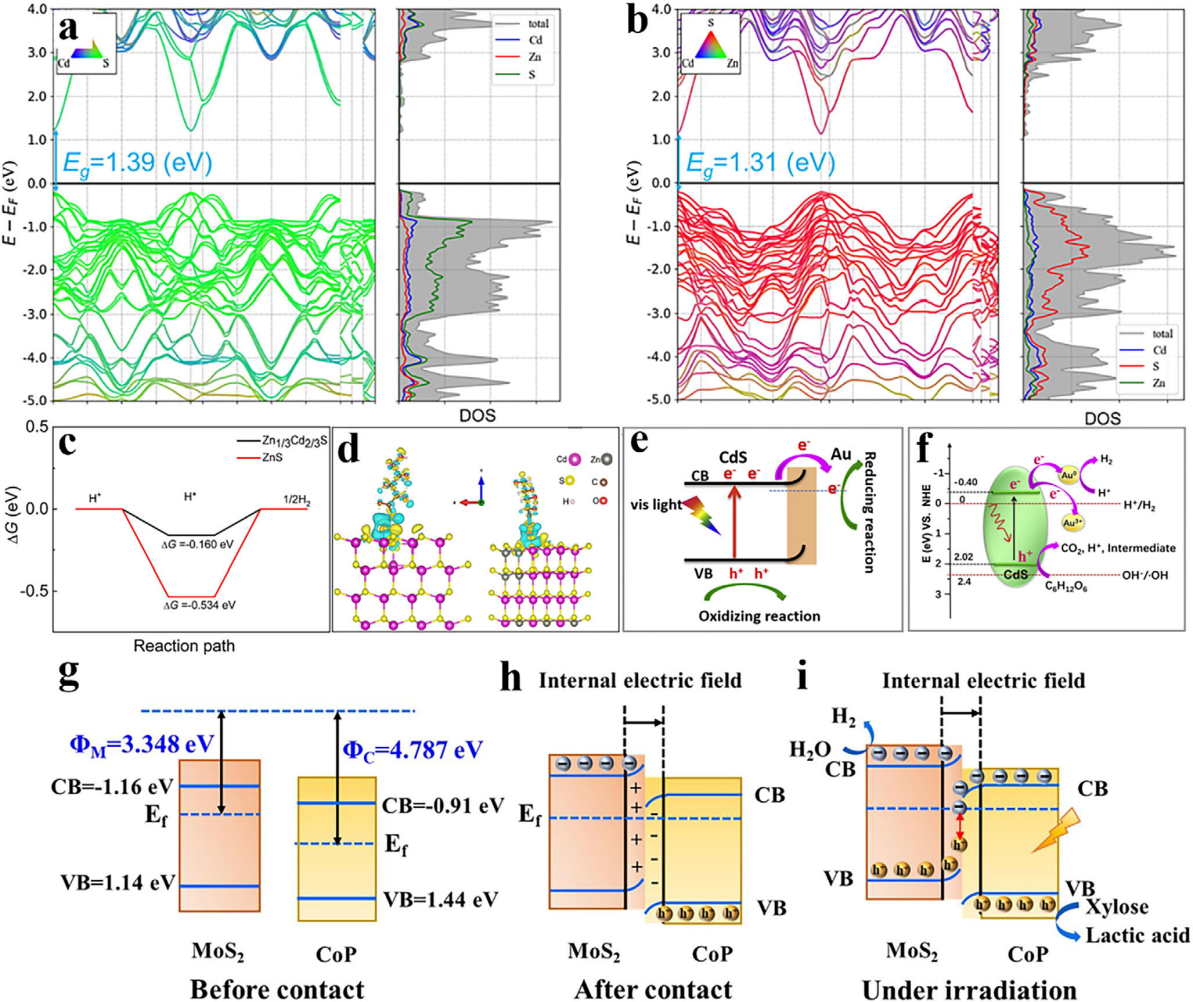
Schematic diagrams of bandgap calculation for a) Zn_1/3_Cd_2/3_S ZB and b) Zn_1/3_Cd_2/3_S WZ. c) Gibbs free energy for H_2_ evolution reaction of ZnS and Zn_1/3_Cd_2/3_S. d) Charge density difference of CdS and Zn_1/3_Cd_2/3_S. Reproduced with permission.^[^ ^165^ ^]^ Copyright 2023, Elsevier. e) Schematic diagram of charge transfer for Au/CdS. f) Schematic illustration for the mechanism of photocatalytic H_2_ evolution over Au/CdS. Reproduced with permission.^[^ ^166^ ^]^ Copyright 2020, Elsevier. g–i) Schematic demonstrations of the charge transfer in the S‐scheme heterojunction of CoP@MoS_2_‐2. Reproduced with permission.^[^ ^167^ ^]^ Copyright 2025, Elsevier.

The Zn_0.3_Cd_0.7_S catalyst was synthesized by the hydrothermal method with optimized Zn and Cd ratios, in which the homojunction of hexagonal wurtzite (WZ) and cubic zinc blende (ZB) phases was found. This distinctive structure significantly enhances the separation of photogenerated charge carriers, while its marigold‐like 3D architecture provides abundant catalytic centers between the catalyst and substrate, thereby elevating photocatalytic performance.^[^ ^165^ ^]^ Kang et al. investigated the band structure of this homojunction using DFT calculations. Their results revealed that upon homojunction formation, the CB edges of the ZB and WZ phases align, leading to band bending (**Figure** **26**a,b). This facilitated the transfer of eˉ from WZ to ZB, where they participated in proton reduction to generate H_2_.^[^ ^165^ ^]^ Simultaneously, photogenerated h^+^ in the ZB VB migrated to the WZ VB, where they oxidized glucose into lactic acid. The thermodynamic analysis further demonstrated that Zn_0.3_Cd_0.7_S exhibited a more favorable H_2_ evolution potential than ZnS (Figure 26c,d).^[^ ^165^ ^]^ The catalyst demonstrated exceptional efficiency, producing H_2_ at 13,640 µmol g^−1^ h^−1^ while converting 96.40% of glucose with 76.80% lactic acid yield.^[^ ^165^ ^]^ Separately, Au nanoparticle decoration of CdS nanorods through sequential solvothermal treatment significantly improved photoactivity.^[^ ^166^ ^]^ During this process, surface Au^3+^ ions were reduced to form Au NPs, establishing a Schottky junction at the CdS interface. This junction facilitated charge separation and enhanced charge transfer, significantly improving photocatalytic H_2_ evolution efficiency (Figure 26e,f).^[^ ^166^ ^]^

Due to the well‐matched CB alignment between CdS and MoS_2_, photogenerated eˉ in CdS could efficiently transfer to MoS_2_ through the heterojunction. This eˉ migration prevented charge accumulation in CdS, suppressed photo‐corrosion, and enhanced the overall stability of the catalyst.^[^ ^168^ ^]^ Pure MoS_2_ naturally adopted a flower‐like microsphere structure with an average diameter of 4.3 µm with numerous nanosheets.^[^ ^168^ ^]^ As the CdS content increased, the diameter of the CdS/MoS_2_ composite microspheres decreased. At a CdS loading of 40%, the composite exhibited excellent dispersion and a uniform flower‐like morphology. By simultaneously tuning both the bandgap and morphology, the catalyst achieved an impressive H_2_ evolution.^[^ ^168^ ^]^ Notably, NiS could serve as an alternative to MoS_2_ in constructing a NiS/CdS heterostructure, with additional loading of MoS_2_ and NiPx as co‐catalysts.^[^ ^169^ ^]^ During the photoreaction, eˉ transferred via a Z‐scheme pathway, which promoted the efficient separation of photogenerated eˉ‐h^+^ pairs. This strategy provided valuable insights into achieving multicomponent synergy and enhancing charge carrier dynamics for improved photocatalytic performance.

Enhancing CdS photocatalysis under visible light has prompted exploration of diverse heterostructure designs.^[^ ^170^, ^171^ ^]^ Among them, the Cd_x_Zn_1‐x_S solid solution, formed by integrating CdS and ZnS, exhibited outstanding photocatalytic activity.^[^ ^170^ ^]^ The adjustable Cd/Zn stoichiometry in these solid solutions critically influenced their photocatalytic behavior. Notably, under alkaline conditions (0.1 mol L^−1^ NaOH), the surface S species of Cd_0.5_Zn_0.5_S were gradually replaced by O species, resulting in the predominant formation of hZn‐OH/hZn‐Oˉ and hCd‐OH/hCd‐Oˉ surface groups. Simultaneously, in high‐salinity environments, Na^+^ ions were adsorbed onto the catalyst surface, forming inner‐ or outer‐sphere complexes that enhanced surface charge density.^[^ ^170^ ^]^ This microstructural transformation significantly improved glucose adsorption, facilitating the efficient extraction of photogenerated h^+^ and enhancing the photo‐oxidation reaction.^[^ ^171^ ^]^ Furthermore, the 1%Pt/Cd_0.6_Zn_0.4_S/Cd_0.1_Zn_0.9_S, comprising Cd_0.6_Zn_0.4_S and Cd_0.1_Zn_0.9_S phases to form a heterojunction structure that further enhances photocatalytic efficiency.^[^ ^172^ ^]^ Under strongly alkaline conditions (5 M NaOH), the increased dissociation of the substrate enhanced photogenerated charge utilization, leading to improved activity and stability. As a result, the catalyst achieved exceptional H_2_ production efficiency at 3400 µmol g^−1^ h^−1^.

Through a facile solvothermal approach, researchers fabricated a ZnIn_2_S_4_‐based photocatalyst with a protective ZnS shell.^[^ ^173^ ^]^ Incorporating an outer ZnS layer significantly enhanced glucose adsorption, while precise control over ZnS content allowed for systematic tailoring of the ZnIn_2_S_4_ morphology. This tunability results in diverse nanostructures, including microspheres, microtubes, and microribbons, all contributing to improved photocatalytic performance.^[^ ^173^ ^]^ Furthermore, as a cost‐effective alternative, NiS has shown catalytic efficiency rivaling precious metal‐based systems in recent investigations.^[^ ^174^ ^]^ Structural characterization of NiS‐modified Cd_1‐x_Mn_x_S photocatalysts revealed extensive stacking faults, which were vital for charge separation. A systematic investigation into the effect of Mn content variation showed a simultaneous decrease in grain size and unit cell volume, along with a progressive shift of the CB to more negative potentials.^[^ ^174^ ^]^ These synergistic structural and electronic modifications significantly enhanced H_2_ evolution efficiency (13,700 µmol g^−1^ h^−1^).

Using pyrolytic and hydrothermal synthesis, Zhang's team fabricated CoP@MoS_2‐x_ heterostructures with floral morphology. The hierarchical architecture provided an enlarged surface area and abundant catalytic sites, leading to superior activity.^[^ ^167^ ^]^ Furthermore, the CoP/MoS_2‐x_ interfacial junction generated an intrinsic electric field with band alignment modification, promoting directional charge transfer and minimizing eˉ‐h^+^ recombination (Figure 26g–i). These synergistic effects collectively contributed to a substantial enhancement in photocatalytic efficiency.^[^ ^167^ ^]^

Metal sulfide catalysts for glucose‐driven photocatalytic H_2_ production have been optimized through strategic modifications: Homojunction engineering in ZnCdS has created phase interfaces for effective band bending. Heterostructures such as CdS/MoS_2_ have leveraged band alignment to facilitate electron transfer and Z‐scheme dynamics, with multicomponent systems demonstrating enhanced synergistic effects. CdxZn_1‐x_S solid solutions have successfully tuned both bandgap and surface oxygen species under alkaline conditions. Surface modification with Au NPs has established Schottky junctions on CdS, while ZnS coating on ZnIn_2_S_4_ has improved substrate adsorption and morphological stability. Hierarchical CoP@MoS_2‐x_ architectures have introduced built‐in electric fields to promote charge transport and reduce recombination. Collectively, these approaches have optimized band structure, charge carrier dynamics, and substrate‐catalyst interactions for efficient H_2_ evolution.

##### Others

Integrating magnetic particles into photocatalysts enabled efficient recovery after photocatalytic reactions using an external magnetic field, offering a cost‐effective strategy for sustainable H_2_ production. This approach significantly reduced operational expenses while enhancing catalyst reusability.^[^ ^175^ ^]^ A notable modification involved doping LaFeO_3_ with the noble metal Ru, which reduced the bandgap of Fe_2_O_3_ to 1.95 eV. This enhancement broadened its light absorption range, enabling efficient utilization of visible and even infrared light for photocatalytic reactions.^[^ ^175^ ^]^ A more economical alternative involved optimizing the amount of citric acid during hydrothermal synthesis. This adjustment influenced the crystal structure, specific surface area, and crystallinity of LaFeO_3_, thereby improving its photocatalytic performance. Studies suggested that citric acid acted as an organic fuel, and during combustion, it created a porous structure that increased the catalyst's specific surface area, ultimately enhancing photocatalytic activity.^[^ ^176^ ^]^ Furthermore, LaFeO_3_ could be modified by depositing Ni onto its surface via photo‐deposition as a cost‐effective substitute for noble metals. While introducing Ni did not alter the orthorhombic perovskite crystal structure of LaFeO_3_, it functioned as an efficient co‐catalyst, facilitating the transfer of photogenerated eˉ.^[^ ^177^ ^]^ The LaFeO_3_/Ni Schottky junction substantially minimized charge recombination, leading to improved photocatalytic performance.^[^ ^177^ ^]^ Structural analysis confirmed Ru^3+^ incorporation into the perovskite lattice while preserving its orthorhombic symmetry, with the bandgap narrowing from 2.12 to 1.72 eV at 2.33% doping. UV–vis spectroscopy demonstrated both bathochromic shifting and intensified absorption, indicating superior visible‐light harvesting.^[^ ^117^ ^]^ As a result, the photocatalyst achieved optimal performance, degrading 70% glucose and yielding H_2_ at 1158 µmol g^−1^ h^−1^.

BYV solid solutions with controlled Bi/Y ratios were synthesized through a solid‐state reaction approach. This process involved the stoichiometric mixing of Bi_2_O_3_, Y_2_O_3_, and NH_4_VO_3_ precursors, followed by high‐temperature calcination.^[^ ^178^ ^]^ Systematic characterization revealed that Y doping in BiVO_4_ promoted a structural phase transition from monoclinic to tetragonal symmetry, resulting in single‐phase BYV solid solutions. This structural transition caused a notable extension of light absorption to 460 nm and decreased bandgap to 2.7 eV, significantly improving visible light absorption efficiency.^[^ ^179^ ^]^ Among high‐performance Bi‐based photocatalysts, Bi_2_WO_6_, a representative Aurivillius‐phase oxide, exhibited superior optical absorption properties and an optimal bandgap of 2.53 eV.^[^ ^179^ ^]^ Nanostructured Bi_2_WO_6_ with a nanosheet morphology demonstrated a specific surface area of 48 m^2^ g^−1^, which improved photon absorption and reactant adsorption, ultimately achieving a remarkable H_2_ evolution rate of 3.05 mmol h^−1^ cm^−2^. Recent progress in catalyst synthesis has demonstrated that ball milling technology, combined with transition metal Cu modification of Bi‐based oxides, enabled efficient glucose oxidation and H_2_ evolution (2600 µmol h^−1^) under simulated solar irradiation.^[^ ^180^ ^]^ The mechanochemical ball milling synthesis approach offered several advantages, including operational simplicity, cost‐effectiveness, solvent‐free processing, and excellent scalability, positioning it as a promising candidate for industrial‐scale photocatalytic applications.^[^ ^181^ ^]^

In‐based materials have shown significant promise in biomass conversion and photocatalytic H_2_ production.^[^ ^182^ ^]^ Research has further highlighted that the selection of In salt precursors was critical in catalyst preparation. For instance, In acetate, In chloride, and In nitrate could produce In_2_O_3_ photocatalysts with distinct micro‐ and nanostructures during hydrothermal synthesis, such as 3D micro‐cubic In_2_O_3_‐MC, 3D micro‐rice‐like In_2_O_3_‐MR, and 2D nanosheet‐like In_2_O_3_‐NP. Among these, the 3D micro‐rice‐like In_2_O_3_‐MR demonstrated the highest photocatalytic H_2_ production activity, outperforming most reported In_2_O_3_‐based materials.^[^ ^182^ ^]^ Additionally, doping with Ni could further enhance the performance of photocatalysts.^[^ ^183^ ^]^ Specifically, incorporating Ni^2+^ into InVO_4_ modified the catalyst in two key ways: 1) bandgap engineering to extend spectral response into the visible range, boosting photon utilization and catalytic activity, and 2) regulating the crystal size and specific surface area of InVO_4_, thereby enhancing substrate adsorption capacity.^[^ ^183^ ^]^

Alkali metal tantalates (MTaO_3_), especially NaTaO_3_, have been extensively investigated for their exceptional water‐splitting performance.^[^ ^184^ ^]^ Studies on the photocatalytic H_2_ generation of La‐doped alkali metal tantalates in glucose solutions revealed that NaTaO_3_ achieved the highest H_2_ production efficiency. The addition of glucose significantly boosted the H_2_ production rate while inhibiting oxygen generation, as glucose was progressively oxidized to carbon dioxide. When employed as a co‐catalyst, NiO demonstrated superior H_2_ production efficiency on NaTaO_3_ compared to Pt, with an optimal loading of 0.2 wt.%.^[^ ^184^ ^]^ Furthermore, recent investigations demonstrated that various layered perovskite‐type oxides and their derivatives, synthesized through solution combustion and organic modification techniques, exhibited enhanced activity in photocatalytic H_2_ generation.^[^ ^185^ ^]^ The primary effects of organic modification on these catalysts included the formation of organic‐inorganic hybrid materials and the expansion of interlayer spacing. These structural modifications promoted the diffusion of reactant molecules into the interlayer regions, thereby improving photocatalytic H_2_ evolution performance.^[^ ^185^ ^]^

Through the rational design of 3DOM CaTiO_3_ (CTO) and the in situ coupling of Zn_0.3_Cd_0.7_S quantum dots (ZCS QDs), a type II heterojunction photocatalyst (3DOM CTO‐ZCS) was successfully synthesized.^[^ ^186^ ^]^ This catalyst exhibited exceptional performance in photocatalytic H_2_ production. The enhanced photocatalytic activity was attributed to the 3DOM structure, which improved light absorption and utilization efficiency but also promoted the transport and diffusion of water and organic molecules.^[^ ^186^ ^]^ Additionally, the partial substitution of Cd^2+^ with Zn^2+^ enhanced the separation and transport of photogenerated charge carriers while mitigating the photo‐corrosion of CdS. Notably, the catalyst avoided the over‐oxidation of glucose to CO_2_, instead selectively converting it into high‐value products, including gluconic acid (with a selectivity of 85.65%) and lactic acid (with a selectivity of 14.36%).^[^ ^186^ ^]^ This selective conversion was attributed to the fact that only photogenerated h^+^ participated as oxidants during the photo‐oxidation process.

In metal‐free mediated H_2_ production from glucose, covalent triazine frameworks synthesized from benzyl halide monomers exhibited a rich microporous structure, high specific surface area, and an optimal bandgap structure.^[^ ^187^ ^]^ These organic frameworks also demonstrated efficient eˉ extraction and separation capabilities, significantly enhancing their photocatalytic performance. Additionally, S‐ and N‐doped graphene quantum dots (SNGQDs) have been successfully utilized as photocatalysts for reforming sugars into H_2_.^[^ ^188^ ^]^ The co‐doping of S and N significantly reduced the bandgap of graphene, enabling visible light absorption. Moreover, introducing amide and amino functional groups during the autoclave hydrothermal treatment enhanced electron resonance and separated photogenerated charges, resulting in outstanding performance in the photocatalytic reforming process.^[^ ^188^ ^]^

**Figure 27:**
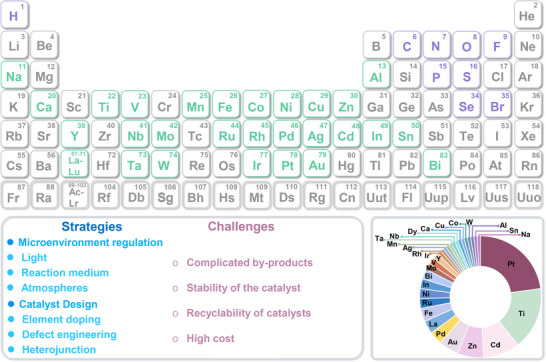
Strategies and challenges in designing photocatalysts for H_2_ production from glucose.

***Summary***. Recent advancements in photocatalytic H_2_ production from glucose have demonstrated significant progress in catalyst design, reaction condition optimization, and mechanistic understanding (**Figure** **27**). Catalyst design innovations, such as heterostructure engineering, defect tuning, and bimetallic/nanocomposite integration, have synergistically enhanced light absorption, charge separation, and active site accessibility—key principles for boosting glucose conversion and H₂ evolution efficiency. As summarized in Table S6 (Supporting Information), these include multi‐component composites like Pt/TiO_2_‐Nb_2_O_5_, surface modifications via fluorination/sulfonation to enhance stability, and nanostructured architectures to expand surface area. These improvements have led to enhanced photocatalytic activity and higher H_2_ generation rates. Reaction conditions have also been optimized through tailored light sources and reaction media, while mechanistic studies clarify charge carrier dynamics and intermediate formation. However, challenges remain: reliance on precious metals (Pt/Pd/Au/Ru) elevates costs, visible light utilization remains suboptimal, and catalyst deactivation from contamination or structural changes hinders durability. Additionally, by‐product interference reduces H₂ selectivity, underscoring the need for further advancements in robust, cost‐effective catalyst systems.

### 4.2 HCOOH

The photocatalytic conversion of glucose to HCOOH is a promising strategy, offering numerous advantages and vast application prospects.^[^ ^189^ ^]^ This process harnesses solar energy to drive the reaction, using renewable glucose as the feedstock. By avoiding the high‐temperature, high‐pressure, and strong acid/alkali conditions typically required in conventional chemical synthesis, it enables the mild, efficient, and environmentally friendly production of HCOOH.^[^ ^26^, ^189^ ^]^ HCOOH is a crucial organic chemical with diverse applications. In the energy sector, it serves as fuel for fuel cells and a hydrogen carrier. In the chemical industry, it is a key raw material for synthesizing various organic compounds.^[^ ^190^, ^191^ ^]^ In agriculture and the food industry, it acts as a feed additive and preservative.^[^ ^192^ ^]^ For environmental protection, it is used in wastewater treatment and as a green solvent.^[^ ^82^ ^]^ In the pharmaceutical field, this compound contributes to medication production and antimicrobial material innovation.^[^ ^193^ ^]^ Therefore, the photocatalytic conversion of glucose to HCOOH provides an innovative pathway for the sustainable production of HCOOH and offers significant support for advancing green chemistry and sustainable development.

**Figure 28:**
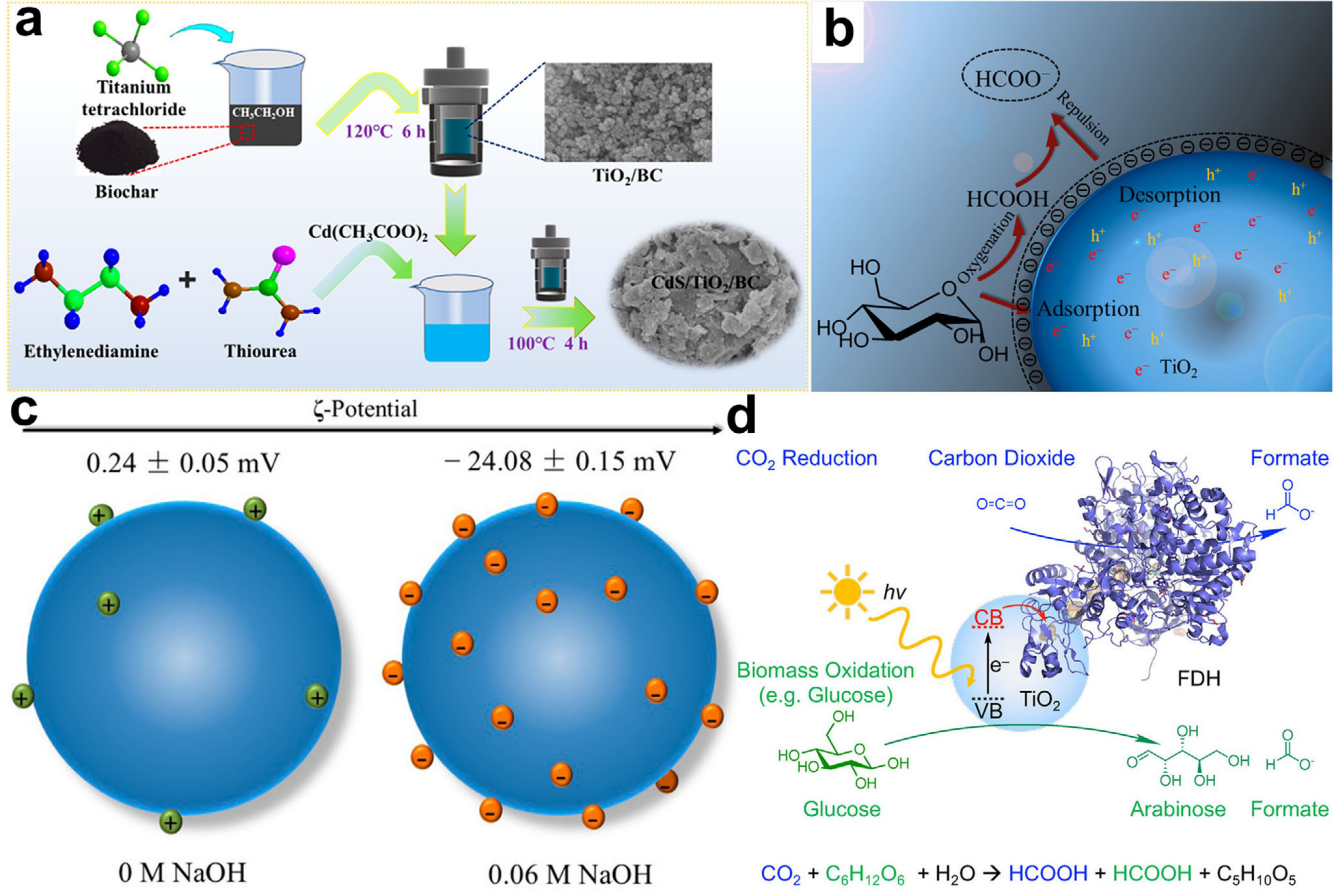
a) Scheme illustration of CdS/TiO_2_/BC preparation. Reproduced with permission.^[^ ^82^ ^]^ Copyright 2024, American Chemical Society. b) Mechanism of the catalytic conversion of glucose to HCOOH by surface negatively charged TiO_2_. c) Zeta potential of TiO_2_ with and without alkali. Reproduced with permission.^[^ ^26^ ^]^ Copyright 2017, American Chemical Society. d) Schematic diagram of selective photocatalytic conversion of CO_2_ and glucose to HCOOH over TiO_2_|FDH. Reproduced with permission.^[^ ^189^ ^]^ Copyright 2023, Wiley‐VCH GmbH.

By harnessing the excellent physicochemical properties of biochar (BC) and the synergistic effects of heterojunctions, Shi et.al. constructed a CdS/TiO_2_/BC heterojunction photocatalyst on a BC support using a simple hydrothermal and calcination method (**Figure** **28**a).^[^ ^82^ ^]^ In this structure, BC acted as the support, offering both extensive surface area and excellent electron transport properties, while also enhancing light absorption (with a red‐shifted absorption edge at 528 nm) and charge separation capabilities.^[^ ^82^ ^]^ These properties allowed the heterojunction to effectively regulate the transformation pathways of glucose by modulating the reaction microenvironment (using NaOH or Na_2_CO_3_). Under NaOH conditions, acetic acid was predominantly produced (with a selectivity of 63.94%), whereas under Na_2_CO_3_ conditions, HCOOH was primarily formed (with a selectivity of 60.29%).

In the photocatalytic conversion of glucose to HCOOH, TiO_2_ generated various ROS and photogenerated h^+^ with strong oxidizing power. These active species could replace traditional oxidants (such as H_2_O_2_ or O_2_), enabling a green photocatalytic biomass transformation process without the need for additional oxidants.^[^ ^26^ ^]^ Notably, the conversion of glucose to HCOOH was primarily driven by enhanced ROS (•O_2_ˉ and •OH) generation under alkaline conditions (Figure 28b).^[^ ^26^ ^]^ Furthermore, in alkaline media, the TiO_2_ surface carries a negative charge, creating a repulsive force with formate ions (HCO_2_ˉ). By adjusting the alkali concentration (e.g., NaOH), the surface charge of TiO_2_ could be modulated, thereby regulating glucose adsorption and product desorption‐adsorption dynamics. This prevented the complete mineralization of HCO_2_ˉ and enhanced the selectivity for HCOOH (Figure 28c).^[^ ^26^ ^]^ Additionally, light irradiation was the key factor driving the conversion of glucose to HCOOH rather than thermochemical effects. In the absence of light, the yield of HCOOH was negligible; however, under irradiation, the yield significantly increased, reaching 35% after 9 h.

Although the amount of HCOOH produced from glucose conversion catalyzed by TiO_2_ could be controlled by modulating the reaction microenvironment, TiO_2_ alone was not capable of driving this reaction under visible light. Interestingly, Bi_2_O_3_ has a narrower bandgap and can absorb visible light, making it an ideal candidate for enhancing photocatalytic efficiency when combined with TiO_2_.^[^ ^194^ ^]^ Research has shown that adjusting the mass ratio of Bi_2_O_3_ to TiO_2_ in the Bi_2_O_3_/TiO_2_ composite to form a heterojunction structure enabled the two components to work synergistically, allowing synergistic effects that extended the spectral response, improved charge separation efficiency.^[^ ^194^ ^]^ Experimental results indicated that the Bi_2_O_3_/TiO_2_ composite exhibited excellent photocatalytic performance and stability at the optimal mass ratio of 1:3. After 4 h of reaction, the glucose conversion reached 65.7%, with arabinose and HCOOH selectivity of 48.3% and 25.6%, respectively.^[^ ^194^ ^]^

To maximize waste utilization, Lam et al. developed a semi‐artificial biohybrid photocatalyst (TiO_2_|FDH).^[^ ^189^ ^]^ Under illumination, this photocatalytic system simultaneously drove CO_2_ reduction and glucose oxidation, combining the products of both reactions into a single HCOOH product via a stoichiometric reaction (Figure 28d). This design allowed for the simultaneous conversion of solid and gaseous waste streams.^[^ ^189^ ^]^ In this catalytic system, TiO_2_ served as the light‐absorbing semiconductor, generating photogenerated eˉ upon light absorption. These eˉ were transferred to the immobilized FDH, which reduced CO_2_ to HCOOH. This approach enabled the coupling of redox reactions within the process. By avoiding oxygen production, which was common in traditional photocatalysis. The design reduced the required thermodynamic driving force and enhanced reaction efficiency, achieving an HCOOH yield of 1.16 mmol g^−1^‐TiO_2_.^[^ ^189^ ^]^

The photo‐oxidation of glucose could be coupled with the reduction of nitrobenzene, enabling the reaction to proceed at room temperature and ambient pressure without the need for external H_2_.^[^ ^195^ ^]^ Under UV light exposure, TiO_2_ facilitated the quantitative conversion of nitrobenzene into aniline. During this process, glucose was converted into products such as HCOOH, arabinose, glycolaldehyde, and lactic acid. Once all the nitrobenzene was consumed, aniline was formed with an 81% yield and was not further oxidized. This was likely because, in the photocatalytic hydrogenation process, sugars served not only as a hydrogen source but also as h^+^ scavengers, preventing the re‐oxidation of aniline by photogenerated h^+^.^[^ ^195^ ^]^ Interestingly, researchers have loaded diverse transition metals, including Pt, Pd, Au, Ag, Cu, and Ru onto TiO_2_ as co‐catalysts via photo‐deposition. However, experimental results indicated that TiO_2_ without metal loading exhibited the highest activity in this system. This might be because the metal co‐catalysts covered the OVs on the TiO_2_ surface, which were essential adsorption sites for the nitro‐oxygen atoms in nitrobenzene.^[^ ^195^ ^]^

Ta‐doped CeO_2_ (Ta‐CeO_2_) was used as a photocatalyst, with Ta doping altering the local structure of CeO_2_ and introducing a significant amount of Ce^3+^.^[^ ^196^ ^]^ This modification enhanced the generation of photogenerated carriers and increased the selectivity for C─C bond cleavage. The catalyst exhibited excellent photo‐thermal synergistic effects, facilitating the complete scission of glucose's C1─C2 bond through integrated light and thermal activation.^[^ ^196^ ^]^ Radical scavenging experiments and intermediate analysis revealed that photogenerated h^+^ initiated C─H bond oxidation in glucose. These radicals then reacted with O_2_, which was reduced by photogenerated eˉ, leading to C─C bond cleavage and the formation of HCOOH and formaldehyde. This remarkable performance was attributed to lattice distortion in CeO_2_ caused by Ta doping, which narrowed the bandgap (from 2.60 to 2.37 eV) and increased the concentration of Ce^3+^.^[^ ^196^ ^]^ These changes enhanced the catalyst's adsorption capacity for reactive intermediates, promoted the generation and separation of photogenerated carriers, and thus increased the selectivity for C─C bond cleavage.

To overcome the limitations of insufficient visible light absorption and low photogenerated carrier separation efficiency in Bi_2_WO_6_, CoPz was introduced.^[^ ^81^ ^]^ CoPz, with its strong visible light absorption capabilities, extended the light absorption range of Bi_2_WO_6_.^[^ ^81^ ^]^ However, CoPz, being a macrocyclic compound, tends to aggregate. To overcome this limitation, CoPz was integrated with Bi_2_WO_6_, forming a composite that exhibited markedly enhanced photocurrent density compared to the individual components. This improvement highlighted the role of CoPz in promoting more efficient separation of photogenerated charge carriers.^[^ ^81^ ^]^ Notably, the catalyst reached its optimal glucose conversion rate of 45.3% when the CoPz loading was set at 0.25%. However, increasing the CoPz content further (to 0.5% or 1%) led to a decrease in catalytic activity, which was attributed to CoPz aggregation on the Bi_2_WO_6_ surface, hindering the separation and transfer of photogenerated carriers.^[^ ^81^ ^]^

***Summary***. Research on photocatalytic glucose to HCOOH has focused on catalyst design, optimization of reaction conditions, and improving product selectivity (Table S7, Supporting Information). Notably, developing composite catalysts (such as Bi_2_O_3_/TiO_2_ and Ta‐CeO_2_) and the integration of bioenzymes with semiconductors have significantly enhanced photocatalytic efficiency and selectivity, enabling efficient transformations under mild reaction conditions. However, challenges persist in balancing HCOOH selectivity with glucose conversion and in fully understanding the underlying reaction mechanisms. Future research should aim to further optimize catalyst performance and gain deeper insights into the reaction mechanisms to enable more efficient photocatalytic production of HCOOH from glucose.

### 4.3 H_2_O_2_

**Figure 29:**
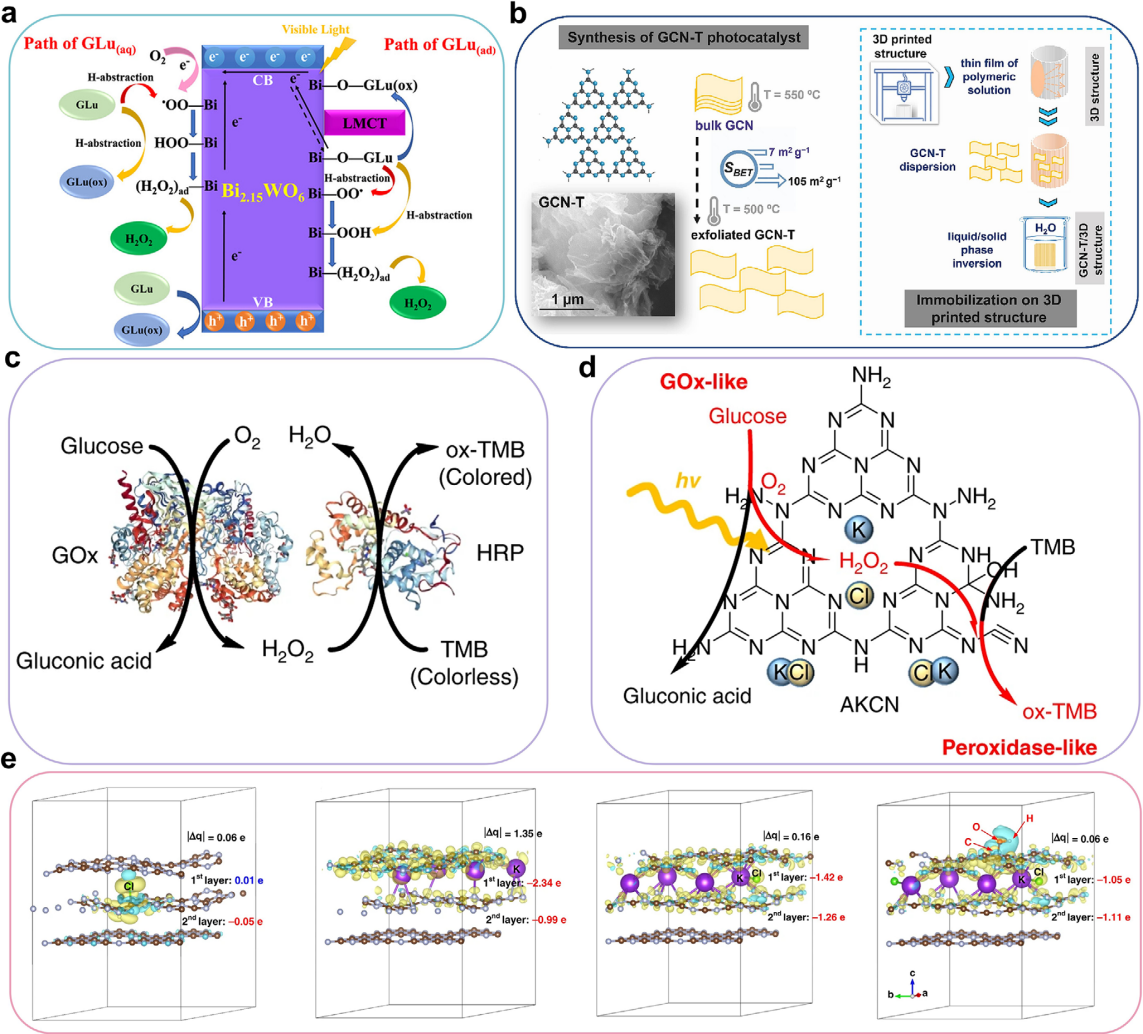
a) Mechanisms involved in the photocatalytic generation of H_2_O_2_ by Bi_2.15_WO_6_. Reproduced with permission.^[^ ^198^ ^]^ Copyright 2022, American Chemical Society. b) Schematic procedure of synthesis and immobilization of the photocatalyst. Reproduced with permission.^[^ ^199^ ^]^ Copyright 2023, Elsevier. c) Colorimetric detection diagram of glucose using GOx and HRP. d) Photocatalytic aerobic oxidation of glucose with in situ production of H_2_O_2_ on AKCN. e) Charge distribution of Cl‐GCN, K‐GCN, KCl‐GCN, and KCl‐OH‐GCN. Reproduced with permission.^[^ ^200^ ^]^ Copyright 2019, Springer Nature.

Traditional methods for producing H_2_O_2_, such as the anthraquinone process, suffer from high energy consumption and the use of organic solvents.^[^ ^197^ ^]^ Utilizing glucose as a sacrificial agent provides a sustainable pathway for H_2_O_2_ production (**Figure** **29**a). In this process, the ─OH of glucose forms LMCT complexes with Bi(III) on the surface of Bi_2.15_WO_6_, significantly enhancing the photoelectrochemical performance of Bi_2.15_WO_6_.^[^ ^198^ ^]^ Reduced charge recombination and improved separation efficiency of photogenerated carriers characterized this enhancement. DFT calculations revealed that oxygen activation on the Bi_2.15_WO_6_ surface involved low energy barriers for hydrogen atom transfer from glucose to the •Bi−OO• and •Bi−OOH species, indicating that glucose acted as an effective hydrogen atom donor.^[^ ^198^ ^]^ Photocatalytic materials exhibiting high stability in both ultrapure water and seawater were obtained by immobilizing exfoliated graphitic carbon nitride materials (GCN‐T) on 3D structures (GCN‐T/3D) (Figure 29b).^[^ ^199^ ^]^ Notably, the efficiency of photocatalytic H_2_O_2_ generation was positively correlated with the number of ─OH in the molecular structure.^[^ ^199^ ^]^ This correlation arises because the ─OH groups in sugars served as efficient eˉ donors, which promoted photogenerated h^+^ capture and subsequently improved H_2_O_2_ generation efficiency.^[^ ^199^ ^]^

Although exhibiting exceptional substrate selectivity and reaction efficiency, natural enzymes were plagued by high production expenses and weak stability. Zhang et al. used modified g‐C_3_N_4_ (GCN for short) as a metal‐free bifunctional nano‐enzyme. An AKCN catalyst bifunctional nano‐enzyme was developed by introducing KOH and KCl to modify GCN.^[^ ^200^ ^]^ The nanoparticles were able to mimic the functions of glucose oxidase (GOx) and horseradish peroxidase (HRP) simultaneously to achieve efficient gluconic acid production through glucose oxidation and to promote H_2_O_2_ production (Figure 29c,d).^[^ ^200^ ^]^ Superior catalytic activity resulted from the compensatory effect of Cl on K‐generated electron density redistribution within the carbon nitride matrix. Charge migration between layers occurred more readily in KCl‐modified carbon nitride than in pure GCN (Figure 29e).^[^ ^200^ ^]^

### 4.4 Syngas

**Figure 30:**
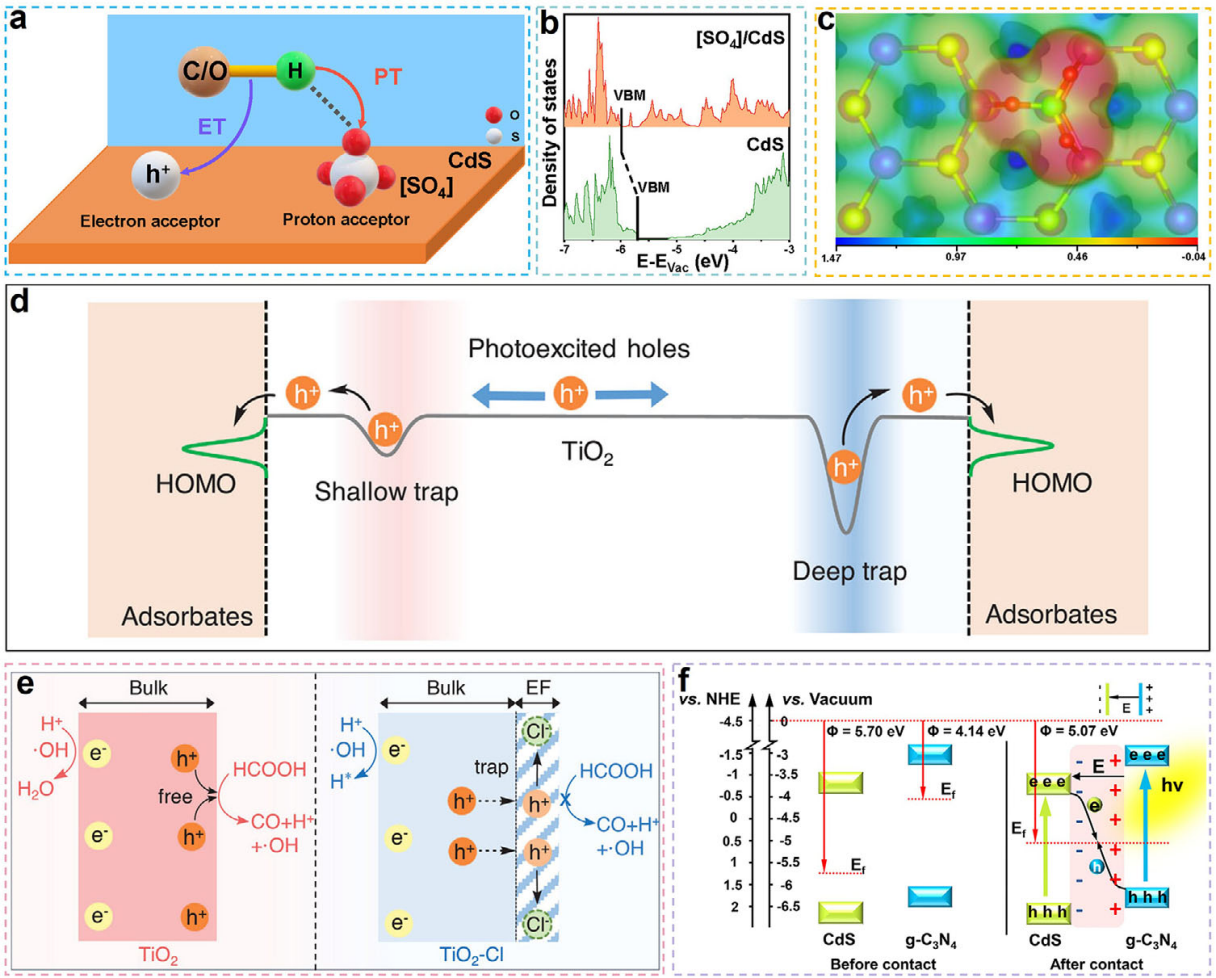
a) Proposed electron‐proton transfer mechanism in photoexcited [SO_4_]/CdS system. b) Total DOS of CdS(001) and [SO_4_]/CdS(001) slab. c) Electron density isosurface of [SO_4_]/CdS(001). Reproduced with permission.^[^ ^15^ ^]^ Copyright 2021, American Chemical Society. d) Concept of electric field‐mediated hole trap state‐controlled hole transfer process. e) Scheme of photocatalytic HCOOH decomposition over TiO_2_ and TiO_2_‐Cl. Reproduced with permission.^[^ ^202^ ^]^ Copyright 2024, Wiley. f) Schematic mechanism of Z‐scheme structure at CdS/g‐C_3_N_4_ interface. Reproduced with permission.^[^ ^203^ ^]^ Copyright 2022, Cell.

Syngas, primarily composed of CO and H_2_, is a crucial feedstock in the chemical industry and is typically produced via the high‐temperature gasification of biomass (400–700 °C).^[^ ^201^ ^]^ However, this process is highly energy‐intensive. Photocatalytic glucose conversion emerges as an ideal alternative for syngas production, as it leverages biomass carbon and solar energy and can be conducted at room temperature. The introduction of [SO_4_]_2_ˉ on the surface of CdS catalysts has been shown to significantly enhance the efficiency of eˉ and proton transfer (**Figure** **30**a).^[^ ^15^ ^]^ During the photocatalytic process, [SO_4_]_2_ˉ functioned as both a proton acceptor and an eˉ transfer promoter. It interacted with hydrogen atoms in biomass through hydrogen bonds, facilitating proton transfer.^[^ ^15^ ^]^ Such coupling improved the oxidative power of the VB while simultaneously accelerating electron transport (Figure 30b,c).

A catalyst consisting of Cu dispersed on defect‐rich titanium dioxide nanorods (Cu/TNR) enabled the selective cleavage of glucose C─C bonds to produce methanol and syngas by modulating its band structure.^[^ ^204^ ^]^ Notably, by adjusting the Cu loading and water concentration, the CO/CO_2_ ratio could be precisely controlled, achieving up to 90% CO selectivity. The TiO_2_ nanorods in Cu/TNR were rich in OVs, which played a crucial role in the adsorption and activation of polyols. DFT calculations revealed that these OVs significantly lower the activation energy required for C─C bond cleavage.^[^ ^204^ ^]^ Additionally, the selective regulation of glucose photo‐reforming could be achieved by introducing an electrostatic field through the surface modification of TiO_2_ with anion adsorbates (e.g., Clˉ).^[^ ^202^ ^]^ This modification altered the trapping states of photogenerated h^+^, thereby influencing interfacial h^+^ transfer efficiency and steering the reaction pathway toward either HCOOH or CO. Interestingly, on unmodified TiO_2_, photogenerated h^+^ occupy shallow trap states, facilitating their transfer and promoting the oxidation of FA's C–H bonds to generate CO (Figure 30d). In contrast, on TiO_2_‐Cl, the photogenerated h^+^ was confined in deep trap states, hindering their transfer and stabilizing HCOOH (Figure 30e).^[^ ^202^ ^]^ Furthermore, Zhou et al. developed an O‐controlled photocatalytic conversion strategy utilizing a Z‐scheme heterojunction and a core‐shell CdS@g‐C_3_N_4_ catalyst to efficiently generate CO from glucose under mild conditions (Figure 30f).^[^ ^203^ ^]^ This core‐shell CdS@g‐C_3_N_4_ catalyst effectively adsorbed and activated oxygen, facilitating the formation of •OH, which accelerated the oxidation of biomass.

***Summary***. Overall, glucose is used not only for the photocatalytic production of important platform molecules such as H_2_, sugar acids, lactic acid, HMF, HCOOH, and arabinose but also for the photocatalytic transformation into other high‐value small molecules, including H_2_O_2_ and syngas (Table S8, Supporting Information). These processes demonstrate significant potential for the efficient valorization of glucose, promising to serve as sustainable alternatives to fossil feedstocks. Developing more advanced photocatalytic strategies is thus imperative for optimizing glucose utilization.

## 5 Conclusion and Perspectives

With the increasing demand for sustainable energy solutions and environmental protection, the development of renewable energy sources as viable alternatives to fossil fuels has become a global research focus. In this context, the photocatalytic oxidation of glucose has garnered growing attention owing to its renewable nature and potential for high‐value chemical transformations, underscoring its critical research significance. In recent years, substantial progress has been made in glucose photocatalysis, enabling its selective conversion into bio‐fuels and a variety of valuable chemical products, H_2_, gluconic acid, arabinose, HCOOH, HMF, and lactic acid. Additionally, researchers have explored the feasibility of photocatalytic glucose conversion for synthesizing H_2_O_2_, amino acids, syngas, and glycerol, further broadening the scope of this field's applications. Notably, among various photocatalytic conversion routes of glucose, the oxidation to gluconic acid, photo‐reforming to H_2_, dehydration to HMF, and C─C bond cleavage to organic acids demonstrate particularly high promise. This significance stems from two key aspects: First, these products find extensive applications in food, cosmetics, pharmaceutical, and chemical industries, exhibiting substantial market demand. Second, these reaction pathways have been extensively investigated and are poised to drive the transition from petroleum refining to biorefining as a cornerstone of the carbon‐neutral economy. However, research on glucose photo‐reforming to H_2_O_2_, amino acids, syngas, and glycerol remains at a preliminary stage.

This review systematically summarizes the reaction mechanisms and catalyst design strategies for glucose photocatalytic oxidation, with a particular emphasis on how different catalytic systems influence product selectivity and conversion efficiency. It has been demonstrated that rationally tuning catalyst structures and surface properties could significantly enhance both selectivity and catalytic performance. Specifically, various catalyst optimization strategies such as morphology control, doping, defect engineering, Schottky junction formation, and heterojunction construction have been extensively studied and proven effective. These approaches improved glucose photocatalytic conversion by reducing bandgap energy, enhancing light absorption, increasing charge carrier separation efficiency, and prolonging carrier lifetimes. However, several challenges persisted in glucose photo‐reforming. Significant side reactions often led to the formation of undesired byproducts, thereby limiting selectivity. Photocatalysts typically utilize only the UV to visible light spectrum, resulting in underutilization of infrared radiation. Moreover, high‐performance photocatalysts frequently rely on noble metals, which increases overall costs. The long‐term stability and recyclability of photocatalysts under operational conditions require further improvement to ensure practical feasibility. Additionally, precise modulation of active sites and reaction pathways remained a major challenge for achieving high selectivity in the conversion of glucose to target products. Furthermore, while significant advancements have been made at the laboratory scale, key hurdles related to scalability, cost‐effectiveness, and reactor engineering must be addressed for future industrial implementation.

**Figure 31:**
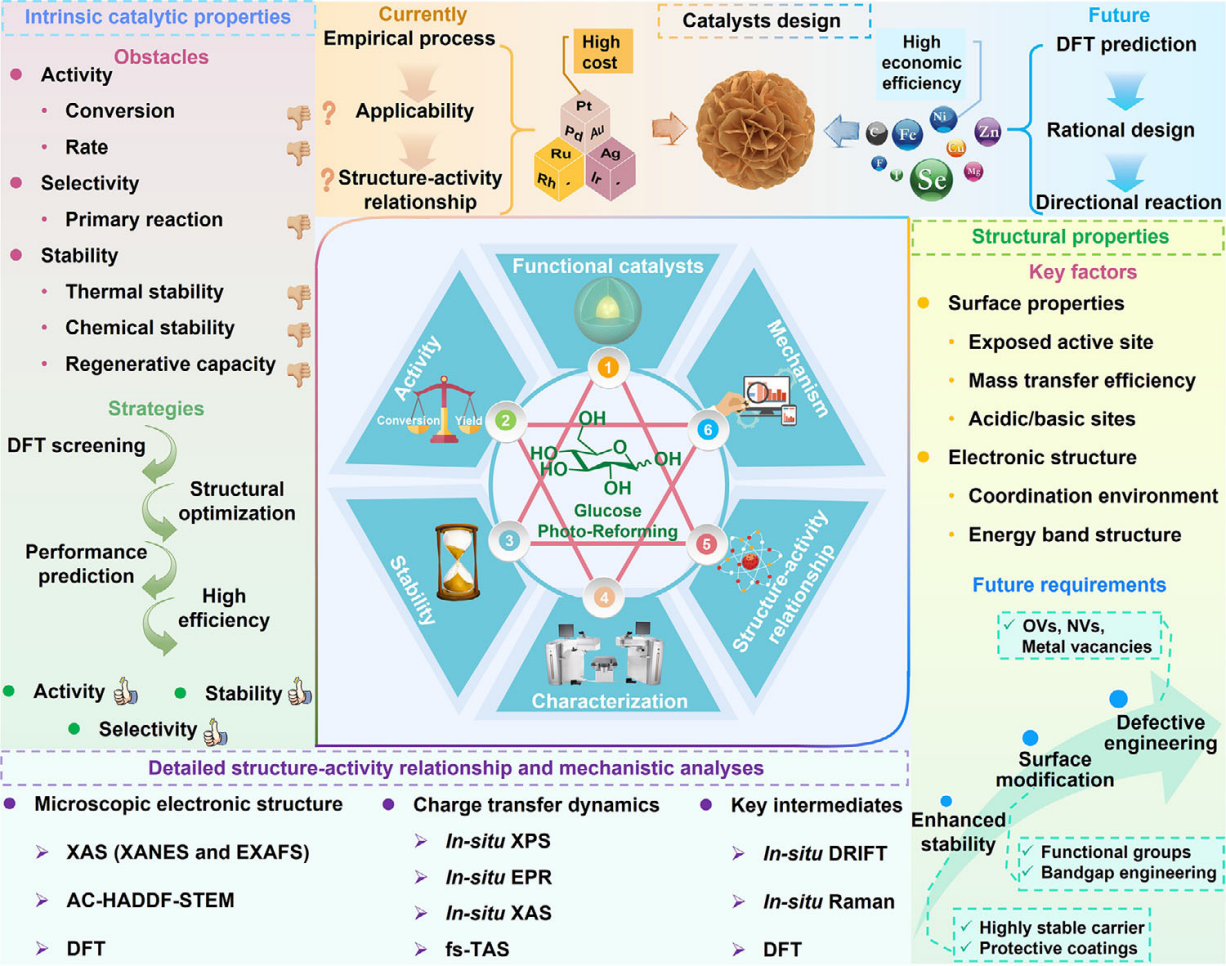
Current challenges and prospects of photo‐reforming glucose.

In the field of photocatalytic glucose conversion, future research directions will primarily focus on rational catalyst design, stability enhancement, cost reduction, expansion of the light response range, optimization of catalytic efficiency, in‐depth mechanistic studies, and the scale‐up process for glucose photo‐reforming, all aimed at facilitating the practical application of this technology (**Figure** **31**).

- Rational Catalyst Design

DFT calculations have become an essential tool for screening and predicting highly efficient photocatalysts, enabling the evaluation of electronic structures, adsorption energies, and reaction energy barriers of various materials to guide experimental optimization.^[^ ^205^, ^206^ ^]^ Future studies may integrate high‐throughput computing and machine learning methods to explore materials with superior photocatalytic performance. Additionally, strategies involving multi‐component synergistic catalysis, such as the construction of multi‐metal oxides, co‐doped systems, or 2D‐3D composite structures, hold great promise for electronic and surface properties in photocatalysts.

- Enhancing Catalyst Stability

Future research should focus on utilizing highly stable supports, including graphene oxide (GO) and Aurivillius compounds, to minimize photo‐corrosion and structural degradation. Moreover, protective coatings (e.g., carbon layers, SiO_2_, or Al_2_O_3_) and surface modifications (e.g., passivation layers, functional group modifications) can effectively mitigate catalyst deactivation during prolonged reactions. Additionally, optimizing catalyst preparation methods, such as sol–gel synthesis, hydrothermal methods, and plasma treatment, can further enhance material stability.

- Reducing Catalyst Costs

Developing efficient catalytic materials at reduced costs emerges as an important research priority moving forward. This can be achieved by substituting noble metals with non‐noble metals (e.g., Fe, Co, Ni, Cu) or by synthesizing metal‐organic frameworks (MOFs) and covalent organic frameworks (COFs) to construct highly efficient catalytic materials. Additionally, incorporating biochar and other inexpensive, renewable resources as catalyst supports can not only reduce costs but also enhance the conductivity and stability of the catalysts.

- Expanding the Light Response Range

The construction of S‐scheme and Z‐scheme heterojunctions can effectively improve charge separation efficiency and expand the light response range. Furthermore, bandgap engineering strategies, such as doping and energy band modulation, can enhance light absorption properties. For example, non‐metal doping (e.g., N, S, P, B) or local structural adjustments can tailor the bandgap of catalysts, thereby improving their absorption in the visible light region.

- Improving Catalytic Efficiency

Tailoring the structural and electronic properties of catalysts enhances their reactivity with critical reaction intermediates. For example, surface functionalization with ─OH, ─COOH, and ─NH_2_ groups can introduce abundant acidic/basic sites, enhancing the adsorption of glucose and its derivatives. Additionally, defect engineering (e.g., OVs, NVs, metal vacancies) can provide extra active sites, thereby promoting redox reactions.

- In‐depth Mechanistic Studies

It is essential to gain an in‐depth understanding of the microscopic electronic structure of catalysts, charge carrier transfer dynamics, and key intermediates. Future studies should leverage advanced characterization techniques to monitor structural evolution, electron transfer pathways, and adsorption‐desorption behaviors in real time. Additionally, combining theoretical calculations with experimental data can help establish a comprehensive reaction pathway model, providing theoretical guidance for catalyst optimization and mechanistic understanding.

- Developing Industrial Applications

Scaling photocatalytic glucose conversion to high‐value molecules from laboratory to industrial settings represents a critical goal, yet its practical feasibility remains daunting. This challenge primarily stems from limitations in reactor design and mass transfer. Future research should focus on optimizing reactor design and reaction conditions to achieve efficient glucose conversion in larger volumes. This includes enhancing the photocatalyst's stability and light absorption efficiency under industrial‐scale conditions, as well as developing cost‐effective methods for catalyst recovery and recycling.

In summary, with the ongoing development of new materials, methodologies, and theoretical frameworks, photocatalytic glucose conversion technology is expected to achieve higher efficiency, lower costs, and industrial‐scale applications, thereby playing a crucial role in renewable energy utilization and green chemical synthesis.

## Conflict of Interest

The authors declare no conflict of interest.

## Supporting information



Supporting Information
