# Supplementary material for: Rational Design of Advanced Functional Catalysts for Photo‐Reforming Glucose into Bio‐Derived Fuels and Chemicals
Source: Adv Sci (Weinh). 2025 Jul 13;12(34):e08850. doi: 10.1002/advs.202508850 (PMC12442708; doi:10.1002/advs.202508850)
Supplement: Supplementary file 1 — Supporting Information [file ADVS-12-e08850-s001.docx]

***Supplementary Material***

**Rational Design of** **Advanced Functional Catalysts for Photo-Reforming Glucose into Bio-Derived Fuels and Chemicals**

*Qiong Yan, Yuyue Zhou, Yan Zhang, Dalin Sun, Xu Wu, Song Yang*, Chunbao Xu*, Heng Zhang**

Q. Yan, Y. Zhou, Y. Zhang, D. Sun, X. Wu, S. Yang, H. Zhang

State Key Laboratory of Green Pesticide, State-Local Joint Laboratory for Comprehensive Utilization of Biomass, Center for R&D of Fine Chemicals of Guizhou University, Guiyang, 550025, China

E-mail: [syang@gzu.edu.cn](mailto:syang@gzu.edu.cn); [hzhang23@gzu.edu.cn](mailto:hzhang23@gzu.edu.cn)

C. Xu

*School of Energy and Environment, City University of Hong Kong, Kowloon Tong, Hong Kong*

E-mail: [chunbaxu@cityu.edu.hk](mailto:chunbaxu@cityu.edu.hk)

**This document includes tables and references, distributed as follows:**

**Table S1:** Performances of various photocatalysts in conversion of glucose into gluconic acid **Page S3**

**Table S2:** Performance of various photocatalysts in conversion of glucose to HMF **Page S4**

**Table S3:** Overview of the performance of various photocatalysts in preparation of arabinose from glucose **Page S5**

**Table S4:** Summary of performance of various photocatalysts in preparation of lactic acid from glucose **Page S6**

**Table S5:** Summary of performance of various photocatalysts in preparation of glycerol and amino acids from glucose **Page S7**

**Table S6:** Performances of various photocatalysts in H_2_ evolution **Pages S8-10**

**Table S7:** Overview of the performance of various photocatalysts in preparation of HCOOH from glucose **Page S11**

**Table S8:** Summary of performance of various photocatalysts in preparation of H_2_O_2_ and syngas from glucose **Page S12**

**References** **Pages S13-16**

| **Table S1.** Performance of various photocatalysts in conversion of glucose into gluconic acid. | | | | | | |
| --- | --- | --- | --- | --- | --- | --- |
| Entry | Catalyst | Light | Reaction time  (h) | Conversion  (%) | Selectivity  (%) | Ref. |
|  | Fe_1_CN-20  PMS | Xe lamp | 0.5 | 36.3 | 91.6 | [1] |
|  | RCN | Xe lamp | 4 | 60.0 | 60.0 | [2] |
|  | Ce6@BNCN  H_2_O_2_ | Xe lamp | 2 | 62.3 | 70.9 | [3] |
|  | g-C_3_N_4_/CoPz  H_2_O_2_ | Xe lamp | 0.3 | 52.1 | 79.4 | [4] |
|  | CNK-OH  TEMPO | Xe lamp | 3 | 30.0 | 100.0 | [5] |
|  | CNKS-OH | Xe lamp | 6 | 80.0 | 70.0 | [6] |
|  | AKCN | Xe lamp | 8 | 75.0 | 75.0 | [7] |
|  | TiO_2_ | Xe lamp | 4 | 42.0 | 7.0 | [8] |
|  | CTAB/MW-TiO_2_ | Hg lamp  (λ = 365 nm) | 2 | 62.3 | 12.5 | [9] |
|  | US/CTAB-TiO_2_ | Hg lamp  (λ = 365 nm) | 2 | 69.5 | 10.9 | [10] |
|  | TiO_2_/ZeY | Hg lamp  (λ = 365 nm) | 2 | 75.0 | 12.0 | [11] |
|  | TiO_2_(US) | Hg lamp  (λ = 365 nm) | 0.17 | 11.0 | 71.3 | [12] |
|  | HPA/TiO_2_ | Hg lamp | 6 | 53.0 | 19.0 | [13] |
|  | TiO_2_/HPW/CoPz | Xe lamp | 3 | 22.2 | 80.4 | [14] |
|  | Ag-TiO_2_ | Hg lamp | 2 | 68.6 | 23.6 | [15] |
|  | Ag/N-doped TiO_2_ | Hg lamp (λ = 250-365 nm) | 3 | 97.7 | 12.6 | [16] |
|  | ZnO | UV- light (λ = 365 nm) | 3 | 21.5 | 15.0 | [17] |
|  | ZnO/CoPzS_8_ | Xe lamp | 5 | 75.0 | - | [18] |
|  | SnO_2_/FePz(SBu)_8_ | Xe lamp | 7 | 34.2 | 45.8 | [19] |
|  | SnO_2_-OVs/CoPz | Xe lamp | 3 | 43.6 | 60.7 | [20] |
|  | H-ZSM-5/FePz(SBu)_8_ H_2_O_2_ | Xe lamp (λ > 420 nm) | 4 | 35.8 | 45.0 | [21] |
|  | M-CdS/MOR | UV lamps system (λ = 254 nm) | 1 | 36.0 | - | [22] |
|  | Au/CeO_2_  H_2_O_2_ | A.M.1.5G | 0.16 | 99.0 | 95.0 | [23] |

| **Table S2.** Performance of various photocatalysts in conversion of glucose to HMF. | | | | | |
| --- | --- | --- | --- | --- | --- |
| Entry | Catalyst | Light | Reaction time  (h) | Yield  (%) | Ref. |
|  | FA_NPC* | 340-850 nm | 1 | 38.4 | [24] |
|  | AgFe_2_O_4_/TiO_2_-SO_3_H | LED | 1.2 | 64.0 | [25] |
|  | Ag/G-Al_2_O_3_-Cr^3+^ | Halogen lamp (λ = 400-750 nm) | 20 | 68.0 | [26] |
|  | G-A-Ga^3+^ | Halogen lamp (λ = 400-800 nm) | 4 | 60.8 | [27] |
|  | FS-Al^3+^ | Halogen lamp (λ = 400-800 nm) | 20 | 60.0 | [28] |
|  | TiO_2_/g‑C_3_N_4_/SO_3_H(IL) | LED | 1 | 96.0 | [29] |

| **Table S3.** Overview of the performance of various photocatalysts in preparation of arabinose from glucose. | | | | | | |
| --- | --- | --- | --- | --- | --- | --- |
| Entry | Catalyst | Light | Reaction time  (h) | Conversion  (%) | Selectivity  (%) | Ref. |
|  | AuCN | Xe lamp | 6 | 30.0 | 40.0 | [30] |
|  | FA_NPC* | Xe lamp | 1 | 91.0 | 30.0 | [31] |
|  | NiTiO_3_ | Xe lamp | 4 | 9.0 | 75.0 | [32] |
|  | Pt/TiO_2_-R | Xe lamp | 5 | 65.0 | 67.0 | [33] |
|  | 3DOM TiO_2_-Au | Xe lamp (λ = 420-780 nm) | 8 | 37.0 | 27.0 | [34] |
|  | Pd/Meso CdS | Xe lamp (λ > 420 nm) | 1 | 20.0 | 70.0 | [35] |
|  | OCN-OH | Xe lamp AM 1.5 | 6 | 10.0 | 90.0 | [36] |

| **Table S4.** Summary of performance of various photocatalysts in preparation of lactic acid from glucose. | | | | | | |
| --- | --- | --- | --- | --- | --- | --- |
| Entry | Catalyst | Light | Reaction time  (h) | Conversion  (%) | Selectivity  (%) | Ref. |
|  | B@mCN-3 | Visible light | 1.5 | 99.0 | 77.8 | [37] |
|  | HC-CN | Xe lamp | 1 | 99.8 | 94.5 | [38] |
|  | RPCN | Xe lamp | 1 | 98.5 | 90.0 | [39] |
|  | Mg-CN/CS | Xe lamp | 2 | 97.0 | 71.0 | [40] |
|  | OCN/5%artCS | Xe lamp | 1 | 99.0 | 90.0 | [41] |
|  | NTCN/LDH | LED (λ= 400-780 nm) | 1 | 99.9 | 92.0 | [42] |
|  | Zn-mCN | Visible light | 6 | - | 43.2 | [43] |
|  | Pt_NP_-C_3_N_4_ | LED (λ = 427 nm) | 4 | 100.0 | 86.0 | [44] |
|  | HCN | Xe lamp (λ > 420 nm) | 1.5 | - | 55.5 | [45] |
|  | PVA@CNNS | Xe lamp | 2 | 100.0 | 92.6 | [46] |
|  | CT-700 | Xe lamp (λ > 380 nm) | 2 | 62.0 | 51.6 | [47] |
|  | s-TCS-4 | Xe lamp | 5 | 95.9 | 94.3 | [48] |
|  | CuO@CS-H | Vis-light (λ ＞ 420 nm) | 1 | 98.2 | 55.3 | [49] |
|  | Cu/Cu_2_O/CuO@CA | Vis-light (λ ＞ 420 nm) | 0.4 | 99.0 | 67.1 | [50] |
|  | Zn_0.1_Cd_0.9_S/NiS | Xe lamp (λ > 420 nm) | 5 | 95.0 | 49.9 | [51] |
|  | Zn_0.6_Cd_0.4_S | Xe lamp (λ > 420 nm) | 5 | 90.0 | 87.0 | [52] |
|  | CNT/LDHs | Xe lamp (λ = 320-780 nm) | 1 | 98.4 | 90.0 | [53] |
|  | CQDs@4CzIPN | Vis-light (λ > 420 nm) | 0.5 | 99.8 | 71.1 | [54] |

| **Table S5.** Summary of performance of various photocatalysts in preparation of glycerol and amino acids from glucose. | | | | | |
| --- | --- | --- | --- | --- | --- |
| Entry | Catalyst | Light | Reaction Time (h) | Yield | Ref. |
|  | BCM-CN | Xe lamp | 3 | 47.5% | [55] |
|  | CdS/Ti_3_C_2_ | Xe lamp (λ > 420 nm) | 8 | 1.0 mmol g^-1^ h^-1^ | [56] |
|  | CdS | Xe lamp (λ = 420-780 nm) | 8 | 0.34 mmol g^-1^ h^-1^ | [57] |
|  | Ba^2+^-TiO_2_ | LED (λ = 365 nm) | 10 | 15.0 μmol | [58] |

| **Table S6.** Performances of various photocatalysts in H_2_ evolution. | | | | | | | |
| --- | --- | --- | --- | --- | --- | --- | --- |
| Entry | Catalyst | Co-catalyst | pH | Light | Reaction time  (h) | Hydrogen evolution rate (μmol g^-1^ h^-1^) | Ref. |
|  | MTCA-100-CN | H_2_PtCl_6_ | 11 | Xe lamp (AM1.5G) | 4 | 127.6 | [59] |
|  | o-g-C_3_N_4_ | Pt | 11 | Xe lamp | 6 | 870.0 | [60] |
|  | g-C_3_N_4__0.2Pd | - | 10 | Xe lamp (λ = 100-1800 nm) | 4 | 1839.8 | [61] |
|  | Pt-MCNN-3.0% | - | - | Xe lamp (λ = 100-1800 nm) | 3 | 201.2 | [62] |
|  | Pt_0.5_Au_1.5_/CN | - | 13 | Xe lamp (λ = 350-800 nm) | 3 | 904.0 | [63] |
|  | DMASnBr_3_/g-C_3_N_4_ | H_2_PtCl_6_ | - | Solar Box 1500e | 6 | 925.0 | [64] |
|  | (Dy_2_O_3_/ND-g-C_3_N_4_) | - | - | Xe lamp (λ > 420 nm) | 4 | 510.3 | [65] |
|  | Cd_0.8_Zn_0.2_S/Au/g-C_3_N_4_ | - | - | Xe lamp  (λ > 420 nm) | 10 | 123.0 | [66] |
|  | Pt/PRCN | - | 7 | Xe lamp  (λ > 400 nm) | 2 | 72.3 | [67] |
|  | THMs | H_2_PtCl_6_ | - | Xe lamp  (λ = 200 - 800 nm) | 2 | 9440.0 | [68] |
|  | TiO_2_  -Au-CdS | - | 1 | Xe lamp  (λ = 300 - 1100 nm) | 8 | 4648.0 | [69] |
|  | Pt/(CNT-TiO_2_)ox-473 | - | 7 | Heraeus TQ 150  (λ = 365 nm) | 2 | 99.1 | [70] |
|  | Pd/TiO_2_ | - | 11 | Hg lamp | 14 | 4580.0 | [71] |
|  | Pt/TiO_2_ | - | 12 | Hg lamp  (λ = 250 - 400 nm) | 3 | 153.0 | [72] |
|  | Pd/TiO_2_ | - | 7 | Xe lamp  (λ = 350 nm) | 12 | - | [73] |
|  | PtO_x_/TiO_2_ | - | 7 | LED  (λ = 380 nm) | 1.7 | 1650.0 | [74] |
|  | Pt/TiO_2_ | - | - | Hg lamp  (λ = 254 nm) | 6 | - | [75] |
|  | P25 | H_2_PtCl_6_ | 5.5 | Hg lamp  (λ = 254 - 254 nm) | 5 | 14690.0 | [76] |
|  | Pt-F-TiO_2_ | - | 2 | LED  (λ = 375-380 nm) | 3 | 590.0 | [77] |
|  | Pt/TiO_2_-Nb_2_O_5_ | - | - | LED  (λ = 254 nm) | 1 | 79.2 | [78] |
|  | Ni_0.05_Au_0.45_/TiO_2_ | - | - | Xe lamp  (λ = 200-2500 nm) | 4 | 6391.9 | [79] |
|  | Er^3+^:YAlO_3_/Pt-TiO_2_ | - | 6.5 | Xe lamp  (λ = 400-700 nm) | 5 | 153.9 | [80] |
|  | CQDs/TiO_2_ | - | - | Xe lamp  (λ = 320-780 nm) | 5 | 2430 | [81] |
|  | h-ZnSe/Pt@TiO_2_ | Pt | - | Xe lamp | 140 | 157.0 | [82] |
|  | CdS/TiO_2_ | H_2_PtCl_6_ | - | LED  (λ = 365 nm) | 2 | 2760 | [83] |
|  | Zn_0.3_Cd_0.7_S | - | 12 | Xe lamp | 4 | 13640.0 | [84] |
|  | Au/CdS-NRs | - | 7 | Xe lamp  (λ > 400 nm) | 4 | 90.0 | [85] |
|  | CdS/MoS_2_ | - | 7 | Xe lamp  (λ > 400 nm) | 3 | 55000.0 | [86] |
|  | Pt/Cd_x_Zn_1-x_S | - | 12 | Hg lamp  (λ > 420 nm) | 2 | 301.3 | [87] |
|  | Pt/Cd_0.5_Zn_0.5_S | - | 12 | Hg lamp  (λ > 420 nm) | 3 | - | [88] |
|  | 1%Pt/Cd_0.6_Zn_0.4_S/Cd_0.1_Zn_0.9_S | - | - | LED  (λ = 450 nm) | 3 | 3400.0 | [89] |
|  | ZnS-ZnIn_2_S_4_ | H_2_PtCl_6_ | 12.65 | Hg lamp  (λ > 420 nm) | 10 | 103000.0 | [90] |
|  | NiS/Cd_0.5_Mn_0.5_S | 0.1 M Na_2_S/_0.1_ M Na_2_SO_3_ | - | LED  (λ = 425 nm) | 1.5 | 7300.0 | [91] |
|  | CoP@MoS_2-x_ | - | - | LED | 4 | 1127.4 | [92] |
|  | Ru-LaFeO_3_ | - | - | LED  (λ = 440 nm) | 4 | 910000.0 | [93] |
|  | LaFeO_3_ | - | 7 | LED  (λ = 375-380 nm) | 4 | 400.0 | [94] |
|  | LaFeO_3_ | - | 6 | Philips TL  (λ = 365 nm) | 4 | 429.0 | [95] |
|  | Ru-LaFeO3 | - | 6 | LED  (λ = 375-380 nm) | 3 | 1158.0 | [96] |
|  | Bi_0.5_Y_0.5_VO_4_ | H_2_PtCl_6_ | 3 | Xe lamp  (λ > 430 nm) | 2 | 7.5 | [97] |
|  | Bi_2_WO_6_ | - | 7 | Xe lamp  (λ = 290-900 nm) | 2.5 | 3050.0 | [98] |
|  | 3% CuBi_2_O_4_-P25 | - | - | Hg lamp  (λ = 400-700 nm) | 5 | 693.3 | [99] |
|  | In_2_O_3_-MR | H_2_PtCl_6_ | 11 | Xe lamp | 5 | 688800.0 | [100] |
|  | Ni-InVO_4_ | - | - | Hg lamp | 11 | 116.7 | [101] |
|  | NaTaO_3_ | - | - | Hg lamp | 11 | 11272.7 | [102] |
|  | HCN_3_×BuNH_2_ | H_2_PtCl_6_ | - | Hg lamp  (λ = 365 nm) | 2 | 15000.0 | [103] |
|  | 3DOM CTO-ZCS-C | - | - | Xe lamp  (λ = 365 nm) | 1 | 2810.0 | [104] |
|  | CTFs | H_2_PtCl_6_ | 12 | Xe lamp  (λ > 420 nm) | 2 | 330000.0 | [105] |
|  | SNGODs | H_2_PtCl_6_ | 10 | Xe lamp  (λ = 420-800 nm) | 12 | 164.0 | [106] |

| **Table S7.** Overview of the performance of various photocatalysts in preparation of HCOOH from glucose. | | | | | | |
| --- | --- | --- | --- | --- | --- | --- |
| Entry | Catalyst | Light | Reaction time  (h) | Conversion  (%) | Selectivity  (%) | Ref. |
|  | TiO_2_ | Hg lamp | 3 | 100.0 | 35.0 | [107] |
|  | Bi_2_O_3_/TiO_2_ | Xe lamp (λ ＞ 420 nm) | 4 | 65.7 | 25.6 | [108] |
|  | CdS/TiO_2_/BC | Xe lamp (λ > 420 nm) | 3 | 95.0 | 60.3 | [109] |
|  | TiO_2_/jFDH | AM 1.5G | 24 | - | - | [110] |
|  | TiO_2_ | Hg lamp (λ > 300 nm) | 3 | - | - | [111] |
|  | Ta-CeO_2_ | Sun light | 15.5 | 19.0 | 70.0 | [112] |
|  | Bi_2_WO_6_/CoPz | Xe lamp (λ ＞ 420 nm) | 3 | 45.3 | 60.6 | [113] |

| **Table S8.** Summary of performance of various photocatalysts in preparation of H_2_O_2_ and syngas from glucose. | | | | | |
| --- | --- | --- | --- | --- | --- |
| Entry | Catalyst | Light | Reaction Time (h) | Yield | Ref. |
|  | Bi_2.15_WO_6_ | Visible light | 1 | 800.5 μmol | [114] |
|  | GCN-T/3D | LED (λ = 412 nm) | 6 | 0.25 μM | [115] |
|  | AKCN | λ > 420 nm | 1.5 | 100 μM | [116] |
|  | [SO_4_]/CdS | LED (λ = 455 nm) | 150 | (0.3 mmol g^-1^ h^-1^)  H_2_ (0.05 mmol g^-1^ h^-1^) | [117] |
|  | TiO_2_ | LED (λ = 365 nm) | 48 | 10.0% | [118] |
|  | CdS@g-C_3_N_4_ | LED (λ = 455 nm) | 24 | 32.0% | [119] |
|  | Cu/TNR | LED (λ = 365 nm) | 12 | 53.0% | [120] |

**References**

[1] T. Xia, M. Ju, H. Qian, X. Bai, R. Lai, C. Xie, G. Yu, Y. Tang, C. Wang, Q. Hou, *J. Catal.* **2024**, 429, 115257.

[2] J. Wang, L. Chen, H. Zhao, P. Kumar, S. R. Larter, M. G. Kibria, J. Hu, *ACS Catal.* **2023**, 13, 2637-2646.

[3] X. Bai, Q. Hou, H. Qian, Y. Nie, T. Xia, R. Lai, G. Yu, M. Laiq Ur Rehman, H. Xie, M. Ju, *Appl. Catal., B* **2022**, 303, 120895.

[4] Q. Zhang, X. Xiang, Y. Ge, C. Yang, B. Zhang, K. Deng, *J. Catal.* **2020**, 388, 11-19.

[5] J. Wang, Q. Zhao, Z. Li, Y. Xiao, X. Zhang, N. Zhong, H. Zhao, L. Jing, D. Di Tommaso, R. Crespo-Otero, M. G. Kibria, J. Hu, *Appl. Catal., B* **2025**, 360, 124526.

[6] J. Wang, H. Zhao, L. Chen, J. Björk, J. Rosen, P. Kumar, L. Jing, J. Chen, M. G. Kibria, J. Hu, *Appl. Catal., B* **2024**, 344, 123665.

[7] X. Liang, J. Liu, D. Zeng, C. Li, S. Chen, H. Li, *Electrochim. Acta* **2016**, 198, 40-48.

[8] L. Da Vià, C. Recchi, E. O. Gonzalez-Yañez, T. E. Davies, J. A. Lopez-Sanchez, *Appl. Catal., B* **2017**, 202, 281-288.

[9] J. Payormhorm, S. Chuangchote, N. Laosiripojana, *Mater. Res. Bull.* **2017**, 95, 546-555.

[10] J. Payormhorm, S. Chuangchote, K. Kiatkittipong, S. Chiarakorn, N. Laosiripojana, *Mater. Chem. Phys.* **2017**, 196, 29-36.

[11] K. Roongraung, S. Chuangchote, N. Laosiripojana, *Catalysts* **2020**, 10, 423.

[12] J. C. Colmenares, A. Magdziarz, A. Bielejewska, *Bioresour. Technol.* **2011**, 102, 11254-11257.

[13] M. Bellardita, E. I. García-López, G. Marcì, B. Megna, F. R. Pomilla, L. Palmisano, *RSC Adv.* **2015**, 5, 59037-59047.

[14] J. Yin, Q. Zhang, C. Yang, B. Zhang, K. Deng, *Catal. Sci. Technol.* **2020**, 10, 2231-2241.

[15] K. Roongraung, S. Chuangchote, N. Laosiripojana, T. Sagawa, *ACS Omega* **2020**, 5, 5862-5872.

[16] N. Suriyachai, S. Chuangchote, N. Laosiripojana, V. Champreda, T. Sagawa, *ACS Omega* **2020**, 5, 20373-20381.

[17] J. Kaewsaenee, M. T. Singhaset, K. Roongraung, P. Kemacheevakul, S. Chuangchote, *ACS Omega* **2023**, 8, 43664-43673.

[18] M. Cheng, Q. Zhang, C. Yang, B. Zhang, K. Deng, *Catal. Sci. Technol.* **2019**, 9, 6909-6919.

[19] Q. Zhang, Y. Ge, C. Yang, B. Zhang, K. Deng, *Green Chem.* **2019**, 21, 5019-5029.

[20] Q. Zhang, C. Yang, B. Zhang, K. Deng, *ACS Sustainable Chem. Eng.* **2021**, 9, 2057-2066.

[21] R. Chen, C. Yang, Q. Zhang, B. Zhang, K. Deng, *J. Catal.* **2019**, 374, 297-305.

[22] O. E. Jaime-Acuña, J. L. Zamora, O. Raymond-Herrera, *Sustain. Chem. Pharm.* **2021**, 19, 100351.

[23] M. Omri, F. Sauvage, Y. Busby, M. Becuwe, G. Pourceau, A. Wadouachi, *ACS Catal.* **2018**, 8, 1635-1639.

[24] S. Barman, R. Chakraborty, *J. Environ. Chem. Eng.* **2021**, 9, 106736.

[25] P. G. Kargar, M. Niakan, B. Maleki, R. S. Zabibah, M. A. Apoorvari, S. S. Ashrafi, S. Arghavani, S. Zhou, *ACS Sustainable Chem. Eng.* **2024**, 12, 18149-18160.

[26] P. Han, T. Tana, S. Sarina, E. R. Waclawik, C. Chen, J. Jia, K. Li, Y. Fang, Y. Huang, W. Doherty, S. E. Bottle, J. Zhao, H. Y. Zhu, *Appl. Catal., B* **2021**, 296, 120340.

[27] Y. Shi, T. Tana, W. Yang, Z. Zhou, H. Yong Zhu, A. C. Bissember, J. Huang, P. Han, S. Sarina, *Angew. Chem. Int. Ed.* **2024**, 63, e202409456.

[28] T. Tana, P. Han, A. J. Brock, X. Mao, S. Sarina, E. R. Waclawik, A. Du, S. E. Bottle, H. Y. Zhu, *Nat. Commun.* **2023**, 14, 4609.

[29] M. Nayebi, A. Faraji, A. Bahadoran, Z. J. Othman, S. Arghavani, P. G. Kargar, S. M. Sajjadinezhad, R. S. Varma, *ACS Appl. Mater. Interfaces* **2023**, 15, 8054-8065.

[30] J. Wang, H. Zhao, P. Liu, N. Yasri, N. Zhong, M. G. Kibria, J. Hu, *J. Energy Chem.* **2022**, 74, 324-331.

[31] X. Du, H. Zhang, T. Yao, S. Dong, L. Jing, J. Hu, *Surf. Interf.* **2024**, 48, 104283.

[32] U. Nwosu, H. Zhao, M. Kibria, J. Hu, *ACS Sustainable Chem. Eng.* **2022**, 10, 5867-5874.

[33] R. Chong, J. Li, Y. Ma, B. Zhang, H. Han, C. Li, *J. Catal.* **2014**, 314, 101-108.

[34] H. Zhao, P. Liu, X. Wu, A. Wang, D. Zheng, S. Wang, Z. Chen, S. Larter, Y. Li, B. L. Su, M. G. Kibria, J. Hu, *Appl. Catal., B* **2021**, 291, 120055.

[35] B. Zhou, J. Song, T. Wu, H. Liu, C. Xie, G. Yang, B. Han, *Green Chem.* **2016**, 18, 3852-3857.

[36] J. Wang, Q. Zhao, P. Kumar, H. Zhao, L. Jing, D. Di Tommaso, R. Crespo-Otero, M. G. Kibria, J. Hu, *ACS Catal.* **2024**, 14, 3376-3386.

[37] J. Ma, Y. Li, D. Jin, Z. Ali, G. Jiao, J. Zhang, S. Wang, R. Sun, *Green Chem.* **2020**, 22, 6384-6392.

[38] J. Huang, Y. Ding, J. Li, Z. Hu, S. Saravanamurugan, J. Wang, Y. Su, S. Yang, H. Li, *Carbon Energy* **2025**, 7, 161407.

[39] Q. Z. Luo, J. S. Huang, T. Y. Liu, J. L. Yuan, A. S. Belousov, S. K. Li, S. Yang, H. Li, *Rare Met.* **2025**, 44, 3218-3233.

[40] A. Srikhaow, C. Chuaicham, J. Trakulmututa, K. Shu, K. Sasaki, *Sustain. Energy Fuels* **2024**, 8, 3065-3076.

[41] A. Srikhaow, C. Chuaicham, S. Shenoy, J. Trakulmututa, K. Sasaki, *Chem. Eng. J.* **2023**, 473, 145167.

[42] Y. Ding, Y. Cao, D. Chen, J. Li, H. Wu, Y. Meng, J. Huang, J. Yuan, Y. Su, J. Wang, H. Li, *Chem. Eng. J.* **2023**, 452, 139687.

[43] J. Ma, X. Li, Y. Li, G. Jiao, H. Su, D. Xiao, S. Zhai, R. Sun, *Adv. Powder Mater.* **2022**, 1, 100058.

[44] E. Wang, A. Mahmood, S. G. Chen, W. Sun, T. Muhmood, X. Yang, Z. Chen, *ACS Catal.* **2022**, 12, 11206-11215.

[45] R. Zou, Z. Chen, L. Zhong, W. Yang, T. Li, J. Gan, Y. Yang, Z. Chen, H. Lai, X. Li, C. Liu, S. Admassie, E. I. Iwuoha, J. Lu, X. Peng, *Adv. Funct. Mater.* **2023**, 33, 2301311.

[46] R. Cui, J. Ma, K. Liu, Z. Ali, J. Zhang, Z. Liu, X. Li, S. Yao, R. Sun, *Mol. Catal.* **2022**, 531, 112653.

[47] J. Trakulmututa, C. Chuaicham, A. Srikhaow, K. Sasaki, *Sustain. Mater. Technol.* **2024**, 42, e01129.

[48] T. W. Wang, Z. W. Yin, Y. H. Guo, F. Y. Bai, J. Chen, W. Dong, J. Liu, Z. Y. Hu, L. Chen, Y. Li, B. L. Su, *CCS Chem.* **2023**, 5, 1773-1788.

[49] Y. Li, J. Ma, D. Jin, G. Jiao, X. Yang, K. Liu, J. Zhou, R. Sun, *Appl. Catal., B* **2021**, 291, 120123.

[50] D. Jin, G. Jiao, W. Ren, J. Zhou, J. Ma, R. Sun, *J. Mater. Chem. C* **2021**, 9, 16450-16458.

[51] Y. S. Shen, F. Y. Bai, K. Wei, X. L. Wang, J. Chen, Z. Y. Jiang, J. Liu, Z. Y. Hu, L. H. Chen, Y. Li, B. L. Su, *Appl. Surf. Sci.* **2023**, 626, 157237.

[52] H. Zhao, C. F. Li, X. Yong, P. Kumar, B. Palma, Z. Y. Hu, G. Van Tendeloo, S. Siahrostami, S. Larter, D. Zheng, S. Wang, Z. Chen, M. G. Kibria, J. Hu, *iScience* **2021**, 24, 102109.

[53] X. Ye, X. Shi, H. Zhong, T. Wang, J. Duo, B. Jin, F. Jin, *Green Chem.* **2022**, 24, 813-822.

[54] X. Yang, K. Liu, J. Ma, R. Sun, *Green Chem.* **2022**, 24, 5894-5903.

[55] J. Wang, X. Wang, H. Zhao, J. F. Van Humbeck, B. N. Richtik, M. R. Dolgos, A. Seifitokaldani, M. G. Kibria, J. Hu, *ACS Catal.* **2022**, 12, 14418-14428.

[56] Y. W. Han, L. Ye, T. J. Gong, Y. Fu, *Angew. Chem. Int. Ed.* **2023**, 62, e202306305.

[57] S. Song, J. Qu, P. Han, M. J. Hulsey, G. Zhang, Y. Wang, S. Wang, D. Chen, J. Lu, N. Yan, *Nat. Commun.* **2020**, 11, 4899.

[58] P. Li, B. Zhang, *ACS Catal.* **2024**, 14, 18345-18353.

[59] T. G. Lee, H. J. Kang, G. Bari, J. W. Park, H. W. Seo, B. H. An, H. J. Hwang, Y. S. Jun, *Chemosphere* **2021**, 283, 131174.

[60] A. Speltini, A. Scalabrini, F. Maraschi, M. Sturini, A. Pisanu, L. Malavasi, A. Profumo, *Int. J. Hydrogen Energy* **2018**, 43, 14925-14933.

[61] C. R. Thara, P. S. Walko, B. Mathew, *Renew. Energy* **2024**, 230, 120811.

[62] J. Zhang, X. Xu, Y. Liu, X. Duan, S. Wang, H. Sun, *Surf. Interf.* **2023**, 42, 103423.

[63] F. Ding, H. Yu, W. Liu, X. Zeng, S. Li, L. Chen, B. Li, J. Guo, C. Wu, *Mater. Des.* **2024**, 238, 112678.

[64] A. Speltini, L. Romani, D. Dondi, L. Malavasi, A. Profumo, *Catalysts* **2020**, 10, 1259.

[65] X. Jing, Y. Zhang, H. Chang, R. Qiu, W. Yang, H. Xie, W. Wang, M. Zhang, X. Lyu, Q. Liu, X. Wang, J. Crittenden, X. Lyu, *J. Environ. Chem. Eng.* **2024**, 12, 113040.

[66] H. Zhao, X. Ding, B. Zhang, Y. Li, C. Wang, *Sci. Bull.* **2017**, 62, 602-609.

[67] H. Zhang, H. Zhao, S. Zhai, R. Zhao, J. Wang, X. Cheng, H. S. Shiran, S. Larter, M. G. Kibria, J. Hu, *Appl. Catal., B* **2022**, 316, 121647.

[68] C. Shi, M. Eqi, J. Shi, Z. Huang, H. Qi, *J Colloid Interface Sci.* **2023**, 650, 1736-1748.

[69] N. Zhong, X. Yu, H. Zhao, J. Hu, I. D. Gates, *Catalysts* **2022**, 12, 819.

[70] C. G. Silva, M. J. Sampaio, R. R. N. Marques, L. A. Ferreira, P. B. Tavares, A. M. T. Silva, J. L. Faria, *Appl. Catal., B* **2015**, 178, 82-90.

[71] X. Fu, J. Long, X. Wang, D. Leung, Z. Ding, L. Wu, Z. Zhang, Z. Li, X. Fu, *Int. J. Hydrogen Energy* **2008**, 33, 6484-6491.

[72] M. Zhou, Y. Li, S. Peng, G. Lu, S. Li, *Catal. Commun.* **2012**, 18, 21-25.

[73] B. Zhou, J. Song, H. Zhou, T. Wu, B. Han, *Chem. Sci.* **2016**, 7, 463-468.

[74] P. Tkachenko, V. Volchek, A. Kurenkova, E. Gerasimov, P. Popovetskiy, I. Asanov, I. Yushina, E. Kozlova, D. Vasilchenko, *Int. J. Hydrogen Energy* **2023**, 48, 22366-22378.

[75] M. Bellardita, H. A. E. Nazer, V. Loddo, F. Parrino, A. M. Venezia, L. Palmisano, *Catal. Today* **2017**, 284, 92-99.

[76] G. Ramis, E. Bahadori, I. Rossetti, *Int. J. Hydrogen Energy* **2021**, 46, 12105-12116.

[77] G. Iervolino, V. Vaiano, J. J. Murcia, L. Rizzo, G. Ventre, G. Pepe, P. Campiglia, M. C. Hidalgo, J. A. Navío, D. Sannino, *J. Catal.* **2016**, 339, 47-56.

[78] A. E. Lara Sandoval, J. Serafin, J. J. Murcia Mesa, H. A. Rojas Sarmiento, J. S. Hernandez Niño, J. Llorca, J. A. Navío Santos, M. C. Hidalgo Lõpez, *Fuel* **2024**, 363, 130932.

[79] M. Eqi, C. Shi, J. Xie, F. Kang, H. Qi, X. Tan, Z. Huang, J. Liu, J. Guo, *Adv. Compos. Hybrid Mater.* **2022**, 6, 5.

[80] C. Ma, Y. Li, H. Zhang, Y. Chen, C. Lu, J. Wang, *Chem. Eng. J.* **2015**, 273, 277-285.

[81] H. Zhao, X. Yu, C. F. Li, W. Yu, A. Wang, Z. Y. Hu, S. Larter, Y. Li, M. Golam Kibria, J. Hu, *J. Energy Chem.* **2022**, 64, 201-208.

[82] Y. You, S. Chen, J. Zhao, J. Lin, D. Wen, P. Sha, L. Li, D. Bu, S. Huang, *Adv. Mater.* **2023**, 36, 2307962.

[83] K. Roongraung, A. Cherevan, D. Eder, S. Chuangchote, *Catal. Sci. Technol.* **2023**, 13, 5556-5566.

[84] F. Kang, C. Shi, Y. Zhu, M. Eqi, J. Shi, M. Teng, Z. Huang, C. Si, F. Jiang, J. Hu, *J. Energy Chem.* **2023**, 79, 158-167.

[85] X. Wang, X. Zheng, H. Han, Y. Fan, S. Zhang, S. Meng, S. Chen, *J. Solid State Chem.* **2020**, 289, 121495.

[86] C. Li, H. Wang, J. Ming, M. Liu, P. Fang, *Int. J. Hydrogen Energy* **2017**, 42, 16968-16978.

[87] S. Q. Peng, Y. J. Peng, Y. X. Li, G. X. Lu, S. B. Li, *Res. Chem. Intermed.* **2009**, 35, 739-749.

[88] Y. Li, D. Gao, S. Peng, G. Lu, S. Li, *Int. J. Hydrogen Energy* **2011**, 36, 4291-4297.

[89] A. Y. Kurenkova, D. V. Markovskaya, E. Y. Gerasimov, I. P. Prosvirin, S. V. Cherepanova, E. A. Kozlova, *Int. J. Hydrogen Energy* **2020**, 45, 30165-30177.

[90] Y. Li, J. Wang, S. Peng, G. Lu, S. Li, *Int. J. Hydrogen Energy* **2010**, 35, 7116-7126.

[91] K. O. Potapenko, E. Y. Gerasimov, S. V. Cherepanova, A. A. Saraev, E. A. Kozlova, *Materials* **2022**, 15, 8026.

[92] J. Zhang, J. Ma, R. Cui, W. Ling, M. Hong, R. Sun, *Chem. Eng. J.* **2025**, 503, 158427.

[93] G. Iervolino, V. Vaiano, D. Sannino, L. Rizzo, A. Galluzzi, M. Polichetti, G. Pepe, P. Campiglia, *Int. J. Hydrogen Energy* **2018**, 43, 2184-2196.

[94] G. Iervolino, V. Vaiano, D. Sannino, L. Rizzo, P. Ciambelli, *Int. J. Hydrogen Energy* **2016**, 41, 959-966.

[95] G. Iervolino, V. Vaiano, D. Sannino, F. Puga, J. A. Navío, M. C. Hidalgo, *Catalysts* **2021**, 11, 1558.

[96] G. Iervolino, V. Vaiano, D. Sannino, L. Rizzo, V. Palma, *Appl. Catal., B* **2017**, 207, 182-194.

[97] D. Jing, M. Liu, J. Shi, W. Tang, L. Guo, *Catal. Commun.* **2010**, 12, 264-267.

[98] L. Madriz, J. Tatá, D. Carvajal, O. Núñez, B. R. Scharifker, J. Mostany, C. Borrás, F. M. Cabrerizo, R. Vargas, *Renew. Energy* **2020**, 152, 974-983.

[99] M. Umair, A. M. Djaballah, M. Bellardita, R. Bagtache, L. Palmisano, M. Trari, *Adv. Sustainable Syst.* **2024**, 8, 2400298.

[100] Y. Tian, Y. Hong, B. Chen, K. Zhang, D. Hong, X. Lin, J. Shi, *Int. J. Hydrogen Energy* **2024**, 51, 475-487.

[101] X. Zhang, *Catal.s Lett.* **2014**, 144, 1253-1257.

[102] X. Fu, X. Wang, D. Y. C. Leung, W. Xue, Z. Ding, H. Huang, X. Fu, *Catal. Commun.* **2010**, 12, 184-187.

[103] S. A. Kurnosenko, V. V. Voytovich, O. I. Silyukov, I. A. Rodionov, I. A. Zvereva, *Nanomaterials* **2022**, 12, 2717.

[104] F. Y. Bai, J. R. Han, J. Chen, Y. Yuan, K. Wei, Y. S. Shen, Y. F. Huang, H. Zhao, J. Liu, Z. Y. Hu, Y. Li, B. L. Su, *J. Colloid Interface Sci.* **2023**, 638, 173-183.

[105] L. Guan, G. Cheng, B. Tan, S. Jin, *Chem. Commun.* **2021**, 57, 5147-5150.

[106] V. C. Nguyen, N. J. Ke, L. D. Nam, B. S. Nguyen, Y. K. Xiao, Y. L. Lee, H. Teng, *J. Mater. Chem. A* **2019**, 7, 8384-8393.

[107] B. Jin, G. Yao, X. Wang, K. Ding, F. Jin, *ACS Sustainable Chem. Eng.* **2017**, 5, 6377-6381.

[108] Y. Zhu, R. Tan, C. Yang, B. Zhang, K. Deng, D. Tang, D. Ding, *Mol. Catal.* **2024**, 554, 113818.

[109] C. Shi, Y. An, G. Gao, J. Xue, H. Algadi, Z. Huang, Z. Guo, *ACS Sustainable Chem. Eng.* **2024**, 12, 2538-2549.

[110] E. Lam, M. Miller, S. Linley, R. R. Manuel, I. A. C. Pereira, E. Reisner, *Angew Chem Int Ed Engl* **2023**, 62, e202215894.

[111] K. Imamura, K. Ikeuchi, Y. Sakamoto, Y. Aono, T. Oto, A. Onda, *RSC Adv.* **2021**, 11, 32300-32304.

[112] P. Ren, Z. Gao, T. Montini, Z. Zhao, N. Ta, Y. Huang, N. Luo, E. Fonda, P. Fornasiero, F. Wang, *Joule* **2023**, 7, 333-349.

[113] Y. Ge, Q. Zhang, C. Yang, B. Zhang, K. Deng, *Appl. Catal., A* **2021**, 623, 118265.

[114] H. T. Ren, C. C. Cai, P. Y. Zhu, C. Wang, S. H. Wu, Y. Liu, X. Han, *Langmuir* **2024**, 40, 7384-7394.

[115] R. A. Borges, M. F. Pedrosa, Y. A. Manrique, C. G. Silva, A. M. T. Silva, J. L. Faria, M. J. Sampaio, *Chem. Eng. J.* **2023**, 470, 144066.

[116] P. Zhang, D. Sun, A. Cho, S. Weon, S. Lee, J. Lee, J. W. Han, D. P. Kim, W. Choi, *Nat. Commun.* **2019**, 10, 940.

[117] Z. Zhang, M. Wang, H. Zhou, F. Wang, *J. Am. Chem. Soc.* **2021**, 143, 6533-6541.

[118] Z. Chen, H. Zhou, F. Kong, Z. Dou, J. Yang, M. Wang, *Adv. Funct. Mater.* **2024**, 35, 2418154.

[119] H. Zhou, M. Wang, F. Wang, *Chem* **2022**, 8, 465-479.

[120] M. Wang, M. Liu, J. Lu, F. Wang, *Nat. Commun.* **2020**, 11, 1083.
